# Supplementary material for: A new system of phosphorus and calcium requirements for lactating dairy cows
Source: PLoS One. 2024 Aug 29;19(8):e0308889. doi: 10.1371/journal.pone.0308889 (PMC11361663; doi:10.1371/journal.pone.0308889)
Supplement: S1 File — (DOCX) [file pone.0308889.s001.docx]

**Supporting information S1**

**Publications used to development the phosphorus and calcium requirement system for lactating dairy cows**

Berry, N.R., Jewell, P.L., Sutter, F., Edwards, P.J., Kreuzer, M., 2001. Effect of concentrate on nitrogen turnover and excretion of P, K, Na, Ca and Mg in lactating cows rotationally grazed at high altitude. Livestock Production Science 71, 261-275.

Bjelland, D.W., Weigel, K.A., Hoffman, P.C., Esser, N.M., Coblentz, W.K., 2011. The effect of feeding dairy heifers diets with and without supplemental phosphorus on growth, reproductive efficiency, health, and lactation performance1. Journal of Dairy Science 94, 6233-6242.

Borucki Castro, S.I., Phillip, L.E., Girard, V., Tremblay, A., 2004. Altering Dietary Cation-Anion Difference in Lactating Dairy Cows to Reduce Phosphorus Excretion to the Environment. Journal of Dairy Science 87, 1751-1757.

Brask-Pedersen, D.N., Glitsø, L.V., Skov, L.K., Lund, P., Sehested, J., 2013. Effect of exogenous phytase on degradation of inositol phosphate in dairy cows. Journal of Dairy Science 96, 1691-1700.

Brintrup, R., Mooren, T., Meyer, U., Spiekers, H., Pfeffer, E., 1993. Effects of 2 levels of phosphorus intake on performance and fecal phosphorus excretion of dairy-cows. Journal of Animal Physiology and Animal Nutrition-Zeitschrift Fur Tierphysiologie Tierernahrung Und Futtermittelkunde 69, 29-36.

Conrad, H.R., Hansard, S.L., Hibbs, J.W., 1956. Studies on Milk Fever in Dairy Cows. V. The Effect of Massive Oral Doses of Vitamin D on Absorption, Excretion, Retention and Blood Levels of Calcium and Phosphorus1. Journal of Dairy Science 39, 1697-1705.

Dann, H.M., Grant, R.J., Cotanch, K.W., Thomas, E.D., Ballard, C.S., Rice, R., 2008. Comparison of brown midrib sorghum-sudangrass with corn silage on lactational performance and nutrient digestibility in Holstein dairy cows. Journal of Dairy Science 91, 663-672.

Delaquis, A.M., Block, E., 1995. The effects of changing ration ingredients on acid-base status, renal-function, and macromineral metabolism. Journal of Dairy Science 78, 2024-2039.

Ekelund, A., Sporndly, R., Holtenius, K., 2006. Influence of low phosphorus intake during early lactation on apparent digestibility of phosphorus and bone metabolism in dairy cows. Livestock Science 99, 227-236.

Ekelund, A., Sporndly, R., Valk, H., Murphy, M., 2003. Influence of feeding various phosphorus sources on apparent digestibility of phosphorus in dairy cows. Animal Feed Science and Technology 109, 95-104.

Feng, X., Knowlton, K.F., Dietrich, A.D., Duncan, S., 2013. Effect of abomasal ferrous lactate infusion on phosphorus absorption in lactating dairy cows. Journal of Dairy Science 96, 4586-4591.

Ferris, C.P., Patterson, D.C., McCoy, M.A., Kilpatrick, D.J., 2010. Effect of offering dairy cows diets differing in phosphorus concentration over four successive lactations: 1. Food intake, milk production, tissue changes and blood metabolites. Animal 4, 545-559.

Guyton, A.D., McKinney, J.M., Knowlton, K.F., 2003. The Effect of Steam-Flaked or Dry Ground Corn and Supplemental Phytic Acid on Phosphorus Partitioning and Ruminal Phytase Activity in Lactating Cows. Journal of Dairy Science 86, 3972-3982.

Giagnoni, G.; Lund, P., Sehested, J., Johansen, M. 2021. Effect of exogenous dietary phytase and concentrate mixtures based on faba beans, rapeseed meal or soybean meal as main protein source on phytate and total phosphorus excretion in dairy cows. Animal Feed Science and eTecnhology 276:114913

Guo, Y.Q., Tong B.X., Wu, Z.G., Ma, W.Q., Ma, L. 2019 Diatery manipulation to reduce nitrogen and phosphorus excretion by dairt cows. Livestock Science. 228:61-66.

Haese, E., Muller, K., Steingass, H., Schollenberger, M., Rodehutscord, M., 2014. Effects of mineral and rapeseed phosphorus supplementation on phytate degradation in dairy cows. Archives of Animal Nutrition 68, 478-491.

Herrera, D., Harris, W.G., Nair, V.D., Josan, M., Staples, C.R., 2010. Effect of dietary modifications of calcium and magnesium on reducing solubility of phosphorus in feces from lactating dairy cows. Journal of Dairy Science 93, 2598-2611.

Hibbs, J.W., Conrad, H.R., 1983. The relation of calcium and phosphorus intake and digestion and the effects of vitamin D feeding on the utilization of calcium and phosphorus by lactating dairy cows.

Jarrett, J.P., Wilson, J.W., Ray, P.P., Knowlton, K.F., 2014. The effects of forage particle length and exogenous phytase inclusion on phosphorus digestion and absorption in lactating cows. Journal of Dairy Science 97, 411-418.

Kamiya, Y., Kamiya, M., Tanaka, M., 2006. The effect of prepartum diet on nitrogen and major mineral balance of dairy cows during parturition in summer. Asian-Australasian Journal of Animal Sciences 19, 1415-1421.

Kamiya, Y., Kamiya, M., Tanaka, M., 2010. The effect of high ambient temperature on Ca, P and Mg balance and bone turnover in high-yielding dairy cows. Animal Science Journal 81, 482-486.

Kebreab, E., France, J., Sutton, J.D., Crompton, L.A., Beever, D.E., 2005. Effect of energy and protein supplementation on phosphorus utilization in lactating dairy cows. Journal of Animal and Feed Sciences 14, 63-77.

Kincaid, R.L., Garikipati, D.K., Nennich, T.D., Harrison, J.H., 2005. Effect of grain source and exogenous phytase on phosphorus digestibility in dairy cows. Journal of Dairy Science 88, 2893-2902.

Knowlton, K.F., Herbein, J.H., 2002. Phosphorus partitioning during early lactation in dairy cows fed diets varying in phosphorus content. Journal of Dairy Science 85, 1227-1236.

Knowlton, K.F., Herbein, J.H., Meister-Weisbarth, M.A., Wark, W.A., 2001. Nitrogen and Phosphorus Partitioning in Lactating Holstein Cows Fed Different Sources of Dietary Protein and Phosphorus. Journal of Dairy Science 84, 1210-1217.

Knowlton, K.F., McKinney, J.M., Cobb, C., 2002. Effect of a Direct-Fed Fibrolytic Enzyme Formulation on Nutrient Intake, Partitioning, and Excretion in Early and Late Lactation Holstein Cows. Journal of Dairy Science 85, 3328-3335.

Knowlton, K.F., Parsons, C.M., Cobb, C.W., Wilson, K.F., 2005. Exogenous Phytase Plus Cellulase and Phosphorus Excretion in Lactating Dairy Cows. The Professional Animal Scientist 21, 212-216.

Knowlton, K.F., Taylor, M.S., Hill, S.R., Cobb, C., Wilson, K.F., 2007. Manure nutrient excretion by lactating cows fed exogenous phytase and cellulase. Journal of Dairy Science 90, 4356-4360.

Kojima, H., Nonaka, K., Oshita, T., Kozakai, T., Hirooka, H., 2005. Effects of feeding and animal performance on nitrogen, phosphorus and potassium excretion by Holstein cows. Animal Science Journal 76, 139-145.

Martz, F.A., Belo, A.T., Weiss, M.F., Belyea, R.L., Goff, J.P., 1990. True Absorption of Calcium and Phosphorus from Alfalfa and Corn Silage When Fed to Lactating Cows. Journal of Dairy Science 73, 1288-1295.

Miller, R.C., 1926. A Study of Calcium and Phosphorus Balances with Dairy Cattle. Journal of Dairy Science 9, 78-92.

Monroe, C.F., 1924. The Metabolism of Calcium, Magnesium, Phosphorus and Sulfur in Dairy Cows Fed High and Low Protein Rations. Journal of Dairy Science 7, 58-73.

Moreira, V.R., Zeringue, L.K., Williams, C.C., Leonardi, C., McCormick, M.E., 2009. Influence of calcium and phosphorus feeding on markers of bone metabolism in transition cows. Journal of Dairy Science 92, 5189-5198.

Morse, D., Head, H.H., Wilcox, C.J., Vanhorn, H.H., Hissem, C.D., Harris, B., 1992. effects of concentration of dietary phosphorus on amount and route of excretion. Journal of Dairy Science 75, 3039-3049.

Muller, C.B.M., B. Kihla. 2021. Holstein dairy cowswith high phosphorus utilization efficiency fed a low phosphorous diet secreted less phosphorus with urine but more with milk and feces. Science of the Total Environment 788:147813

Myers, Z.H., Beede, D.K., 2009. Evaluating estimates of phosphorus maintenance requirement of lactating Holstein cows with different dry matter intakes. Journal of Dairy Science 92, 708-719.

O'Rourke, E.M., Michal, J., Kincaid, R.L., Harrison, J.H., 2007. Use of Fecal Samples as a Tool to Monitor Phosphorus Excretion in Lactating Dairy Cows. The Professional Animal Scientist 23, 536-540.

Odongo, N.E., McKnight, D., KoekKoek, A., Fisher, J.W., Sharpe, F., Kebreab, E., Frances, J., McBride, B.W., 2007. Long-term effects of feeding diets without mineral phosphorus supplementation on the performance and phosphorus excretion in high-yielding dairy cows. Canadian Journal of Animal Science 87, 639-646.

Puggaard, L., Kristensen, N.B., Sehested, J., 2011. Effect of decreasing dietary phosphorus supply on net recycling of inorganic phosphate in lactating dairy cows. Journal of Dairy Science 94, 1420-1429.

Puggaard, L., Lund, P., Sehested, J., 2013. Effect of feed forage particle size and dietary urea on excretion of phosphorus in lactating dairy cows. Livestock Science 158, 50-56.

Ray, P.P., Jarrett, J., Knowlton, K.F., 2013. Effect of dietary phytate on phosphorus digestibility in dairy cows. Journal of Dairy Science 96, 1156-1163.

Reid, M., O'Donovan, M., Elliott, C.T., Bailey, J.S., Watson, C.J., Lalor, S.T.J., Corrigan, B., Fenelon, M.A., Lewis, E., 2015. The effect of dietary crude protein and phosphorus on grass-fed dairy cow production, nutrient status, and milk heat stability. Journal of Dairy Science 98, 517-531.

Rindsig, R.B., Schultz, L.H., 1970. Effect of Bentonite on Nitrogen and Mineral Balances and Ration Digestibility of High-Grain Rations Fed to Lactating Dairy Cows1. Journal of Dairy Science 53, 888-892.

Ruiz, T.M., Sotomayor-Ramírez, D., Torres-Meléndez, C., Martínez-Rodriguez, G.A., 2016. Phosphorus mass cycling and balance in dairy farms: Case studies in Puerto Rico. Agriculture, Ecosystems & Environment 220, 115-124.

Salazar, J.A.E., Ferguson, J.D., Beegle, D.B., Remsburg, D.W., Wu, Z., 2013. Body phosphorus mobilization and deposition during lactation in dairy cows. Journal of Animal Physiology and Animal Nutrition 97, 502-514.

Shore, K.V., Odongo, N.E., Mutsvangwa, T., Widowski, T.M., Cant, J.P., Bettger, W.J., McBride, B.W., 2005. Phosphorus status of lactating dairy cows fed total mixed rations containing 0.24% vs. 0.36% phosphorus. Canadian Journal of Animal Science 85, 409-412.

Spiekers, H., Brintrup, R., Balmelli, M., Pfeffer, E., 1993. Influence of dry-matter intake on fecal phosphorus losses in dairy-cows fed rations low in phosphorus. Journal of Animal Physiology and Animal Nutrition-Zeitschrift Fur Tierphysiologie Tierernahrung Und Futtermittelkunde 69, 37-43.

St-Pierre, N.R., Bouchard, R., St-Laurent, G.J., Vinet, C., Roy, G.L., 1983. Effects of Stage of Maturity and Frost on Nutritive Value of Corn Silage for Lactating Dairy Cows1. Journal of Dairy Science 66, 1466-1473.

St. Pierre, N.R., Bouchard, R., St. Laurent, G., Roy, G.L., Vinet, C., 1987. Performance of Lactating Dairy Cows Fed Silage from Corn of Varying Maturities. Journal of Dairy Science 70, 108-115.

Taylor, M.S., Knowlton, K.F., McGilliard, M.L., Swecker, W.S., Ferguson, J.D., Wu, Z., Hanigan, M.D., 2009. Dietary calcium has little effect on mineral balance and bone mineral metabolism through twenty weeks of lactation in Holstein cows. Journal of Dairy Science 92, 223-237.

Valk, H., Sebek, L.B.J., Beynen, A.C., 2002. Influence of phosphorus intake on excretion and blood plasma and saliva concentrations of phosphorus in dairy cows. Journal of Dairy Science 85, 2642-2649.

Winter, L., Meyer, U., von Soosten, D., Gorniak, M., Lebzien, P., Danicke, S., 2015. Effect of phytase supplementation on rumen fermentation characteristics and phosphorus balance in lactating dairy cows. Italian Journal of Animal Science 14:53-60

Wondater, W.B., Ayanie, T.D. 2023. Supplementing dairy feed by dicalcium phosphate and effect on dry matter intake, digestibility, milk composition, and blood mineral balances in crossbred dairy cows. Plos One. 18(11): e0282879

Wu, Z., 2005. Utilization of Phosphorus in Lactating Cows Fed Varying Amounts of Phosphorus and Sources of Fiber. Journal of Dairy Science 88, 2850-2859.

Wu, Z., Satter, L.D., Blohowiak, A.J., Stauffacher, R.H., Wilson, J.H., 2001. Milk production, estimated phosphorus excretion, and bone characteristics of dairy cows fed different amounts of phosphorus for two or three years. Journal of Dairy Science 84, 1738-1748.

Wu, Z., Satter, L.D., Sojo, R., 2000. Milk Production, Reproductive Performance, and Fecal Excretion of Phosphorus by Dairy Cows Fed Three Amounts of Phosphorus1. Journal of Dairy Science 83, 1028-1041.

Wu, Z., Tallam, S.K., Ishler, V.A., Archibald, D.D., 2003. Utilization of Phosphorus in Lactating Cows Fed Varying Amounts of Phosphorus and Forage. Journal of Dairy Science 86, 3300-3308.
