# Supplementary material for: A new system of phosphorus and calcium requirements for lactating dairy cows
Source: PLoS One. 2024 Aug 29;19(8):e0308889. doi: 10.1371/journal.pone.0308889 (PMC11361663; doi:10.1371/journal.pone.0308889)
Supplement: S2 File — (DOCX) [file pone.0308889.s002.docx]

**Supporting information S2**

**S2.1 Final Dataset, codes used to derive the P excretion nonlinear model, quantify study variance, and cross-validation procedure (Table 2; Figures 2 and 3; and Supplementary S3.1)**

**Abbreviations:**

Obs: observations ID

Study: Study ID

Model: Mixed (1) or Fixed (2)

Fold: FoldID (1 to 5)

PfecalurinaryBW075 = P fecal + urinary excretion (g/BW^0.75^/d)

PintakeBW075 = P intake (g/BW^0.75^/d)

InvTruSEMPfecalPolMis = normalized inverse of SEM P fecal excretion

data P_COMPLETE_FINALMODEL;

input Model Obs Study FOLDS PfecalurinaryBW075 PintakeBW075 InvTruSEMPfecalPolMis;

cards;

1 9 3 1 0.505768414 0.664019852 0.647668394

1 10 3 1 0.526444481 0.70855292 0.647668394

1 11 3 1 0.49543038 0.699805353 0.647668394

1 12 3 1 0.500201781 0.672767419 0.647668394

2 65 17 1 0.28182751 0.455013771 1.218687352

2 66 17 1 0.352041778 0.55384749 2.1036865

2 67 17 1 0.345864026 0.504737903 0.494985059

2 68 17 1 0.461937682 0.583028142 0.292082093

2 86 23 1 0.347295495 0.581875487 0.409999225

2 87 23 1 0.325131564 0.60335538 0.981720367

2 88 23 1 0.316383018 0.570016339 0.690272133

2 89 23 1 0.328112506 0.616712685 0.435245483

2 96 25 1 0.378097072 0.512131484 0.31387152

2 97 25 1 0.357297776 0.559986203 0.209620007

2 120 33 1 0.331390337 0.548494268 1.244434268

2 121 33 1 0.326440609 0.538836261 1.244434268

2 122 33 1 0.331953721 0.536824177 1.244434268

2 123 33 1 0.349981999 0.573846534 1.244434268

2 124 33 1 0.34382502 0.564188528 1.244434268

2 125 33 1 0.349338132 0.562176443 1.244434268

2 161 43 1 0.419510514 0.521543323 1

1 176 47 1 0.122285401 0.231053393 2.233339289

1 177 47 1 0.16539853 0.360309879 2.233339289

1 178 47 1 0.217035321 0.535808516 2.233339289

1 228 64 1 0.167234386 0.296144225 2.158894646

1 229 64 1 0.157653249 0.296144225 2.158894646

1 2 1 2 0.207080663 0.32748785 1.750455118

1 3 1 2 0.218325233 0.283986957 1.750455118

1 5 1 2 0.187464223 0.251976867 1.799078872

1 6 1 2 0.229651881 0.337337108 1.799078872

2 78 20 2 0.276102418 0.444549729 1.06451606

2 98 26 2 0.23320876 0.501710241 0.88354833

2 99 26 2 0.163625592 0.546374174 1.318728851

2 100 26 2 0.182263161 0.511198295 0.48018931

2 101 26 2 0.193042881 0.482378207 1.436663951

1 112 31 2 0.293378764 0.547052132 0.925240563

1 113 31 2 0.299520717 0.55830058 1.03626943

2 159 41 2 0.345129028 0.485046645 1

2 160 42 2 0.474888549 0.653767017 1

2 164 46 2 0.453253097 0.501201361 1

2 165 46 2 0.277832621 0.501201361 1

2 166 46 2 0.285517709 0.501201361 1

2 167 46 2 0.352511624 0.501201361 1

2 168 46 2 0.376485756 0.684975193 1

2 169 46 2 0.35827544 0.684975193 1

2 170 46 2 0.391271196 0.684975193 1

2 171 46 2 0.422930415 0.684975193 1

2 172 46 2 0.431200238 0.935575874 1

2 173 46 2 0.521082348 0.935575874 1

2 174 46 2 0.595677818 0.935575874 1

2 175 46 2 0.667349612 0.935575874 1

2 206 58 2 0.169289194 0.304063115 2.1036865

2 207 58 2 0.112605126 0.178015456 2.1036865

2 57 15 3 0.291686538 0.41179276 1.328644105

2 58 15 3 0.296783473 0.45222145 0.441774165

2 59 15 3 0.352050655 0.45636196 0.50488476

2 60 15 3 0.344794632 0.548240835 1.17806444

2 79 21 3 0.276102418 0.444549729 1.06451606

2 80 21 3 0.269912312 0.357353212 0.654480245

2 81 21 3 0.23738749 0.371046967 0.4207373

2 92 24 3 0.32339724 0.428136914 0.312760471

2 93 24 3 0.446229171 0.699946812 0.302067805

2 94 24 3 0.38652876 0.534637911 0.218160082

2 95 24 3 0.272021518 0.37221265 0.191037477

2 102 27 3 0.134062215 0.265497998 1.125539274

2 103 27 3 0.177870713 0.276738225 1.6829492

2 115 32 3 0.417935842 0.57408307 1.425077952

2 117 32 3 0.412885258 0.572399542 1.425077952

2 118 32 3 0.341251139 0.451185521 1.425077952

1 130 35 3 0.402204225 0.788886573 0.835701153

1 131 35 3 0.360422634 0.631442188 0.835701153

1 132 35 3 0.402399342 0.701445955 0.835701153

1 133 35 3 0.345280454 0.627862029 0.835701153

1 134 36 3 0.703397669 0.998058521 0.681756204

1 135 36 3 0.701069749 0.994029249 0.681756204

1 190 53 3 0.417782565 0.78446942 0.785052599

1 191 53 3 0.585697092 0.843580143 0.785052599

1 192 53 3 0.650318136 0.989854134 0.785052599

1 193 53 3 0.512059158 0.934750918 0.785052599

2 26 8 4 0.134706395 0.419169681 0.694181129

2 27 8 4 0.134676005 0.417042523 0.694181129

2 69 18 4 0.380320122 0.567368806 0.690272133

2 70 18 4 0.332677954 0.549931982 0.591002227

2 71 18 4 0.288209535 0.458686021 0.550497402

2 72 18 4 0.331362822 0.51968255 0.706838664

2 90 24 4 0.31780181 0.599810242 0.821905423

1 106 29 4 0.547774318 0.735698336 1.102414287

1 107 29 4 0.523580212 0.757423656 1.102414287

1 108 29 4 0.596360034 0.768286316 1.102414287

1 109 29 4 0.562883291 0.731748278 1.102414287

1 138 37 4 0.3048317 0.643597034 0.454504136

1 139 37 4 0.306546737 0.57885815 0.454504136

1 140 37 4 0.371134435 0.762246103 0.454504136

1 141 37 4 0.380488499 0.738407726 0.454504136

1 142 37 4 0.381532421 0.745052582 0.454504136

1 144 37 4 0.751553801 1.203626632 0.404792746

1 145 37 4 0.858209478 1.226135519 0.404792746

1 146 37 4 0.716816304 1.246899674 0.404792746

1 147 37 4 0.751850301 1.192536574 0.404792746

1 150 37 4 0.958563189 1.365069018 0.404792746

1 151 37 4 0.952883208 1.374374635 0.404792746

1 152 37 4 0.997343624 1.480444814 0.404792746

2 156 39 4 0.391580316 0.616961947 1

2 162 44 4 0.245992675 0.325742997 1

2 163 45 4 0.309752355 0.498058472 1

2 61 16 5 0.298069837 0.522539437 0.758410584

2 62 16 5 0.359699621 0.549066586 0.58903222

2 63 16 5 0.242355842 0.585350974 1

2 64 16 5 0.296109559 0.579975198 1

2 73 19 5 0.252435807 0.401166961 0.353419332

2 74 19 5 0.268413487 0.42289428 1.17806444

2 75 19 5 0.232437418 0.365659284 0.58903222

2 76 19 5 0.265382551 0.396811245 1

2 82 22 5 0.371737591 0.74239924 0.88354833

2 84 22 5 0.387857067 0.719151645 0.411910643

2 110 30 5 0.376541101 0.705817628 0.519734312

2 111 30 5 0.313521669 0.573476823 0.519734312

2 157 40 5 0.13251226 0.248460488 2.1036865

2 158 40 5 0.171109581 0.375034699 2.1036865

1 188 52 5 0.180664989 0.396601877 1.497499176

1 189 52 5 0.257170236 0.556401798 1.497499176

2 208 59 5 0.134474123 0.52074463 0.930050874

2 209 59 5 0.137261084 0.499616121 0.930050874

2 210 59 5 0.169517996 0.556230925 0.930050874

2 211 59 5 0.202462683 0.671044438 0.930050874

2 213 60 5 0.203906213 0.595760762 1.963440734

2 214 60 5 0.190338046 0.571724354 1.963440734

2 215 60 5 0.185997928 0.553206422 1.963440734

2 216 60 5 0.238712801 0.59765068 1.963440734

2 217 60 5 0.159770799 0.572210412 1.963440734

;

proc means;

var Study FOLDS PfecalurinaryBW075 PintakeBW075 InvTruSEMPfecalPolMis;

run;

proc means;

var Study FOLDS PfecalurinaryBW075 PintakeBW075 InvTruSEMPfecalPolMis;

by FOLDS;

run;

PROC nLmixed ;

Title FINALMODEL_PExcretion;

parms a = 0.13 b = 1.40

ve= 0.0006 va= -35;

model PfecalurinaryBW075 ~ normal (a*exp(b*PintakeBW075)+ u1,ve);

random u1 ~ normal(0, va)Subject=Study;

replicate InvTruSEMPfecalPolMis;

run;

/*Variance Study*/

Proc mixed;

Title P Effect Pfecal x Study;

class study;

MODEL PfecalurinaryBW075 = PintakeBW075 Study*PintakeBW075/solution;

RANDOM Study;

WEIGHT InvTruSEMPfecalPolMis;

RUN;

Proc mixed;

Title P Effect Pfecal x Study;

MODEL PfecalurinaryBW075 = PintakeBW075 Study*PintakeBW075/solution;

RANDOM Study/solution;

WEIGHT InvTruSEMPfecalPolMis;

RUN;

Proc mixed method=type3;

Title Variance Study Pfecal;

class Study;

MODEL PfecalurinaryBW075 = PintakeBW075/solution;

RANDOM Study;

WEIGHT InvTruSEMPfecalPolMis;

RUN;

data KCROSSVALIDATIONRUN1;

input Model Obs Study FOLDS PfecalurinaryBW075 PintakeBW075 InvTruSEMPfecalPolMis;

cards;

1 2 1 2 0.207080663 0.32748785 1.750455118

1 3 1 2 0.218325233 0.283986957 1.750455118

1 5 1 2 0.187464223 0.251976867 1.799078872

1 6 1 2 0.229651881 0.337337108 1.799078872

2 78 20 2 0.276102418 0.444549729 1.06451606

2 98 26 2 0.23320876 0.501710241 0.88354833

2 99 26 2 0.163625592 0.546374174 1.318728851

2 100 26 2 0.182263161 0.511198295 0.48018931

2 101 26 2 0.193042881 0.482378207 1.436663951

1 112 31 2 0.293378764 0.547052132 0.925240563

1 113 31 2 0.299520717 0.55830058 1.03626943

2 159 41 2 0.345129028 0.485046645 1

2 160 42 2 0.474888549 0.653767017 1

2 164 46 2 0.453253097 0.501201361 1

2 165 46 2 0.277832621 0.501201361 1

2 166 46 2 0.285517709 0.501201361 1

2 167 46 2 0.352511624 0.501201361 1

2 168 46 2 0.376485756 0.684975193 1

2 169 46 2 0.35827544 0.684975193 1

2 170 46 2 0.391271196 0.684975193 1

2 171 46 2 0.422930415 0.684975193 1

2 172 46 2 0.431200238 0.935575874 1

2 173 46 2 0.521082348 0.935575874 1

2 174 46 2 0.595677818 0.935575874 1

2 175 46 2 0.667349612 0.935575874 1

2 206 58 2 0.169289194 0.304063115 2.1036865

2 207 58 2 0.112605126 0.178015456 2.1036865

2 57 15 3 0.291686538 0.41179276 1.328644105

2 58 15 3 0.296783473 0.45222145 0.441774165

2 59 15 3 0.352050655 0.45636196 0.50488476

2 60 15 3 0.344794632 0.548240835 1.17806444

2 79 21 3 0.276102418 0.444549729 1.06451606

2 80 21 3 0.269912312 0.357353212 0.654480245

2 81 21 3 0.23738749 0.371046967 0.4207373

2 92 24 3 0.32339724 0.428136914 0.312760471

2 93 24 3 0.446229171 0.699946812 0.302067805

2 94 24 3 0.38652876 0.534637911 0.218160082

2 95 24 3 0.272021518 0.37221265 0.191037477

2 102 27 3 0.134062215 0.265497998 1.125539274

2 103 27 3 0.177870713 0.276738225 1.6829492

2 115 32 3 0.417935842 0.57408307 1.425077952

2 117 32 3 0.412885258 0.572399542 1.425077952

2 118 32 3 0.341251139 0.451185521 1.425077952

1 130 35 3 0.402204225 0.788886573 0.835701153

1 131 35 3 0.360422634 0.631442188 0.835701153

1 132 35 3 0.402399342 0.701445955 0.835701153

1 133 35 3 0.345280454 0.627862029 0.835701153

1 134 36 3 0.703397669 0.998058521 0.681756204

1 135 36 3 0.701069749 0.994029249 0.681756204

1 190 53 3 0.417782565 0.78446942 0.785052599

1 191 53 3 0.585697092 0.843580143 0.785052599

1 192 53 3 0.650318136 0.989854134 0.785052599

1 193 53 3 0.512059158 0.934750918 0.785052599

2 26 8 4 0.134706395 0.419169681 0.694181129

2 27 8 4 0.134676005 0.417042523 0.694181129

2 69 18 4 0.380320122 0.567368806 0.690272133

2 70 18 4 0.332677954 0.549931982 0.591002227

2 71 18 4 0.288209535 0.458686021 0.550497402

2 72 18 4 0.331362822 0.51968255 0.706838664

2 90 24 4 0.31780181 0.599810242 0.821905423

1 106 29 4 0.547774318 0.735698336 1.102414287

1 107 29 4 0.523580212 0.757423656 1.102414287

1 108 29 4 0.596360034 0.768286316 1.102414287

1 109 29 4 0.562883291 0.731748278 1.102414287

1 138 37 4 0.3048317 0.643597034 0.454504136

1 139 37 4 0.306546737 0.57885815 0.454504136

1 140 37 4 0.371134435 0.762246103 0.454504136

1 141 37 4 0.380488499 0.738407726 0.454504136

1 142 37 4 0.381532421 0.745052582 0.454504136

1 144 37 4 0.751553801 1.203626632 0.404792746

1 145 37 4 0.858209478 1.226135519 0.404792746

1 146 37 4 0.716816304 1.246899674 0.404792746

1 147 37 4 0.751850301 1.192536574 0.404792746

1 150 37 4 0.958563189 1.365069018 0.404792746

1 151 37 4 0.952883208 1.374374635 0.404792746

1 152 37 4 0.997343624 1.480444814 0.404792746

2 156 39 4 0.391580316 0.616961947 1

2 162 44 4 0.245992675 0.325742997 1

2 163 45 4 0.309752355 0.498058472 1

2 61 16 5 0.298069837 0.522539437 0.758410584

2 62 16 5 0.359699621 0.549066586 0.58903222

2 63 16 5 0.242355842 0.585350974 1

2 64 16 5 0.296109559 0.579975198 1

2 73 19 5 0.252435807 0.401166961 0.353419332

2 74 19 5 0.268413487 0.42289428 1.17806444

2 75 19 5 0.232437418 0.365659284 0.58903222

2 76 19 5 0.265382551 0.396811245 1

2 82 22 5 0.371737591 0.74239924 0.88354833

2 84 22 5 0.387857067 0.719151645 0.411910643

2 110 30 5 0.376541101 0.705817628 0.519734312

2 111 30 5 0.313521669 0.573476823 0.519734312

2 157 40 5 0.13251226 0.248460488 2.1036865

2 158 40 5 0.171109581 0.375034699 2.1036865

1 188 52 5 0.180664989 0.396601877 1.497499176

1 189 52 5 0.257170236 0.556401798 1.497499176

2 208 59 5 0.134474123 0.52074463 0.930050874

2 209 59 5 0.137261084 0.499616121 0.930050874

2 210 59 5 0.169517996 0.556230925 0.930050874

2 211 59 5 0.202462683 0.671044438 0.930050874

2 213 60 5 0.203906213 0.595760762 1.963440734

2 214 60 5 0.190338046 0.571724354 1.963440734

2 215 60 5 0.185997928 0.553206422 1.963440734

2 216 60 5 0.238712801 0.59765068 1.963440734

2 217 60 5 0.159770799 0.572210412 1.963440734

;

proc means

Obs Study FOLDS PfecalurinaryBW075 PintakeBW075 InvTruSEMPfecalPolMis;

run;

proc means

Obs Study FOLDS PfecalurinaryBW075 PintakeBW075 InvTruSEMPfecalPolMis;

by FOLDS;

run;

PROC nLmixed ;

Title CrosvalidationRun1#;

parms a = 0.13 b = 1.40

ve= 0.0006 va= -35;

model PfecalurinaryBW075 ~ normal (a*exp(b*PintakeBW075)+ u1,ve);

random u1 ~ normal(0, va)Subject=Study;

replicate InvTruSEMPfecalPolMis;

run;

data KCROSSVALIDATIONRUN2;

input Model Obs Study FOLDS PfecalurinaryBW075 PintakeBW075 InvTruSEMPfecalPolMis;

cards;

1 9 3 1 0.505768414 0.664019852 0.647668394

1 10 3 1 0.526444481 0.70855292 0.647668394

1 11 3 1 0.49543038 0.699805353 0.647668394

1 12 3 1 0.500201781 0.672767419 0.647668394

2 65 17 1 0.28182751 0.455013771 1.218687352

2 66 17 1 0.352041778 0.55384749 2.1036865

2 67 17 1 0.345864026 0.504737903 0.494985059

2 68 17 1 0.461937682 0.583028142 0.292082093

2 86 23 1 0.347295495 0.581875487 0.409999225

2 87 23 1 0.325131564 0.60335538 0.981720367

2 88 23 1 0.316383018 0.570016339 0.690272133

2 89 23 1 0.328112506 0.616712685 0.435245483

2 96 25 1 0.378097072 0.512131484 0.31387152

2 97 25 1 0.357297776 0.559986203 0.209620007

2 120 33 1 0.331390337 0.548494268 1.244434268

2 121 33 1 0.326440609 0.538836261 1.244434268

2 122 33 1 0.331953721 0.536824177 1.244434268

2 123 33 1 0.349981999 0.573846534 1.244434268

2 124 33 1 0.34382502 0.564188528 1.244434268

2 125 33 1 0.349338132 0.562176443 1.244434268

2 161 43 1 0.419510514 0.521543323 1

1 176 47 1 0.122285401 0.231053393 2.233339289

1 177 47 1 0.16539853 0.360309879 2.233339289

1 178 47 1 0.217035321 0.535808516 2.233339289

1 228 64 1 0.167234386 0.296144225 2.158894646

1 229 64 1 0.157653249 0.296144225 2.158894646

2 57 15 3 0.291686538 0.41179276 1.328644105

2 58 15 3 0.296783473 0.45222145 0.441774165

2 59 15 3 0.352050655 0.45636196 0.50488476

2 60 15 3 0.344794632 0.548240835 1.17806444

2 79 21 3 0.276102418 0.444549729 1.06451606

2 80 21 3 0.269912312 0.357353212 0.654480245

2 81 21 3 0.23738749 0.371046967 0.4207373

2 92 24 3 0.32339724 0.428136914 0.312760471

2 93 24 3 0.446229171 0.699946812 0.302067805

2 94 24 3 0.38652876 0.534637911 0.218160082

2 95 24 3 0.272021518 0.37221265 0.191037477

2 102 27 3 0.134062215 0.265497998 1.125539274

2 103 27 3 0.177870713 0.276738225 1.6829492

2 115 32 3 0.417935842 0.57408307 1.425077952

2 117 32 3 0.412885258 0.572399542 1.425077952

2 118 32 3 0.341251139 0.451185521 1.425077952

1 130 35 3 0.402204225 0.788886573 0.835701153

1 131 35 3 0.360422634 0.631442188 0.835701153

1 132 35 3 0.402399342 0.701445955 0.835701153

1 133 35 3 0.345280454 0.627862029 0.835701153

1 134 36 3 0.703397669 0.998058521 0.681756204

1 135 36 3 0.701069749 0.994029249 0.681756204

1 190 53 3 0.417782565 0.78446942 0.785052599

1 191 53 3 0.585697092 0.843580143 0.785052599

1 192 53 3 0.650318136 0.989854134 0.785052599

1 193 53 3 0.512059158 0.934750918 0.785052599

2 26 8 4 0.134706395 0.419169681 0.694181129

2 27 8 4 0.134676005 0.417042523 0.694181129

2 69 18 4 0.380320122 0.567368806 0.690272133

2 70 18 4 0.332677954 0.549931982 0.591002227

2 71 18 4 0.288209535 0.458686021 0.550497402

2 72 18 4 0.331362822 0.51968255 0.706838664

2 90 24 4 0.31780181 0.599810242 0.821905423

1 106 29 4 0.547774318 0.735698336 1.102414287

1 107 29 4 0.523580212 0.757423656 1.102414287

1 108 29 4 0.596360034 0.768286316 1.102414287

1 109 29 4 0.562883291 0.731748278 1.102414287

1 138 37 4 0.3048317 0.643597034 0.454504136

1 139 37 4 0.306546737 0.57885815 0.454504136

1 140 37 4 0.371134435 0.762246103 0.454504136

1 141 37 4 0.380488499 0.738407726 0.454504136

1 142 37 4 0.381532421 0.745052582 0.454504136

1 144 37 4 0.751553801 1.203626632 0.404792746

1 145 37 4 0.858209478 1.226135519 0.404792746

1 146 37 4 0.716816304 1.246899674 0.404792746

1 147 37 4 0.751850301 1.192536574 0.404792746

1 150 37 4 0.958563189 1.365069018 0.404792746

1 151 37 4 0.952883208 1.374374635 0.404792746

1 152 37 4 0.997343624 1.480444814 0.404792746

2 156 39 4 0.391580316 0.616961947 1

2 162 44 4 0.245992675 0.325742997 1

2 163 45 4 0.309752355 0.498058472 1

2 61 16 5 0.298069837 0.522539437 0.758410584

2 62 16 5 0.359699621 0.549066586 0.58903222

2 63 16 5 0.242355842 0.585350974 1

2 64 16 5 0.296109559 0.579975198 1

2 73 19 5 0.252435807 0.401166961 0.353419332

2 74 19 5 0.268413487 0.42289428 1.17806444

2 75 19 5 0.232437418 0.365659284 0.58903222

2 76 19 5 0.265382551 0.396811245 1

2 82 22 5 0.371737591 0.74239924 0.88354833

2 84 22 5 0.387857067 0.719151645 0.411910643

2 110 30 5 0.376541101 0.705817628 0.519734312

2 111 30 5 0.313521669 0.573476823 0.519734312

2 157 40 5 0.13251226 0.248460488 2.1036865

2 158 40 5 0.171109581 0.375034699 2.1036865

1 188 52 5 0.180664989 0.396601877 1.497499176

1 189 52 5 0.257170236 0.556401798 1.497499176

2 208 59 5 0.134474123 0.52074463 0.930050874

2 209 59 5 0.137261084 0.499616121 0.930050874

2 210 59 5 0.169517996 0.556230925 0.930050874

2 211 59 5 0.202462683 0.671044438 0.930050874

2 213 60 5 0.203906213 0.595760762 1.963440734

2 214 60 5 0.190338046 0.571724354 1.963440734

2 215 60 5 0.185997928 0.553206422 1.963440734

2 216 60 5 0.238712801 0.59765068 1.963440734

2 217 60 5 0.159770799 0.572210412 1.963440734

;

proc means

Obs Study FOLDS PfecalurinaryBW075 PintakeBW075 InvTruSEMPfecalPolMis;

run;

proc means

Obs Study FOLDS PfecalurinaryBW075 PintakeBW075 InvTruSEMPfecalPolMis;

by FOLDS;

run;

PROC nLmixed;

Title CrosvalidationRun2#;

parms a = 0.13 b = 1.40

ve= 0.0006 va= -35;

model PfecalurinaryBW075 ~ normal (a*exp(b*PintakeBW075)+ u1,ve);

random u1 ~ normal(0, va)Subject=Study;

replicate InvTruSEMPfecalPolMis;

run;

data KCROSSVALIDATIONRUN3;

input Model Obs Study FOLDS PfecalurinaryBW075 PintakeBW075 InvTruSEMPfecalPolMis;

cards;

1 9 3 1 0.505768414 0.664019852 0.647668394

1 10 3 1 0.526444481 0.70855292 0.647668394

1 11 3 1 0.49543038 0.699805353 0.647668394

1 12 3 1 0.500201781 0.672767419 0.647668394

2 65 17 1 0.28182751 0.455013771 1.218687352

2 66 17 1 0.352041778 0.55384749 2.1036865

2 67 17 1 0.345864026 0.504737903 0.494985059

2 68 17 1 0.461937682 0.583028142 0.292082093

2 86 23 1 0.347295495 0.581875487 0.409999225

2 87 23 1 0.325131564 0.60335538 0.981720367

2 88 23 1 0.316383018 0.570016339 0.690272133

2 89 23 1 0.328112506 0.616712685 0.435245483

2 96 25 1 0.378097072 0.512131484 0.31387152

2 97 25 1 0.357297776 0.559986203 0.209620007

2 120 33 1 0.331390337 0.548494268 1.244434268

2 121 33 1 0.326440609 0.538836261 1.244434268

2 122 33 1 0.331953721 0.536824177 1.244434268

2 123 33 1 0.349981999 0.573846534 1.244434268

2 124 33 1 0.34382502 0.564188528 1.244434268

2 125 33 1 0.349338132 0.562176443 1.244434268

2 161 43 1 0.419510514 0.521543323 1

1 176 47 1 0.122285401 0.231053393 2.233339289

1 177 47 1 0.16539853 0.360309879 2.233339289

1 178 47 1 0.217035321 0.535808516 2.233339289

1 228 64 1 0.167234386 0.296144225 2.158894646

1 229 64 1 0.157653249 0.296144225 2.158894646

1 2 1 2 0.207080663 0.32748785 1.750455118

1 3 1 2 0.218325233 0.283986957 1.750455118

1 5 1 2 0.187464223 0.251976867 1.799078872

1 6 1 2 0.229651881 0.337337108 1.799078872

2 78 20 2 0.276102418 0.444549729 1.06451606

2 98 26 2 0.23320876 0.501710241 0.88354833

2 99 26 2 0.163625592 0.546374174 1.318728851

2 100 26 2 0.182263161 0.511198295 0.48018931

2 101 26 2 0.193042881 0.482378207 1.436663951

1 112 31 2 0.293378764 0.547052132 0.925240563

1 113 31 2 0.299520717 0.55830058 1.03626943

2 159 41 2 0.345129028 0.485046645 1

2 160 42 2 0.474888549 0.653767017 1

2 164 46 2 0.453253097 0.501201361 1

2 165 46 2 0.277832621 0.501201361 1

2 166 46 2 0.285517709 0.501201361 1

2 167 46 2 0.352511624 0.501201361 1

2 168 46 2 0.376485756 0.684975193 1

2 169 46 2 0.35827544 0.684975193 1

2 170 46 2 0.391271196 0.684975193 1

2 171 46 2 0.422930415 0.684975193 1

2 172 46 2 0.431200238 0.935575874 1

2 173 46 2 0.521082348 0.935575874 1

2 174 46 2 0.595677818 0.935575874 1

2 175 46 2 0.667349612 0.935575874 1

2 206 58 2 0.169289194 0.304063115 2.1036865

2 207 58 2 0.112605126 0.178015456 2.1036865

2 26 8 4 0.134706395 0.419169681 0.694181129

2 27 8 4 0.134676005 0.417042523 0.694181129

2 69 18 4 0.380320122 0.567368806 0.690272133

2 70 18 4 0.332677954 0.549931982 0.591002227

2 71 18 4 0.288209535 0.458686021 0.550497402

2 72 18 4 0.331362822 0.51968255 0.706838664

2 90 24 4 0.31780181 0.599810242 0.821905423

1 106 29 4 0.547774318 0.735698336 1.102414287

1 107 29 4 0.523580212 0.757423656 1.102414287

1 108 29 4 0.596360034 0.768286316 1.102414287

1 109 29 4 0.562883291 0.731748278 1.102414287

1 138 37 4 0.3048317 0.643597034 0.454504136

1 139 37 4 0.306546737 0.57885815 0.454504136

1 140 37 4 0.371134435 0.762246103 0.454504136

1 141 37 4 0.380488499 0.738407726 0.454504136

1 142 37 4 0.381532421 0.745052582 0.454504136

1 144 37 4 0.751553801 1.203626632 0.404792746

1 145 37 4 0.858209478 1.226135519 0.404792746

1 146 37 4 0.716816304 1.246899674 0.404792746

1 147 37 4 0.751850301 1.192536574 0.404792746

1 150 37 4 0.958563189 1.365069018 0.404792746

1 151 37 4 0.952883208 1.374374635 0.404792746

1 152 37 4 0.997343624 1.480444814 0.404792746

2 156 39 4 0.391580316 0.616961947 1

2 162 44 4 0.245992675 0.325742997 1

2 163 45 4 0.309752355 0.498058472 1

2 61 16 5 0.298069837 0.522539437 0.758410584

2 62 16 5 0.359699621 0.549066586 0.58903222

2 63 16 5 0.242355842 0.585350974 1

2 64 16 5 0.296109559 0.579975198 1

2 73 19 5 0.252435807 0.401166961 0.353419332

2 74 19 5 0.268413487 0.42289428 1.17806444

2 75 19 5 0.232437418 0.365659284 0.58903222

2 76 19 5 0.265382551 0.396811245 1

2 82 22 5 0.371737591 0.74239924 0.88354833

2 84 22 5 0.387857067 0.719151645 0.411910643

2 110 30 5 0.376541101 0.705817628 0.519734312

2 111 30 5 0.313521669 0.573476823 0.519734312

2 157 40 5 0.13251226 0.248460488 2.1036865

2 158 40 5 0.171109581 0.375034699 2.1036865

1 188 52 5 0.180664989 0.396601877 1.497499176

1 189 52 5 0.257170236 0.556401798 1.497499176

2 208 59 5 0.134474123 0.52074463 0.930050874

2 209 59 5 0.137261084 0.499616121 0.930050874

2 210 59 5 0.169517996 0.556230925 0.930050874

2 211 59 5 0.202462683 0.671044438 0.930050874

2 213 60 5 0.203906213 0.595760762 1.963440734

2 214 60 5 0.190338046 0.571724354 1.963440734

2 215 60 5 0.185997928 0.553206422 1.963440734

2 216 60 5 0.238712801 0.59765068 1.963440734

2 217 60 5 0.159770799 0.572210412 1.963440734

;

proc means

Obs Study FOLDS PfecalurinaryBW075 PintakeBW075 InvTruSEMPfecalPolMis;

run;

proc means

Obs Study FOLDS PfecalurinaryBW075 PintakeBW075 InvTruSEMPfecalPolMis;

by FOLDS;

run;

PROC nLmixed;

Title CrosvalidationRun3#;

parms a = 0.13 b = 1.40

ve= 0.0008 va= -35;

model PfecalurinaryBW075 ~ normal (a*exp(b*PintakeBW075)+ u1,ve);

random u1 ~ normal(0, va)Subject=Study;

replicate InvTruSEMPfecalPolMis;

run;

data KCROSSVALIDATIONRUN4;

input Model Obs Study FOLDS PfecalurinaryBW075 PintakeBW075 InvTruSEMPfecalPolMis;

cards;

1 9 3 1 0.505768414 0.664019852 0.647668394

1 10 3 1 0.526444481 0.70855292 0.647668394

1 11 3 1 0.49543038 0.699805353 0.647668394

1 12 3 1 0.500201781 0.672767419 0.647668394

2 65 17 1 0.28182751 0.455013771 1.218687352

2 66 17 1 0.352041778 0.55384749 2.1036865

2 67 17 1 0.345864026 0.504737903 0.494985059

2 68 17 1 0.461937682 0.583028142 0.292082093

2 86 23 1 0.347295495 0.581875487 0.409999225

2 87 23 1 0.325131564 0.60335538 0.981720367

2 88 23 1 0.316383018 0.570016339 0.690272133

2 89 23 1 0.328112506 0.616712685 0.435245483

2 96 25 1 0.378097072 0.512131484 0.31387152

2 97 25 1 0.357297776 0.559986203 0.209620007

2 120 33 1 0.331390337 0.548494268 1.244434268

2 121 33 1 0.326440609 0.538836261 1.244434268

2 122 33 1 0.331953721 0.536824177 1.244434268

2 123 33 1 0.349981999 0.573846534 1.244434268

2 124 33 1 0.34382502 0.564188528 1.244434268

2 125 33 1 0.349338132 0.562176443 1.244434268

2 161 43 1 0.419510514 0.521543323 1

1 176 47 1 0.122285401 0.231053393 2.233339289

1 177 47 1 0.16539853 0.360309879 2.233339289

1 178 47 1 0.217035321 0.535808516 2.233339289

1 228 64 1 0.167234386 0.296144225 2.158894646

1 229 64 1 0.157653249 0.296144225 2.158894646

1 2 1 2 0.207080663 0.32748785 1.750455118

1 3 1 2 0.218325233 0.283986957 1.750455118

1 5 1 2 0.187464223 0.251976867 1.799078872

1 6 1 2 0.229651881 0.337337108 1.799078872

2 78 20 2 0.276102418 0.444549729 1.06451606

2 98 26 2 0.23320876 0.501710241 0.88354833

2 99 26 2 0.163625592 0.546374174 1.318728851

2 100 26 2 0.182263161 0.511198295 0.48018931

2 101 26 2 0.193042881 0.482378207 1.436663951

1 112 31 2 0.293378764 0.547052132 0.925240563

1 113 31 2 0.299520717 0.55830058 1.03626943

2 159 41 2 0.345129028 0.485046645 1

2 160 42 2 0.474888549 0.653767017 1

2 164 46 2 0.453253097 0.501201361 1

2 165 46 2 0.277832621 0.501201361 1

2 166 46 2 0.285517709 0.501201361 1

2 167 46 2 0.352511624 0.501201361 1

2 168 46 2 0.376485756 0.684975193 1

2 169 46 2 0.35827544 0.684975193 1

2 170 46 2 0.391271196 0.684975193 1

2 171 46 2 0.422930415 0.684975193 1

2 172 46 2 0.431200238 0.935575874 1

2 173 46 2 0.521082348 0.935575874 1

2 174 46 2 0.595677818 0.935575874 1

2 175 46 2 0.667349612 0.935575874 1

2 206 58 2 0.169289194 0.304063115 2.1036865

2 207 58 2 0.112605126 0.178015456 2.1036865

2 57 15 3 0.291686538 0.41179276 1.328644105

2 58 15 3 0.296783473 0.45222145 0.441774165

2 59 15 3 0.352050655 0.45636196 0.50488476

2 60 15 3 0.344794632 0.548240835 1.17806444

2 79 21 3 0.276102418 0.444549729 1.06451606

2 80 21 3 0.269912312 0.357353212 0.654480245

2 81 21 3 0.23738749 0.371046967 0.4207373

2 92 24 3 0.32339724 0.428136914 0.312760471

2 93 24 3 0.446229171 0.699946812 0.302067805

2 94 24 3 0.38652876 0.534637911 0.218160082

2 95 24 3 0.272021518 0.37221265 0.191037477

2 102 27 3 0.134062215 0.265497998 1.125539274

2 103 27 3 0.177870713 0.276738225 1.6829492

2 115 32 3 0.417935842 0.57408307 1.425077952

2 117 32 3 0.412885258 0.572399542 1.425077952

2 118 32 3 0.341251139 0.451185521 1.425077952

1 130 35 3 0.402204225 0.788886573 0.835701153

1 131 35 3 0.360422634 0.631442188 0.835701153

1 132 35 3 0.402399342 0.701445955 0.835701153

1 133 35 3 0.345280454 0.627862029 0.835701153

1 134 36 3 0.703397669 0.998058521 0.681756204

1 135 36 3 0.701069749 0.994029249 0.681756204

1 190 53 3 0.417782565 0.78446942 0.785052599

1 191 53 3 0.585697092 0.843580143 0.785052599

1 192 53 3 0.650318136 0.989854134 0.785052599

1 193 53 3 0.512059158 0.934750918 0.785052599

2 61 16 5 0.298069837 0.522539437 0.758410584

2 62 16 5 0.359699621 0.549066586 0.58903222

2 63 16 5 0.242355842 0.585350974 1

2 64 16 5 0.296109559 0.579975198 1

2 73 19 5 0.252435807 0.401166961 0.353419332

2 74 19 5 0.268413487 0.42289428 1.17806444

2 75 19 5 0.232437418 0.365659284 0.58903222

2 76 19 5 0.265382551 0.396811245 1

2 82 22 5 0.371737591 0.74239924 0.88354833

2 84 22 5 0.387857067 0.719151645 0.411910643

2 110 30 5 0.376541101 0.705817628 0.519734312

2 111 30 5 0.313521669 0.573476823 0.519734312

2 157 40 5 0.13251226 0.248460488 2.1036865

2 158 40 5 0.171109581 0.375034699 2.1036865

1 188 52 5 0.180664989 0.396601877 1.497499176

1 189 52 5 0.257170236 0.556401798 1.497499176

2 208 59 5 0.134474123 0.52074463 0.930050874

2 209 59 5 0.137261084 0.499616121 0.930050874

2 210 59 5 0.169517996 0.556230925 0.930050874

2 211 59 5 0.202462683 0.671044438 0.930050874

2 213 60 5 0.203906213 0.595760762 1.963440734

2 214 60 5 0.190338046 0.571724354 1.963440734

2 215 60 5 0.185997928 0.553206422 1.963440734

2 216 60 5 0.238712801 0.59765068 1.963440734

2 217 60 5 0.159770799 0.572210412 1.963440734

;

proc means

Obs Study FOLDS PfecalurinaryBW075 PintakeBW075 InvTruSEMPfecalPolMis;

run;

proc means

Obs Study FOLDS PfecalurinaryBW075 PintakeBW075 InvTruSEMPfecalPolMis;

by FOLDS;

run;

PROC nLmixed;

Title CrosvalidationRun4#;

parms a = 0.13 b = 1.40

ve= 0.0006 va= -35;

model PfecalurinaryBW075 ~ normal (a*exp(b*PintakeBW075)+ u1,ve);

random u1 ~ normal(0, va)Subject=Study;

replicate InvTruSEMPfecalPolMis;

run;

data KCROSSVALIDATIONRUN5;

input Model Obs Study FOLDS PfecalurinaryBW075 PintakeBW075 InvTruSEMPfecalPolMis;

cards;

1 9 3 1 0.505768414 0.664019852 0.647668394

1 10 3 1 0.526444481 0.70855292 0.647668394

1 11 3 1 0.49543038 0.699805353 0.647668394

1 12 3 1 0.500201781 0.672767419 0.647668394

2 65 17 1 0.28182751 0.455013771 1.218687352

2 66 17 1 0.352041778 0.55384749 2.1036865

2 67 17 1 0.345864026 0.504737903 0.494985059

2 68 17 1 0.461937682 0.583028142 0.292082093

2 86 23 1 0.347295495 0.581875487 0.409999225

2 87 23 1 0.325131564 0.60335538 0.981720367

2 88 23 1 0.316383018 0.570016339 0.690272133

2 89 23 1 0.328112506 0.616712685 0.435245483

2 96 25 1 0.378097072 0.512131484 0.31387152

2 97 25 1 0.357297776 0.559986203 0.209620007

2 120 33 1 0.331390337 0.548494268 1.244434268

2 121 33 1 0.326440609 0.538836261 1.244434268

2 122 33 1 0.331953721 0.536824177 1.244434268

2 123 33 1 0.349981999 0.573846534 1.244434268

2 124 33 1 0.34382502 0.564188528 1.244434268

2 125 33 1 0.349338132 0.562176443 1.244434268

2 161 43 1 0.419510514 0.521543323 1

1 176 47 1 0.122285401 0.231053393 2.233339289

1 177 47 1 0.16539853 0.360309879 2.233339289

1 178 47 1 0.217035321 0.535808516 2.233339289

1 228 64 1 0.167234386 0.296144225 2.158894646

1 229 64 1 0.157653249 0.296144225 2.158894646

1 2 1 2 0.207080663 0.32748785 1.750455118

1 3 1 2 0.218325233 0.283986957 1.750455118

1 5 1 2 0.187464223 0.251976867 1.799078872

1 6 1 2 0.229651881 0.337337108 1.799078872

2 78 20 2 0.276102418 0.444549729 1.06451606

2 98 26 2 0.23320876 0.501710241 0.88354833

2 99 26 2 0.163625592 0.546374174 1.318728851

2 100 26 2 0.182263161 0.511198295 0.48018931

2 101 26 2 0.193042881 0.482378207 1.436663951

1 112 31 2 0.293378764 0.547052132 0.925240563

1 113 31 2 0.299520717 0.55830058 1.03626943

2 159 41 2 0.345129028 0.485046645 1

2 160 42 2 0.474888549 0.653767017 1

2 164 46 2 0.453253097 0.501201361 1

2 165 46 2 0.277832621 0.501201361 1

2 166 46 2 0.285517709 0.501201361 1

2 167 46 2 0.352511624 0.501201361 1

2 168 46 2 0.376485756 0.684975193 1

2 169 46 2 0.35827544 0.684975193 1

2 170 46 2 0.391271196 0.684975193 1

2 171 46 2 0.422930415 0.684975193 1

2 172 46 2 0.431200238 0.935575874 1

2 173 46 2 0.521082348 0.935575874 1

2 174 46 2 0.595677818 0.935575874 1

2 175 46 2 0.667349612 0.935575874 1

2 206 58 2 0.169289194 0.304063115 2.1036865

2 207 58 2 0.112605126 0.178015456 2.1036865

2 57 15 3 0.291686538 0.41179276 1.328644105

2 58 15 3 0.296783473 0.45222145 0.441774165

2 59 15 3 0.352050655 0.45636196 0.50488476

2 60 15 3 0.344794632 0.548240835 1.17806444

2 79 21 3 0.276102418 0.444549729 1.06451606

2 80 21 3 0.269912312 0.357353212 0.654480245

2 81 21 3 0.23738749 0.371046967 0.4207373

2 92 24 3 0.32339724 0.428136914 0.312760471

2 93 24 3 0.446229171 0.699946812 0.302067805

2 94 24 3 0.38652876 0.534637911 0.218160082

2 95 24 3 0.272021518 0.37221265 0.191037477

2 102 27 3 0.134062215 0.265497998 1.125539274

2 103 27 3 0.177870713 0.276738225 1.6829492

2 115 32 3 0.417935842 0.57408307 1.425077952

2 117 32 3 0.412885258 0.572399542 1.425077952

2 118 32 3 0.341251139 0.451185521 1.425077952

1 130 35 3 0.402204225 0.788886573 0.835701153

1 131 35 3 0.360422634 0.631442188 0.835701153

1 132 35 3 0.402399342 0.701445955 0.835701153

1 133 35 3 0.345280454 0.627862029 0.835701153

1 134 36 3 0.703397669 0.998058521 0.681756204

1 135 36 3 0.701069749 0.994029249 0.681756204

1 190 53 3 0.417782565 0.78446942 0.785052599

1 191 53 3 0.585697092 0.843580143 0.785052599

1 192 53 3 0.650318136 0.989854134 0.785052599

1 193 53 3 0.512059158 0.934750918 0.785052599

2 26 8 4 0.134706395 0.419169681 0.694181129

2 27 8 4 0.134676005 0.417042523 0.694181129

2 69 18 4 0.380320122 0.567368806 0.690272133

2 70 18 4 0.332677954 0.549931982 0.591002227

2 71 18 4 0.288209535 0.458686021 0.550497402

2 72 18 4 0.331362822 0.51968255 0.706838664

2 90 24 4 0.31780181 0.599810242 0.821905423

1 106 29 4 0.547774318 0.735698336 1.102414287

1 107 29 4 0.523580212 0.757423656 1.102414287

1 108 29 4 0.596360034 0.768286316 1.102414287

1 109 29 4 0.562883291 0.731748278 1.102414287

1 138 37 4 0.3048317 0.643597034 0.454504136

1 139 37 4 0.306546737 0.57885815 0.454504136

1 140 37 4 0.371134435 0.762246103 0.454504136

1 141 37 4 0.380488499 0.738407726 0.454504136

1 142 37 4 0.381532421 0.745052582 0.454504136

1 144 37 4 0.751553801 1.203626632 0.404792746

1 145 37 4 0.858209478 1.226135519 0.404792746

1 146 37 4 0.716816304 1.246899674 0.404792746

1 147 37 4 0.751850301 1.192536574 0.404792746

1 150 37 4 0.958563189 1.365069018 0.404792746

1 151 37 4 0.952883208 1.374374635 0.404792746

1 152 37 4 0.997343624 1.480444814 0.404792746

2 156 39 4 0.391580316 0.616961947 1

2 162 44 4 0.245992675 0.325742997 1

2 163 45 4 0.309752355 0.498058472 1

;

proc means

Obs Study FOLDS PfecalurinaryBW075 PintakeBW075 InvTruSEMPfecalPolMis;

run;

proc means

Obs Study FOLDS PfecalurinaryBW075 PintakeBW075 InvTruSEMPfecalPolMis;

run;

PROC nLmixed;

Title CrosvalidationRun5#;

parms a = 0.13 b = 1.40

ve= 0.0006 va= -35;

model PfecalurinaryBW075 ~ normal (a*exp(b*PintakeBW075)+ u1,ve);

random u1 ~ normal(0, va)Subject=Study;

replicate InvTruSEMPfecalPolMis;

run;

**11:23 Monday, September 6, 2023**

**The MEANS Procedure**

**Variable N Mean Std Dev Minimum Maximum**

**ƒƒƒƒƒƒƒƒƒƒƒƒƒƒƒƒƒƒƒƒƒƒƒƒƒƒƒƒƒƒƒƒƒƒƒƒƒƒƒƒƒƒƒƒƒƒƒƒƒƒƒƒƒƒƒƒƒƒƒƒƒƒƒƒƒƒƒƒƒƒƒƒƒƒƒƒƒƒƒƒƒƒƒƒƒƒƒƒƒƒƒƒ**

**Study 130 32.5384615 15.6374396 1.0000000 64.0000000**

**FOLDS 130 2.9769231 1.4112801 1.0000000 5.0000000**

**PfecalurinaryBW075 130 0.3572482 0.1757189 0.1126051 0.9973436**

**PintakeBW075 130 0.6004932 0.2373601 0.1780155 1.4804448**

**InvTruSEMPfecalPolMis 130 1.0002423 0.5308371 0.1910375 2.2333393**

**ƒƒƒƒƒƒƒƒƒƒƒƒƒƒƒƒƒƒƒƒƒƒƒƒƒƒƒƒƒƒƒƒƒƒƒƒƒƒƒƒƒƒƒƒƒƒƒƒƒƒƒƒƒƒƒƒƒƒƒƒƒƒƒƒƒƒƒƒƒƒƒƒƒƒƒƒƒƒƒƒƒƒƒƒƒƒƒƒƒƒƒƒ**

**CrosvalidationRun5# 11:23 Monday, September 6, 2023 127**

**---------------------------------------------- FOLDS=1 -----------------------------------------------**

**The MEANS Procedure**

**Variable N Mean Std Dev Minimum Maximum**

**ƒƒƒƒƒƒƒƒƒƒƒƒƒƒƒƒƒƒƒƒƒƒƒƒƒƒƒƒƒƒƒƒƒƒƒƒƒƒƒƒƒƒƒƒƒƒƒƒƒƒƒƒƒƒƒƒƒƒƒƒƒƒƒƒƒƒƒƒƒƒƒƒƒƒƒƒƒƒƒƒƒƒƒƒƒƒƒƒƒƒƒƒ**

**Study 26 28.1538462 16.9131719 3.0000000 64.0000000**

**FOLDS 26 1.0000000 0 1.0000000 1.0000000**

**PfecalurinaryBW075 26 0.3386108 0.1088338 0.1222854 0.5264445**

**PintakeBW075 26 0.5327392 0.1198592 0.2310534 0.7085529**

**InvTruSEMPfecalPolMis 26 1.1240483 0.6807444 0.2096200 2.2333393**

**ƒƒƒƒƒƒƒƒƒƒƒƒƒƒƒƒƒƒƒƒƒƒƒƒƒƒƒƒƒƒƒƒƒƒƒƒƒƒƒƒƒƒƒƒƒƒƒƒƒƒƒƒƒƒƒƒƒƒƒƒƒƒƒƒƒƒƒƒƒƒƒƒƒƒƒƒƒƒƒƒƒƒƒƒƒƒƒƒƒƒƒƒ**

**---------------------------------------------- FOLDS=2 -----------------------------------------------**

**Variable N Mean Std Dev Minimum Maximum**

**ƒƒƒƒƒƒƒƒƒƒƒƒƒƒƒƒƒƒƒƒƒƒƒƒƒƒƒƒƒƒƒƒƒƒƒƒƒƒƒƒƒƒƒƒƒƒƒƒƒƒƒƒƒƒƒƒƒƒƒƒƒƒƒƒƒƒƒƒƒƒƒƒƒƒƒƒƒƒƒƒƒƒƒƒƒƒƒƒƒƒƒƒ**

**Study 27 34.8518519 17.3198501 1.0000000 58.0000000**

**FOLDS 27 2.0000000 0 2.0000000 2.0000000**

**PfecalurinaryBW075 27 0.3229246 0.1387617 0.1126051 0.6673496**

**PintakeBW075 27 0.5518613 0.2116567 0.1780155 0.9355759**

**InvTruSEMPfecalPolMis 27 1.2019110 0.4079039 0.4801893 2.1036865**

**ƒƒƒƒƒƒƒƒƒƒƒƒƒƒƒƒƒƒƒƒƒƒƒƒƒƒƒƒƒƒƒƒƒƒƒƒƒƒƒƒƒƒƒƒƒƒƒƒƒƒƒƒƒƒƒƒƒƒƒƒƒƒƒƒƒƒƒƒƒƒƒƒƒƒƒƒƒƒƒƒƒƒƒƒƒƒƒƒƒƒƒƒ**

**---------------------------------------------- FOLDS=3 -----------------------------------------------**

**Variable N Mean Std Dev Minimum Maximum**

**ƒƒƒƒƒƒƒƒƒƒƒƒƒƒƒƒƒƒƒƒƒƒƒƒƒƒƒƒƒƒƒƒƒƒƒƒƒƒƒƒƒƒƒƒƒƒƒƒƒƒƒƒƒƒƒƒƒƒƒƒƒƒƒƒƒƒƒƒƒƒƒƒƒƒƒƒƒƒƒƒƒƒƒƒƒƒƒƒƒƒƒƒ**

**Study 26 30.5000000 11.9974997 15.0000000 53.0000000**

**FOLDS 26 3.0000000 0 3.0000000 3.0000000**

**PfecalurinaryBW075 26 0.3869819 0.1453122 0.1340622 0.7033977**

**PintakeBW075 26 0.5965686 0.2254090 0.2654980 0.9980585**

**InvTruSEMPfecalPolMis 26 0.8287453 0.4068281 0.1910375 1.6829492**

**ƒƒƒƒƒƒƒƒƒƒƒƒƒƒƒƒƒƒƒƒƒƒƒƒƒƒƒƒƒƒƒƒƒƒƒƒƒƒƒƒƒƒƒƒƒƒƒƒƒƒƒƒƒƒƒƒƒƒƒƒƒƒƒƒƒƒƒƒƒƒƒƒƒƒƒƒƒƒƒƒƒƒƒƒƒƒƒƒƒƒƒƒ**

**---------------------------------------------- FOLDS=4 -----------------------------------------------**

**Variable N Mean Std Dev Minimum Maximum**

**ƒƒƒƒƒƒƒƒƒƒƒƒƒƒƒƒƒƒƒƒƒƒƒƒƒƒƒƒƒƒƒƒƒƒƒƒƒƒƒƒƒƒƒƒƒƒƒƒƒƒƒƒƒƒƒƒƒƒƒƒƒƒƒƒƒƒƒƒƒƒƒƒƒƒƒƒƒƒƒƒƒƒƒƒƒƒƒƒƒƒƒƒ**

**Study 26 30.7692308 10.3123526 8.0000000 45.0000000**

**FOLDS 26 4.0000000 0 4.0000000 4.0000000**

**PfecalurinaryBW075 26 0.4934397 0.2552131 0.1346760 0.9973436**

**PintakeBW075 26 0.7893408 0.3402273 0.3257430 1.4804448**

**InvTruSEMPfecalPolMis 26 0.6640233 0.2716447 0.4047927 1.1024143**

**ƒƒƒƒƒƒƒƒƒƒƒƒƒƒƒƒƒƒƒƒƒƒƒƒƒƒƒƒƒƒƒƒƒƒƒƒƒƒƒƒƒƒƒƒƒƒƒƒƒƒƒƒƒƒƒƒƒƒƒƒƒƒƒƒƒƒƒƒƒƒƒƒƒƒƒƒƒƒƒƒƒƒƒƒƒƒƒƒƒƒƒƒ**

**CrosvalidationRun5# 11:23 Monday, September 6, 2023 128**

**---------------------------------------------- FOLDS=5 -----------------------------------------------**

**The MEANS Procedure**

**Variable N Mean Std Dev Minimum Maximum**

**ƒƒƒƒƒƒƒƒƒƒƒƒƒƒƒƒƒƒƒƒƒƒƒƒƒƒƒƒƒƒƒƒƒƒƒƒƒƒƒƒƒƒƒƒƒƒƒƒƒƒƒƒƒƒƒƒƒƒƒƒƒƒƒƒƒƒƒƒƒƒƒƒƒƒƒƒƒƒƒƒƒƒƒƒƒƒƒƒƒƒƒƒ**

**Study 25 38.5600000 18.9254678 16.0000000 60.0000000**

**FOLDS 25 5.0000000 0 5.0000000 5.0000000**

**PfecalurinaryBW075 25 0.2411384 0.0782775 0.1325123 0.3878571**

**PintakeBW075 25 0.5311599 0.1205510 0.2484605 0.7423992**

**InvTruSEMPfecalPolMis 25 1.1817066 0.5927737 0.3534193 2.1036865**

**ƒƒƒƒƒƒƒƒƒƒƒƒƒƒƒƒƒƒƒƒƒƒƒƒƒƒƒƒƒƒƒƒƒƒƒƒƒƒƒƒƒƒƒƒƒƒƒƒƒƒƒƒƒƒƒƒƒƒƒƒƒƒƒƒƒƒƒƒƒƒƒƒƒƒƒƒƒƒƒƒƒƒƒƒƒƒƒƒƒƒƒƒ**

**FINALMODEL_PExcretion 11:23 Monday, September 6, 2023 129**

**The NLMIXED Procedure**

**Specifications**

**Data Set WORK.COMPLETE_**

**FINALMODEL**

**Dependent Variable PfecalurinaryBW075**

**Distribution for Dependent Variable Normal**

**Random Effects u1**

**Distribution for Random Effects Normal**

**Subject Variable Study**

**Replicate Variable InvTruSEMPfecal**

**PolMis**

**Optimization Technique Dual Quasi-Newton**

**Integration Method Adaptive Gaussian**

**Quadrature**

**Dimensions**

**Observations Used 130**

**Observations Not Used 0**

**Total Observations 130**

**Subjects 26**

**Max Obs Per Subject 12**

**Parameters 4**

**Quadrature Points 1**

**Parameters**

**a b ve va NegLogLike**

**0.13 1.4 0.0006 -35 -268.32711**

**Iteration History**

**Iter Calls NegLogLike Diff MaxGrad Slope**

**1 67 -273.30105 4.973942 71609.03 -1.404E8**

**2 114 -273.36037 0.059315 74906.49 -4.861E7**

**3* 215 -273.36103 0.00066 74528.42 -5.611E7**

**4 260 -273.36134 0.000308 74714.83 -5.587E7**

**5* 305 -273.36135 0.000014 74760.96 -5.587E7**

**6* 350 -273.36135 4.147E-8 74763.83 -5.587E7**

**7* 449 -273.79586 0.434514 73446.1 -5.59E7**

**8 503 -273.81519 0.019327 75564.18 -1.144E8**

**9* 610 -273.81558 0.000385 75380.21 -5.71E7**

**10 710 -273.81562 0.000043 75258.62 -5.682E7**

**11 753 -273.81562 5.012E-6 75282.12 -5.669E7**

**12* 796 -273.81562 2.642E-7 75287.98 -5.669E7**

**13* 839 -273.81562 5.351E-9 75288.72 -5.669E7**

**FINALMODEL_PExcretion 11:23 Monday, September 6, 2023 130**

**The NLMIXED Procedure**

**Iteration History**

**Iter Calls NegLogLike Diff MaxGrad Slope**

**14* 932 -273.81565 0.000025 75334.84 -5.668E7**

**15* 987 -273.81566 0.000011 75273.81 -5.675E7**

**16 1030 -273.81566 1.26E-6 75285.55 -5.677E7**

**17* 1073 -273.81566 6.807E-8 75288.48 -5.677E7**

**18* 1116 -273.81566 1.652E-9 75289.22 -5.677E7**

**19* 1209 -273.8157 0.000043 75230.49 -5.668E7**

**20 1262 -273.81572 0.000019 75282.1 -8.514E7**

**21* 1314 -273.81572 2.792E-7 75288.5 -8.518E7**

**22* 1366 -273.81572 1.928E-9 75289.3 -8.519E7**

**23* 1423 -273.81572 3.4E-10 75289.3 -8.519E7**

**24* 1480 -273.81572 2.84E-13 75289.3 -8.519E7**

**NOTE: FCONV convergence criterion satisfied.**

**Fit Statistics**

**-2 Log Likelihood -547.6**

**AIC (smaller is better) -539.6**

**AICC (smaller is better) -539.3**

**BIC (smaller is better) -534.6**

**Parameter Estimates**

**Standard**

**Parameter Estimate Error DF t Value Pr > |t| Alpha Lower Upper Gradient**

**a 0.1352 0.04272 25 3.16 0.0041 0.05 0.04718 0.2232 -4.18274**

**b 1.4010 0.1863 25 7.52 <.0001 0.05 1.0175 1.7846 1.364055**

**ve 0.000862 . 25 . . 0.05 . . -0.15498**

**va -37.7832 . 25 . . 0.05 . . 75289.3**

**Covariance Matrix of Parameter Estimates**

**Row Parameter a b ve va**

**1 a 0.001825 -0.00788 . .**

**2 b -0.00788 0.03469 . .**

**3 ve . . . .**

**4 va . . . .**

**FINALMODEL_PExcretion 11:23 Monday, September 6, 2023 131**

**The NLMIXED Procedure**

**Correlation Matrix of Parameter Estimates**

**Row Parameter a b ve va**

**1 a 1.0000 -0.9908 . .**

**2 b -0.9908 1.0000 . .**

**3 ve . . 1.0000 .**

**4 va . . . 1.0000**

**P Effect Pfecal x Study 11:23 Monday, September 6, 2023 132**

**The Mixed Procedure**

**Model Information**

**Data Set WORK.COMPLETE_FINALMODEL**

**Dependent Variable PfecalurinaryBW075**

**Weight Variable InvTruSEMPfecalPolMis**

**Covariance Structure Variance Components**

**Estimation Method REML**

**Residual Variance Method Profile**

**Fixed Effects SE Method Model-Based**

**Degrees of Freedom Method Containment**

**Class Level Information**

**Class Levels Values**

**Study 39 1 3 8 15 16 17 18 19 20 21 22**

**23 24 25 26 27 29 30 31 32 33**

**35 36 37 39 40 41 42 43 44 45**

**46 47 52 53 58 59 60 64**

**Dimensions**

**Covariance Parameters 2**

**Columns in X 41**

**Columns in Z 39**

**Subjects 1**

**Max Obs Per Subject 130**

**Observations Used 130**

**Observations Not Used 0**

**Total Observations 130**

**Iteration History**

**Iteration Evaluations -2 Res Log Like Criterion**

**0 1 -318.19392192**

**1 3 -326.08230498 0.00033071**

**2 1 -326.17404840 0.00001732**

**3 1 -326.17846183 0.00000006**

**4 1 -326.17847584 0.00000000**

**Convergence criteria met.**

**P Effect Pfecal x Study 11:23 Monday, September 6, 2023 133**

**The Mixed Procedure**

**Covariance Parameter**

**Estimates**

**Cov Parm Estimate**

**Study 0.007820**

**Residual 0.001337**

**Fit Statistics**

**-2 Res Log Likelihood -326.2**

**AIC (smaller is better) -322.2**

**AICC (smaller is better) -322.0**

**BIC (smaller is better) -318.9**

**Solution for Fixed Effects**

**Standard**

**Effect Study Estimate Error DF t Value Pr > |t|**

**Intercept 0.02389 0.03608 30 0.66 0.5130**

**PintakeBW075 0.4679 0.3279 60 1.43 0.1588**

**PintakeBW075*Study 1 0.05409 0.3895 60 0.14 0.8900**

**PintakeBW075*Study 3 0.2313 0.3392 60 0.68 0.4980**

**PintakeBW075*Study 8 -0.2029 0.3797 60 -0.53 0.5952**

**PintakeBW075*Study 15 0.09653 0.3531 60 0.27 0.7855**

**PintakeBW075*Study 16 -0.04445 0.3482 60 -0.13 0.8988**

**PintakeBW075*Study 17 0.1798 0.3491 60 0.51 0.6085**

**PintakeBW075*Study 18 0.1370 0.3518 60 0.39 0.6983**

**PintakeBW075*Study 19 0.1176 0.3779 60 0.31 0.7568**

**PintakeBW075*Study 20 0.09948 0.3745 60 0.27 0.7914**

**PintakeBW075*Study 21 0.08374 0.3743 60 0.22 0.8237**

**PintakeBW075*Study 22 0.01019 0.3382 60 0.03 0.9761**

**PintakeBW075*Study 23 0.03737 0.3463 60 0.11 0.9144**

**PintakeBW075*Study 24 0.03949 0.3437 60 0.11 0.9089**

**PintakeBW075*Study 25 0.1746 0.3635 60 0.48 0.6327**

**PintakeBW075*Study 26 -0.1987 0.3537 60 -0.56 0.5763**

**PintakeBW075*Study 27 0.05733 0.4516 60 0.13 0.8994**

**PintakeBW075*Study 29 0.2436 0.3355 60 0.73 0.4705**

**PintakeBW075*Study 30 0.03237 0.3438 60 0.09 0.9253**

**PintakeBW075*Study 31 0.02540 0.3517 60 0.07 0.9427**

**PintakeBW075*Study 32 0.2021 0.3458 60 0.58 0.5612**

**PintakeBW075*Study 33 0.1022 0.3485 60 0.29 0.7704**

**PintakeBW075*Study 35 0.01821 0.3367 60 0.05 0.9571**

**PintakeBW075*Study 36 0.2132 0.3300 60 0.65 0.5208**

**PintakeBW075*Study 37 0.3037 0.3277 60 0.93 0.3578**

**PintakeBW075*Study 39 0.1281 0.3475 60 0.37 0.7137**

**PintakeBW075*Study 40 -0.1116 0.3711 60 -0.30 0.7646**

**PintakeBW075*Study 41 0.1944 0.3659 60 0.53 0.5971**

**PintakeBW075*Study 42 0.2220 0.3443 60 0.64 0.5216**

**P Effect Pfecal x Study 11:23 Monday, September 6, 2023 134**

**The Mixed Procedure**

**Solution for Fixed Effects**

**Standard**

**Effect Study Estimate Error DF t Value Pr > |t|**

**PintakeBW075*Study 43 0.2907 0.3594 60 0.81 0.4217**

**PintakeBW075*Study 44 0.2140 0.4232 60 0.51 0.6150**

**PintakeBW075*Study 45 0.1061 0.3634 60 0.29 0.7713**

**PintakeBW075*Study 46 0.04295 0.3290 60 0.13 0.8966**

**PintakeBW075*Study 47 -0.1443 0.3376 60 -0.43 0.6707**

**PintakeBW075*Study 52 -0.03479 0.3490 60 -0.10 0.9209**

**PintakeBW075*Study 53 0.1459 0.3305 60 0.44 0.6605**

**PintakeBW075*Study 58 -0.00510 0.3849 60 -0.01 0.9895**

**PintakeBW075*Study 59 -0.1860 0.3431 60 -0.54 0.5898**

**PintakeBW075*Study 60 -0.1352 0.3454 60 -0.39 0.6967**

**PintakeBW075*Study 64 0 . . . .**

**Type 3 Tests of Fixed Effects**

**Num Den**

**Effect DF DF F Value Pr > F**

**PintakeBW075 1 60 63.46 <.0001**

**PintakeBW075*Study 38 60 1.26 0.2121**

**P Effect Pfecal x Study 11:23 Monday, September 6, 2023 135**

**The Mixed Procedure**

**Model Information**

**Data Set WORK.COMPLETE_FINALMODEL**

**Dependent Variable PfecalurinaryBW075**

**Weight Variable InvTruSEMPfecalPolMis**

**Covariance Structure Variance Components**

**Estimation Method REML**

**Residual Variance Method Profile**

**Fixed Effects SE Method Model-Based**

**Degrees of Freedom Method Containment**

**Dimensions**

**Covariance Parameters 2**

**Columns in X 3**

**Columns in Z 1**

**Subjects 1**

**Max Obs Per Subject 130**

**Observations Used 130**

**Observations Not Used 0**

**Total Observations 130**

**Iteration History**

**Iteration Evaluations -2 Res Log Like Criterion**

**0 1 -288.66152275**

**1 1 -288.66152275 0.00000000**

**Convergence criteria met.**

**Covariance Parameter**

**Estimates**

**Cov Parm Estimate**

**Study 0**

**Residual 0.004585**

**Fit Statistics**

**-2 Res Log Likelihood -288.7**

**AIC (smaller is better) -286.7**

**AICC (smaller is better) -286.6**

**BIC (smaller is better) -288.7**

**P Effect Pfecal x Study 11:23 Monday, September 6, 2023 136**

**The Mixed Procedure**

**Solution for Fixed Effects**

**Standard**

**Effect Estimate Error DF t Value Pr > |t|**

**Intercept -0.05135 0.01672 126 -3.07 0.0026**

**PintakeBW075 0.8261 0.04112 126 20.09 <.0001**

**PintakeBW075*Study -0.00437 0.000707 126 -6.18 <.0001**

**Solution for Random Effects**

**Std Err**

**Effect Estimate Pred DF t Value Pr > |t|**

**Study 0 . . . .**

**Type 3 Tests of Fixed Effects**

**Num Den**

**Effect DF DF F Value Pr > F**

**PintakeBW075 1 126 403.51 <.0001**

**PintakeBW075*Study 1 126 38.25 <.0001**

**Variance Study Pfecal 11:23 Monday, September 6, 2023 137**

**The Mixed Procedure**

**Model Information**

**Data Set WORK.COMPLETE_FINALMODEL**

**Dependent Variable PfecalurinaryBW075**

**Weight Variable InvTruSEMPfecalPolMis**

**Covariance Structure Variance Components**

**Estimation Method Type 3**

**Residual Variance Method Factor**

**Fixed Effects SE Method Model-Based**

**Degrees of Freedom Method Containment**

**Class Level Information**

**Class Levels Values**

**Study 39 1 3 8 15 16 17 18 19 20 21 22**

**23 24 25 26 27 29 30 31 32 33**

**35 36 37 39 40 41 42 43 44 45**

**46 47 52 53 58 59 60 64**

**Dimensions**

**Covariance Parameters 2**

**Columns in X 2**

**Columns in Z 39**

**Subjects 1**

**Max Obs Per Subject 130**

**Observations Used 130**

**Observations Not Used 0**

**Total Observations 130**

**Type 3 Analysis of Variance**

**Sum of**

**Source DF Squares Mean Square Expected Mean Square Error Term**

**PintakeBW075 1 0.449904 0.449904 Var(Residual) + Q(PintakeBW075) MS(Residual)**

**Study 38 0.599997 0.015789 Var(Residual) + 3.1837 Var(Study) MS(Residual)**

**Residual 90 0.157678 0.001752 Var(Residual) .**

**Type 3 Analysis of Variance**

**Error**

**Source DF F Value Pr > F**

**PintakeBW075 90 256.80 <.0001**

**Study 90 9.01 <.0001**

**Residual . . .**

**Variance Study Pfecal 11:23 Monday, September 6, 2023 138**

**The Mixed Procedure**

**Covariance Parameter**

**Estimates**

**Cov Parm Estimate**

**Study 0.004409**

**Residual 0.001752**

**Fit Statistics**

**-2 Res Log Likelihood -353.1**

**AIC (smaller is better) -349.1**

**AICC (smaller is better) -349.0**

**BIC (smaller is better) -345.7**

**Solution for Fixed Effects**

**Standard**

**Effect Estimate Error DF t Value Pr > |t|**

**Intercept -0.01938 0.02135 38 -0.91 0.3698**

**PintakeBW075 0.6304 0.03304 90 19.08 <.0001**

**Type 3 Tests of Fixed Effects**

**Num Den**

**Effect DF DF F Value Pr > F**

**PintakeBW075 1 90 364.02 <.0001**

**CrosvalidationRun1# 11:23 Monday, September 6, 2023 139**

**The NLMIXED Procedure**

**Specifications**

**Data Set WORK.**

**KCROSSVALIDATIONRUN1**

**Dependent Variable PfecalurinaryBW075**

**Distribution for Dependent Variable Normal**

**Random Effects u1**

**Distribution for Random Effects Normal**

**Subject Variable Study**

**Replicate Variable InvTruSEMPfecal**

**PolMis**

**Optimization Technique Dual Quasi-Newton**

**Integration Method Adaptive Gaussian**

**Quadrature**

**Dimensions**

**Observations Used 104**

**Observations Not Used 0**

**Total Observations 104**

**Subjects 19**

**Max Obs Per Subject 12**

**Parameters 4**

**Quadrature Points 1**

**Parameters**

**a b ve va NegLogLike**

**0.13 1.4 0.0006 -35 -200.69772**

**Iteration History**

**Iter Calls NegLogLike Diff MaxGrad Slope**

**1 19 -210.34241 9.644687 50428.38 -1.168E8**

**2 123 -210.34382 0.001413 49951.25 -2.543E7**

**3 168 -210.34404 0.00022 50055.95 -2.502E7**

**4* 213 -210.34407 0.000024 50107.56 -2.502E7**

**5* 313 -210.49883 0.154765 47628.65 -2.511E7**

**6 368 -210.57223 0.073399 49674.8 -2.745E7**

**7* 422 -210.57625 0.004017 50130.16 -2.773E7**

**8* 477 -210.57664 0.000393 50348.6 -2.776E7**

**9* 530 -210.57664 1.84E-10 50348.6 -2.778E7**

**10* 588 -210.57664 4.16E-11 50348.6 -2.778E7**

**CrosvalidationRun1# 11:23 Monday, September 6, 2023 140**

**The NLMIXED Procedure**

**NOTE: FCONV convergence criterion satisfied.**

**Fit Statistics**

**-2 Log Likelihood -421.2**

**AIC (smaller is better) -413.2**

**AICC (smaller is better) -412.7**

**BIC (smaller is better) -409.4**

**Parameter Estimates**

**Standard**

**Parameter Estimate Error DF t Value Pr > |t| Alpha Lower Upper Gradient**

**a 0.1321 0.03080 18 4.29 0.0004 0.05 0.06738 0.1968 -81.2611**

**b 1.4004 0.1378 18 10.16 <.0001 0.05 1.1110 1.6899 -16.3055**

**ve 0.001037 . 18 . . 0.05 . . -20.6621**

**va -35.7850 . 18 . . 0.05 . . 50348.6**

**Covariance Matrix of Parameter Estimates**

**Row Parameter a b ve va**

**1 a 0.000949 -0.00415 . .**

**2 b -0.00415 0.01898 . .**

**3 ve . . . .**

**4 va . . . .**

**Correlation Matrix of Parameter Estimates**

**Row Parameter a b ve va**

**1 a 1.0000 -0.9780 . .**

**2 b -0.9780 1.0000 . .**

**3 ve . . 1.0000 .**

**4 va . . . 1.0000**

**CrosvalidationRun2# 11:23 Monday, September 6, 2023 141**

**The NLMIXED Procedure**

**Specifications**

**Data Set WORK.**

**KCROSSVALIDATIONRUN2**

**Dependent Variable PfecalurinaryBW075**

**Distribution for Dependent Variable Normal**

**Random Effects u1**

**Distribution for Random Effects Normal**

**Subject Variable Study**

**Replicate Variable InvTruSEMPfecal**

**PolMis**

**Optimization Technique Dual Quasi-Newton**

**Integration Method Adaptive Gaussian**

**Quadrature**

**Dimensions**

**Observations Used 103**

**Observations Not Used 0**

**Total Observations 103**

**Subjects 19**

**Max Obs Per Subject 12**

**Parameters 4**

**Quadrature Points 1**

**Parameters**

**a b ve va NegLogLike**

**0.13 1.4 0.0006 -35 -224.4461**

**Iteration History**

**Iter Calls NegLogLike Diff MaxGrad Slope**

**1 47 -224.76192 0.315821 86401.37 -6.427E7**

**2* 95 -224.80676 0.04484 89923.84 -7.479E7**

**3* 145 -224.80771 0.00095 90352.08 -8.086E7**

**4* 200 -224.81029 0.002583 89301.65 -8.164E7**

**5 245 -224.81296 0.002671 90136.73 -8.129E7**

**6* 290 -224.813 0.000044 90237.67 -8.131E7**

**7* 335 -224.81301 1.568E-6 90262.72 -8.131E7**

**8* 433 -225.54915 0.736146 83784.61 -8.147E7**

**9 490 -225.75426 0.205105 92200.44 -1.117E8**

**10* 543 -225.75426 5.85E-11 92200.44 -1.165E8**

**11* 602 -225.75426 4.18E-11 92200.44 -1.165E8**

**CrosvalidationRun2# 11:23 Monday, September 6, 2023 142**

**The NLMIXED Procedure**

**NOTE: FCONV convergence criterion satisfied.**

**Fit Statistics**

**-2 Log Likelihood -451.5**

**AIC (smaller is better) -443.5**

**AICC (smaller is better) -443.1**

**BIC (smaller is better) -439.7**

**Parameter Estimates**

**Standard**

**Parameter Estimate Error DF t Value Pr > |t| Alpha Lower Upper Gradient**

**a 0.1345 0.02619 18 5.14 <.0001 0.05 0.07950 0.1895 -114.464**

**b 1.4009 0.1110 18 12.62 <.0001 0.05 1.1677 1.6341 -18.6041**

**ve 0.000518 . 18 . . 0.05 . . -143.662**

**va -36.4174 . 18 . . 0.05 . . 92200.44**

**Covariance Matrix of Parameter Estimates**

**Row Parameter a b ve va**

**1 a 0.000686 -0.00285 . .**

**2 b -0.00285 0.01232 . .**

**3 ve . . . .**

**4 va . . . .**

**Correlation Matrix of Parameter Estimates**

**Row Parameter a b ve va**

**1 a 1.0000 -0.9808 . .**

**2 b -0.9808 1.0000 . .**

**3 ve . . 1.0000 .**

**4 va . . . 1.0000**

**CrosvalidationRun3# 11:23 Monday, September 6, 2023 143**

**The NLMIXED Procedure**

**Specifications**

**Data Set WORK.**

**KCROSSVALIDATIONRUN3**

**Dependent Variable PfecalurinaryBW075**

**Distribution for Dependent Variable Normal**

**Random Effects u1**

**Distribution for Random Effects Normal**

**Subject Variable Study**

**Replicate Variable InvTruSEMPfecal**

**PolMis**

**Optimization Technique Dual Quasi-Newton**

**Integration Method Adaptive Gaussian**

**Quadrature**

**Dimensions**

**Observations Used 104**

**Observations Not Used 0**

**Total Observations 104**

**Subjects 22**

**Max Obs Per Subject 12**

**Parameters 4**

**Quadrature Points 1**

**Parameters**

**a b ve va NegLogLike**

**0.13 1.4 0.0008 -35 -224.93206**

**Iteration History**

**Iter Calls NegLogLike Diff MaxGrad Slope**

**1 59 -225.24136 0.309302 59575.23 -4.579E7**

**2 105 -225.24691 0.005551 60697.9 -3.525E7**

**3* 156 -225.24691 1.88E-12 60697.9 -3.538E7**

**NOTE: FCONV convergence criterion satisfied.**

**Fit Statistics**

**-2 Log Likelihood -450.5**

**AIC (smaller is better) -442.5**

**AICC (smaller is better) -442.1**

**BIC (smaller is better) -438.1**

**CrosvalidationRun3# 11:23 Monday, September 6, 2023 144**

**The NLMIXED Procedure**

**Parameter Estimates**

**Standard**

**Parameter Estimate Error DF t Value Pr > |t| Alpha Lower Upper Gradient**

**a 0.1300 0.02451 21 5.30 <.0001 0.05 0.07903 0.1810 -136.177**

**b 1.4000 0.1123 21 12.47 <.0001 0.05 1.1665 1.6335 -28.4717**

**ve 0.000886 . 21 . . 0.05 . . -230.083**

**va -35.0009 . 21 . . 0.05 . . 60697.9**

**Covariance Matrix of Parameter Estimates**

**Row Parameter a b ve va**

**1 a 0.000601 -0.00267 . .**

**2 b -0.00267 0.01261 . .**

**3 ve . . . .**

**4 va . . . .**

**Correlation Matrix of Parameter Estimates**

**Row Parameter a b ve va**

**1 a 1.0000 -0.9698 . .**

**2 b -0.9698 1.0000 . .**

**3 ve . . 1.0000 .**

**4 va . . . 1.0000**

**CrosvalidationRun4# 11:23 Monday, September 6, 2023 145**

**The NLMIXED Procedure**

**Specifications**

**Data Set WORK.**

**KCROSSVALIDATIONRUN4**

**Dependent Variable PfecalurinaryBW075**

**Distribution for Dependent Variable Normal**

**Random Effects u1**

**Distribution for Random Effects Normal**

**Subject Variable Study**

**Replicate Variable InvTruSEMPfecal**

**PolMis**

**Optimization Technique Dual Quasi-Newton**

**Integration Method Adaptive Gaussian**

**Quadrature**

**Dimensions**

**Observations Used 104**

**Observations Not Used 0**

**Total Observations 104**

**Subjects 22**

**Max Obs Per Subject 12**

**Parameters 4**

**Quadrature Points 1**

**Parameters**

**a b ve va NegLogLike**

**0.13 1.4 0.0006 -35 -232.56362**

**Iteration History**

**Iter Calls NegLogLike Diff MaxGrad Slope**

**1 68 -236.92492 4.361291 64835.41 -1.055E8**

**2 167 -236.92493 0.000017 64794.1 -4.204E7**

**3 211 -236.92493 1.531E-7 64800.83 -4.21E7**

**4* 305 -236.92561 0.000673 64557.43 -4.199E7**

**5 358 -236.926 0.000395 64787.09 -4.427E7**

**6* 410 -236.926 1.179E-6 64801 -4.429E7**

**7* 468 -236.926 1.33E-10 64801 -4.429E7**

**8* 521 -236.926 1.42E-13 64801 -4.429E7**

**NOTE: FCONV convergence criterion satisfied.**

**CrosvalidationRun4# 11:23 Monday, September 6, 2023 146**

**The NLMIXED Procedure**

**Fit Statistics**

**-2 Log Likelihood -473.9**

**AIC (smaller is better) -465.9**

**AICC (smaller is better) -465.4**

**BIC (smaller is better) -461.5**

**Parameter Estimates**

**Standard**

**Parameter Estimate Error DF t Value Pr > |t| Alpha Lower Upper Gradient**

**a 0.1300 0.05807 21 2.24 0.0361 0.05 0.009293 0.2508 -22.7439**

**b 1.4000 0.3171 21 4.41 0.0002 0.05 0.7405 2.0595 -4.45556**

**ve 0.000868 . 21 . . 0.05 . . -0.59879**

**va -35.1282 . 21 . . 0.05 . . 64801**

**Covariance Matrix of Parameter Estimates**

**Row Parameter a b ve va**

**1 a 0.003372 -0.01812 . .**

**2 b -0.01812 0.1006 . .**

**3 ve . . . .**

**4 va . . . .**

**Correlation Matrix of Parameter Estimates**

**Row Parameter a b ve va**

**1 a 1.0000 -0.9841 . .**

**2 b -0.9841 1.0000 . .**

**3 ve . . 1.0000 .**

**4 va . . . 1.0000**

**CrosvalidationRun5# 11:23 Monday, September 6, 2023 147**

**The NLMIXED Procedure**

**Specifications**

**Data Set WORK.**

**KCROSSVALIDATIONRUN5**

**Dependent Variable PfecalurinaryBW075**

**Distribution for Dependent Variable Normal**

**Random Effects u1**

**Distribution for Random Effects Normal**

**Subject Variable Study**

**Replicate Variable InvTruSEMPfecal**

**PolMis**

**Optimization Technique Dual Quasi-Newton**

**Integration Method Adaptive Gaussian**

**Quadrature**

**Dimensions**

**Observations Used 105**

**Observations Not Used 0**

**Total Observations 105**

**Subjects 22**

**Max Obs Per Subject 12**

**Parameters 4**

**Quadrature Points 1**

**Parameters**

**a b ve va NegLogLike**

**0.13 1.4 0.0006 -35 -195.136**

**Iteration History**

**Iter Calls NegLogLike Diff MaxGrad Slope**

**1 67 -202.4884 7.352398 49828.09 -9.703E7**

**2 112 -202.4897 0.001301 50191.33 -2.249E7**

**3* 210 -202.80692 0.317217 48118.54 -2.519E7**

**4 266 -202.86648 0.059562 50282.39 -3.386E7**

**5* 319 -202.86727 0.000791 50517.03 -3.447E7**

**6* 371 -202.8673 0.00003 50574.17 -3.453E7**

**7* 424 -202.8673 7.9E-10 50574.17 -3.454E7**

**8* 522 -202.87604 0.00874 50226.45 -2.558E7**

**9 576 -202.87718 0.001144 50481.61 -3.829E7**

**10* 629 -202.87728 0.000097 50606.75 -3.841E7**

**11* 687 -202.87728 4.19E-10 50606.75 -3.847E7**

**12 792 -202.87728 4.859E-6 50589.07 -2.561E7**

**13 887 -202.87729 5.363E-7 50581.55 -2.559E7**

**CrosvalidationRun5# 11:23 Monday, September 6, 2023 148**

**The NLMIXED Procedure**

**Iteration History**

**Iter Calls NegLogLike Diff MaxGrad Slope**

**14 932 -202.87729 6.991E-8 50584.84 -2.611E7**

**15* 1026 -202.87729 5.207E-6 50557.37 -2.559E7**

**16 1080 -202.8773 7.019E-6 50585.58 -3.019E7**

**17* 1134 -202.8773 9.49E-11 50585.58 -3.019E7**

**18* 1192 -202.8773 1.94E-10 50585.58 -3.019E7**

**19* 1241 -202.8773 1.14E-13 50585.58 -3.019E7**

**NOTE: FCONV convergence criterion satisfied.**

**Fit Statistics**

**-2 Log Likelihood -405.8**

**AIC (smaller is better) -397.8**

**AICC (smaller is better) -397.4**

**BIC (smaller is better) -393.4**

**Parameter Estimates**

**Standard**

**Parameter Estimate Error DF t Value Pr > |t| Alpha Lower Upper Gradient**

**a 0.1351 0.04756 21 2.84 0.0098 0.05 0.03623 0.2340 -3.793**

**b 1.4010 0.2065 21 6.79 <.0001 0.05 0.9716 1.8304 0.996051**

**ve 0.000981 . 21 . . 0.05 . . -1.269**

**va -38.2011 . 21 . . 0.05 . . 50585.58**

**Covariance Matrix of Parameter Estimates**

**Row Parameter a b ve va**

**1 a 0.002262 -0.00974 . .**

**2 b -0.00974 0.04263 . .**

**3 ve . . . .**

**4 va . . . .**

**Correlation Matrix of Parameter Estimates**

**Row Parameter a b ve va**

**1 a 1.0000 -0.9914 . .**

**2 b -0.9914 1.0000 . .**

**3 ve . . 1.0000 .**

**4 va . . . 1.0000**

**File “Predicted x Observed P excretion Test K FOLDS”**

| **Obs** | **Study** | **FOLDS** | **PfecalurinaryBW075** | **PintakeBW075** | **InvTruSEMPfecalPolMis** | **ESTIMATED_PFfecalUrinary** |
| --- | --- | --- | --- | --- | --- | --- |
| 9 | 3 | 1 | 0.50577 | 0.66402 | 0.64767 | 0.33477 |
| 10 | 3 | 1 | 0.52644 | 0.70855 | 0.64767 | 0.35632 |
| 11 | 3 | 1 | 0.49543 | 0.69981 | 0.64767 | 0.35198 |
| 12 | 3 | 1 | 0.50020 | 0.67277 | 0.64767 | 0.33890 |
| 65 | 17 | 1 | 0.28183 | 0.45501 | 1.21869 | 0.24982 |
| 66 | 17 | 1 | 0.35204 | 0.55385 | 2.10369 | 0.28691 |
| 67 | 17 | 1 | 0.34586 | 0.50474 | 0.49499 | 0.26784 |
| 68 | 17 | 1 | 0.46194 | 0.58303 | 0.29208 | 0.29888 |
| 86 | 23 | 1 | 0.34730 | 0.58188 | 0.41000 | 0.29839 |
| 87 | 23 | 1 | 0.32513 | 0.60336 | 0.98172 | 0.30751 |
| 88 | 23 | 1 | 0.31638 | 0.57002 | 0.69027 | 0.29348 |
| 89 | 23 | 1 | 0.32811 | 0.61671 | 0.43525 | 0.31331 |
| 96 | 25 | 1 | 0.37810 | 0.51213 | 0.31387 | 0.27063 |
| 97 | 25 | 1 | 0.35730 | 0.55999 | 0.20962 | 0.28939 |
| 120 | 33 | 1 | 0.33139 | 0.54849 | 1.24443 | 0.28477 |
| 121 | 33 | 1 | 0.32644 | 0.53884 | 1.24443 | 0.28094 |
| 122 | 33 | 1 | 0.33195 | 0.53682 | 1.24443 | 0.28015 |
| 123 | 33 | 1 | 0.34998 | 0.57385 | 1.24443 | 0.29506 |
| 124 | 33 | 1 | 0.34383 | 0.56419 | 1.24443 | 0.29109 |
| 125 | 33 | 1 | 0.34934 | 0.56218 | 1.24443 | 0.29028 |
| 161 | 43 | 1 | 0.41951 | 0.52154 | 1.00000 | 0.27422 |
| 176 | 47 | 1 | 0.12229 | 0.23105 | 2.23334 | 0.18257 |
| 177 | 47 | 1 | 0.16540 | 0.36031 | 2.23334 | 0.21880 |
| 178 | 47 | 1 | 0.21704 | 0.53581 | 2.23334 | 0.27975 |
| 228 | 64 | 1 | 0.16723 | 0.29614 | 2.15889 | 0.19999 |
| 229 | 64 | 1 | 0.15765 | 0.29614 | 2.15889 | 0.19999 |
| 2 | 1 | 2 | 0.20708 | 0.32749 | 1.75046 | 0.21280 |
| 3 | 1 | 2 | 0.21833 | 0.28399 | 1.75046 | 0.20022 |
| 5 | 1 | 2 | 0.18746 | 0.25198 | 1.79908 | 0.19144 |
| 6 | 1 | 2 | 0.22965 | 0.33734 | 1.79908 | 0.21575 |
| 78 | 20 | 2 | 0.27610 | 0.44455 | 1.06452 | 0.25072 |
| 98 | 26 | 2 | 0.23321 | 0.50171 | 0.88355 | 0.27162 |
| 99 | 26 | 2 | 0.16363 | 0.54637 | 1.31873 | 0.28916 |
| 100 | 26 | 2 | 0.18226 | 0.51120 | 0.48019 | 0.27526 |
| 101 | 26 | 2 | 0.19304 | 0.48238 | 1.43666 | 0.26436 |
| 112 | 31 | 2 | 0.29338 | 0.54705 | 0.92524 | 0.28943 |
| 113 | 31 | 2 | 0.29952 | 0.55830 | 1.03627 | 0.29403 |
| 159 | 41 | 2 | 0.34513 | 0.48505 | 1.00000 | 0.26535 |
| 160 | 42 | 2 | 0.47489 | 0.65377 | 1.00000 | 0.33611 |
| 164 | 46 | 2 | 0.45325 | 0.50120 | 1.00000 | 0.27143 |
| 165 | 46 | 2 | 0.27783 | 0.50120 | 1.00000 | 0.27143 |
| 166 | 46 | 2 | 0.28552 | 0.50120 | 1.00000 | 0.27143 |
| 167 | 46 | 2 | 0.35251 | 0.50120 | 1.00000 | 0.27143 |
| 168 | 46 | 2 | 0.37649 | 0.68498 | 1.00000 | 0.35113 |
| 169 | 46 | 2 | 0.35828 | 0.68498 | 1.00000 | 0.35113 |
| 170 | 46 | 2 | 0.39127 | 0.68498 | 1.00000 | 0.35113 |
| 171 | 46 | 2 | 0.42293 | 0.68498 | 1.00000 | 0.35113 |
| 172 | 46 | 2 | 0.43120 | 0.93558 | 1.00000 | 0.49880 |
| 173 | 46 | 2 | 0.52108 | 0.93558 | 1.00000 | 0.49880 |
| 174 | 46 | 2 | 0.59568 | 0.93558 | 1.00000 | 0.49880 |
| 175 | 46 | 2 | 0.66735 | 0.93558 | 1.00000 | 0.49880 |
| 206 | 58 | 2 | 0.16929 | 0.30406 | 2.10369 | 0.20593 |
| 207 | 58 | 2 | 0.11261 | 0.17802 | 2.10369 | 0.17259 |
| 57 | 15 | 3 | 0.29169 | 0.41179 | 1.32864 | 0.23138 |
| 58 | 15 | 3 | 0.29678 | 0.45222 | 0.44177 | 0.24485 |
| 59 | 15 | 3 | 0.35205 | 0.45636 | 0.50488 | 0.24627 |
| 60 | 15 | 3 | 0.34479 | 0.54824 | 1.17806 | 0.28008 |
| 79 | 21 | 3 | 0.27610 | 0.44455 | 1.06452 | 0.24223 |
| 80 | 21 | 3 | 0.26991 | 0.35735 | 0.65448 | 0.21440 |
| 81 | 21 | 3 | 0.23739 | 0.37105 | 0.42074 | 0.21855 |
| 92 | 24 | 3 | 0.32340 | 0.42814 | 0.31276 | 0.23673 |
| 93 | 24 | 3 | 0.44623 | 0.69995 | 0.30207 | 0.34635 |
| 94 | 24 | 3 | 0.38653 | 0.53464 | 0.21816 | 0.27480 |
| 95 | 24 | 3 | 0.27202 | 0.37221 | 0.19104 | 0.21890 |
| 102 | 27 | 3 | 0.13406 | 0.26550 | 1.12554 | 0.18853 |
| 103 | 27 | 3 | 0.17787 | 0.27674 | 1.68295 | 0.19152 |
| 115 | 32 | 3 | 0.41794 | 0.57408 | 1.42508 | 0.29040 |
| 117 | 32 | 3 | 0.41289 | 0.57240 | 1.42508 | 0.28971 |
| 118 | 32 | 3 | 0.34125 | 0.45119 | 1.42508 | 0.24449 |
| 130 | 35 | 3 | 0.40220 | 0.78889 | 0.83570 | 0.39228 |
| 131 | 35 | 3 | 0.36042 | 0.63144 | 0.83570 | 0.31468 |
| 132 | 35 | 3 | 0.40240 | 0.70145 | 0.83570 | 0.34708 |
| 133 | 35 | 3 | 0.34528 | 0.62786 | 0.83570 | 0.31311 |
| 134 | 36 | 3 | 0.70340 | 0.99806 | 0.68176 | 0.52575 |
| 135 | 36 | 3 | 0.70107 | 0.99403 | 0.68176 | 0.52279 |
| 190 | 53 | 3 | 0.41778 | 0.78447 | 0.78505 | 0.38986 |
| 191 | 53 | 3 | 0.58570 | 0.84358 | 0.78505 | 0.42350 |
| 192 | 53 | 3 | 0.65032 | 0.98985 | 0.78505 | 0.51974 |
| 193 | 53 | 3 | 0.51206 | 0.93475 | 0.78505 | 0.48115 |
| 26 | 8 | 4 | 0.13471 | 0.41917 | 0.69418 | 0.23378 |
| 27 | 8 | 4 | 0.13468 | 0.41704 | 0.69418 | 0.23308 |
| 69 | 18 | 4 | 0.38032 | 0.56737 | 0.69027 | 0.28768 |
| 70 | 18 | 4 | 0.33268 | 0.54993 | 0.59100 | 0.28074 |
| 71 | 18 | 4 | 0.28821 | 0.45869 | 0.55050 | 0.24708 |
| 72 | 18 | 4 | 0.33136 | 0.51968 | 0.70684 | 0.26910 |
| 90 | 24 | 4 | 0.31780 | 0.59981 | 0.82191 | 0.30105 |
| 106 | 29 | 4 | 0.54777 | 0.73570 | 1.10241 | 0.36413 |
| 107 | 29 | 4 | 0.52358 | 0.75742 | 1.10241 | 0.37538 |
| 108 | 29 | 4 | 0.59636 | 0.76829 | 1.10241 | 0.38113 |
| 109 | 29 | 4 | 0.56288 | 0.73175 | 1.10241 | 0.36212 |
| 138 | 37 | 4 | 0.30483 | 0.64360 | 0.45450 | 0.32008 |
| 139 | 37 | 4 | 0.30655 | 0.57886 | 0.45450 | 0.29235 |
| 140 | 37 | 4 | 0.37113 | 0.76225 | 0.45450 | 0.37792 |
| 141 | 37 | 4 | 0.38049 | 0.73841 | 0.45450 | 0.36551 |
| 142 | 37 | 4 | 0.38153 | 0.74505 | 0.45450 | 0.36893 |
| 144 | 37 | 4 | 0.75155 | 1.20363 | 0.40479 | 0.70107 |
| 145 | 37 | 4 | 0.85821 | 1.22614 | 0.40479 | 0.72352 |
| 146 | 37 | 4 | 0.71682 | 1.24690 | 0.40479 | 0.74486 |
| 147 | 37 | 4 | 0.75185 | 1.19254 | 0.40479 | 0.69027 |
| 150 | 37 | 4 | 0.95856 | 1.36507 | 0.40479 | 0.87886 |
| 151 | 37 | 4 | 0.95288 | 1.37437 | 0.40479 | 0.89039 |
| 152 | 37 | 4 | 0.99734 | 1.48044 | 0.40479 | 1.03293 |
| 156 | 39 | 4 | 0.39158 | 0.61696 | 1.00000 | 0.30836 |
| 162 | 44 | 4 | 0.24599 | 0.32574 | 1.00000 | 0.20512 |
| 163 | 45 | 4 | 0.30975 | 0.49806 | 1.00000 | 0.26108 |
| 61 | 16 | 5 | 0.29807 | 0.52254 | 0.75841 | 0.28093 |
| 62 | 16 | 5 | 0.35970 | 0.54907 | 0.58903 | 0.29156 |
| 63 | 16 | 5 | 0.24236 | 0.58535 | 1.00000 | 0.30677 |
| 64 | 16 | 5 | 0.29611 | 0.57998 | 1.00000 | 0.30447 |
| 73 | 19 | 5 | 0.25244 | 0.40117 | 0.35342 | 0.23700 |
| 74 | 19 | 5 | 0.26841 | 0.42289 | 1.17806 | 0.24432 |
| 75 | 19 | 5 | 0.23244 | 0.36566 | 0.58903 | 0.22550 |
| 76 | 19 | 5 | 0.26538 | 0.39681 | 1.00000 | 0.23556 |
| 82 | 22 | 5 | 0.37174 | 0.74240 | 0.88355 | 0.38227 |
| 84 | 22 | 5 | 0.38786 | 0.71915 | 0.41191 | 0.37002 |
| 110 | 30 | 5 | 0.37654 | 0.70582 | 0.51973 | 0.36317 |
| 111 | 30 | 5 | 0.31352 | 0.57348 | 0.51973 | 0.30171 |
| 157 | 40 | 5 | 0.13251 | 0.24846 | 2.10369 | 0.19135 |
| 158 | 40 | 5 | 0.17111 | 0.37503 | 2.10369 | 0.22848 |
| 188 | 52 | 5 | 0.18066 | 0.39660 | 1.49750 | 0.23549 |
| 189 | 52 | 5 | 0.25717 | 0.55640 | 1.49750 | 0.29458 |
| 208 | 59 | 5 | 0.13447 | 0.52074 | 0.93005 | 0.28022 |
| 209 | 59 | 5 | 0.13726 | 0.49962 | 0.93005 | 0.27205 |
| 210 | 59 | 5 | 0.16952 | 0.55623 | 0.93005 | 0.29450 |
| 211 | 59 | 5 | 0.20246 | 0.67104 | 0.93005 | 0.34590 |
| 213 | 60 | 5 | 0.20391 | 0.59576 | 1.96344 | 0.31127 |
| 214 | 60 | 5 | 0.19034 | 0.57172 | 1.96344 | 0.30097 |
| 215 | 60 | 5 | 0.18600 | 0.55321 | 1.96344 | 0.29326 |
| 216 | 60 | 5 | 0.23871 | 0.59765 | 1.96344 | 0.31210 |
| 217 | 60 | 5 | 0.15977 | 0.57221 | 1.96344 | 0.30117 |

**Model Evaluation - TEST - P EXCRETION**

R version 4.3.1 (2023-06-16 ucrt) -- "Beagle Scouts"

Copyright (C) 2023 The R Foundation for Statistical Computing

Platform: x86_64-w64-mingw32/x64 (64-bit)

R is free software and comes with ABSOLUTELY NO WARRANTY.

You are welcome to redistribute it under certain conditions.

Type 'license()' or 'licence()' for distribution details.

R is a collaborative project with many contributors.

Type 'contributors()' for more information and

'citation()' on how to cite R or R packages in publications.

Type 'demo()' for some demos, 'help()' for on-line help, or

'help.start()' for an HTML browser interface to help.

Type 'q()' to quit R.

[Workspace loaded from ~/.RData]

library(metrica)

library(dplyr)

library(purrr)

library(readxl)

> attach(Predited_x_Observed_P_excretion_TEST_K_FOLDS)

The following objects are masked from Predited_x_Observed_Ca_excretion_TEST_K_FOLDS:

Residual, Study

> TEST.P_excretion <- metrics_summary(data = Predited_x_Observed_P_excretion_TEST_K_FOLDS, obs = PfecalurinaryBW075, pred = ESTIMATED_PFfecalUrinary, type = "regression")

> TEST.P_excretion

Metric Score

1 B0 4.235855e-02

2 B1 7.953867e-01

3 r 8.925657e-01

4 R2 7.966736e-01

5 Xa 9.562930e-01

6 CCC 8.535544e-01

7 MAE 6.898014e-02

8 RMAE 1.930874e-01

9 MAPE 2.261531e+01

10 SMAPE 2.167064e+01

11 RAE 5.511572e-01

12 RSE 2.436092e-01

13 MBE 3.073919e-02

14 PBE 8.604434e+00

15 PAB 1.265924e+01

16 PPB 1.718597e+01

17 MSE 7.464094e-03

18 RMSE 8.639499e-02

19 RRMSE 2.418346e-01

20 RSR 2.819715e+00

21 iqRMSE 5.062991e-01

22 MLA 2.227674e-03

23 MLP 5.236419e-03

24 RMLA 2.227674e-03

25 RMLP 5.236419e-03

26 SB 9.448976e-04

27 SDSD 1.282777e-03

28 LCS 5.236419e-03

29 PLA 2.984521e+01

30 PLP 7.015479e+01

31 Ue 7.015479e+01

32 Uc 1.718597e+01

33 Ub 1.265924e+01

34 NSE 7.563908e-01

35 E1 4.488428e-01

36 Erel 6.125616e-01

37 KGE 8.108544e-01

38 d 9.226569e-01

39 d1 6.960802e-01

40 d1r 7.244214e-01

41 RAC 9.260921e-01

42 AC 7.478783e-01

43 lambda 8.535544e-01

44 dcorr 8.488304e-01

45 MIC 6.663903e-01

**S2.2 Final Dataset, codes used to derive the Ca excretion nonlinear model, quantify study variance, and cross-validation procedure (Table 2; Figures 2 and 3; and Supplementary S3.2)**

**Abbreviations:**

Obs: observations ID

Study: Study ID

Model: Mixed (1) or Fixed (2)

Fold: FoldID (1 to 5)

CaintakeBW075 = Ca intake (g/BW^0.75^/d)

Cafecal_urinaryBW075 = Ca fecal + urinary excretion (g/BW^0.75^/d)

Inv_SEMCafecal_Norm = normalized inverse of SEM Ca fecal excretion

data FINALMODEL_Ca_Excretion;

input Obs Model Study Fold CaintakeBW075 Cafecal_urinaryBW075 Inv_SEMCafecal_Norm;

cards;

104 2 28 1 2.5368 1.8887 0.7067

138 1 37 1 1.4585 0.7983 1.0118

139 1 37 1 1.4265 0.9115 1.0118

140 1 37 1 1.7275 1.0324 1.0118

141 1 37 1 1.6723 1.0756 1.0118

142 1 37 1 1.6874 1.1085 1.0118

143 1 37 1 1.2950 1.0027 0.9029

144 1 37 1 1.7126 1.0020 0.9029

145 1 37 1 1.7145 1.1378 0.9029

146 1 37 1 1.7741 1.1296 0.9029

147 1 37 1 1.6965 1.0335 0.9029

148 1 37 1 1.3083 0.7717 0.8759

149 1 37 1 1.3602 0.8298 0.8759

150 1 37 1 1.4672 0.8375 0.8759

151 1 37 1 1.4827 0.9827 0.8759

152 1 37 1 1.5908 1.0948 0.8759

160 2 42 1 0.4701 0.3745 1.0000

9 1 3 2 1.0934 0.8612 0.7345

10 1 3 2 1.1674 0.9368 0.7345

11 1 3 2 1.1666 0.8707 0.7345

12 1 3 2 1.1404 0.8727 0.7345

71 2 18 2 2.0370 1.5525 0.2394

72 2 18 2 1.8070 1.4233 0.2387

82 2 22 2 1.1782 0.7462 1.4181

83 2 22 2 1.2747 0.7287 1.9516

84 2 22 2 1.1878 0.7071 1.1157

85 2 22 2 1.2834 0.5879 1.9516

162 2 44 2 0.6599 0.5349 1.0000

213 2 60 2 0.8919 0.3812 1.9516

216 2 60 2 0.6307 0.3475 1.9516

26 2 8 3 1.9687 0.9371 0.3432

27 2 8 3 1.9184 0.9699 0.3432

86 2 23 3 1.3705 0.9116 1.3407

87 2 23 3 1.4090 0.7509 0.7879

88 2 23 3 1.3197 0.7396 0.8475

89 2 23 3 1.5244 0.8794 0.9909

96 2 25 3 1.1190 0.8378 0.4426

97 2 25 3 1.1798 0.9079 1.1973

98 2 26 3 2.2254 0.9965 0.6369

99 2 26 3 2.0708 0.9735 1.6156

100 2 26 3 2.0731 0.9898 0.8408

112 1 31 3 0.8243 0.5221 1.6767

113 1 31 3 0.8503 0.5078 1.8931

57 2 15 4 0.6558 0.5414 1.0394

58 2 15 4 0.6573 0.4575 0.4558

59 2 15 4 0.6934 0.6259 0.3324

60 2 15 4 0.7817 0.6139 0.2968

110 2 30 4 1.3856 0.9358 0.7252

111 2 30 4 1.0847 0.7184 0.7252

130 1 35 4 1.3441 0.6045 1.2226

131 1 35 4 1.2146 0.5575 1.2226

132 1 35 4 1.3691 0.6931 1.2226

133 1 35 4 1.1902 0.5472 1.2226

157 2 40 4 0.8204 0.7829 1.9516

158 2 40 4 0.9141 0.7454 1.9516

159 2 41 4 0.7836 0.5997 1.0000

161 2 43 4 0.3855 0.2745 1.0000

65 2 17 5 1.3284 0.9910 1.3578

66 2 17 5 1.4717 1.0780 1.2391

67 2 17 5 1.6954 1.2390 0.9426

68 2 17 5 1.8734 1.4550 0.9525

80 2 21 5 1.1812 1.1951 0.7146

81 2 21 5 1.3820 1.0182 0.4254

90 2 24 5 1.0434 0.6904 1.8497

91 2 24 5 1.0655 0.8141 0.3526

92 2 24 5 0.9718 0.7980 0.2436

93 2 24 5 1.1593 0.6958 0.9525

94 2 24 5 1.2984 1.0311 0.6414

95 2 24 5 0.9863 0.8856 1.9516

163 2 45 5 0.6933 0.5396 1.0000

;

proc means;

Title Overall Descriptive Dataset;

var CaintakeBW075 Cafecal_urinaryBW075 Inv_SEMCafecal_Norm;

run;

proc means;

Title Descriptive Dataset by Fold;

var CaintakeBW075 Cafecal_urinaryBW075 Inv_SEMCafecal_Norm;

By Fold;

run;

/*parms a = 0.35 b = 0.58

ve= 0.001 va= -30;/*

PROC nLmixed ;

Title FINALMODEL_Ca_Excretion;

parms a = 0.35 b = 0.58

ve= 0.001 va= -30;

model Cafecal_urinaryBW075 ~ normal (a*exp(b*CaintakeBW075)+ u1,ve);

random u1 ~ normal(0, va) Subject=Study;

replicate Inv_SEMCafecal_Norm;

run;

Proc mixed Data = FINALMODEL_Ca_Excretion;

Title Effect Study x CaintakeBW075;

class study;

MODEL Cafecal_urinaryBW075 = CaintakeBW075 Study*CaintakeBW075/solution;

Random Study/Subject=Study;

WEIGHT Inv_SEMCafecal_Norm;

RUN;

Proc mixed Data = FINALMODEL_Ca_Excretion method=type3;

Title Study Variance and Study Effect Ca Excretion;

class study;

MODEL Cafecal_urinaryBW075 = CaintakeBW075 /solution;

RANDOM Study;;

WEIGHT Inv_SEMCafecal_Norm;

RUN;

data Test_CaCrossValidation_Run1;

input Obs Model Study Fold CaintakeBW075 Cafecal_urinaryBW075 Inv_SEMCafecal_Norm;

cards;

9 1 3 2 1.0934 0.8612 0.7345

10 1 3 2 1.1674 0.9368 0.7345

11 1 3 2 1.1666 0.8707 0.7345

12 1 3 2 1.1404 0.8727 0.7345

71 2 18 2 2.0370 1.5525 0.2394

72 2 18 2 1.8070 1.4233 0.2387

82 2 22 2 1.1782 0.7462 1.4181

83 2 22 2 1.2747 0.7287 1.9516

84 2 22 2 1.1878 0.7071 1.1157

85 2 22 2 1.2834 0.5879 1.9516

162 2 44 2 0.6599 0.5349 1.0000

213 2 60 2 0.8919 0.3812 1.9516

216 2 60 2 0.6307 0.3475 1.9516

26 2 8 3 1.9687 0.9371 0.3432

27 2 8 3 1.9184 0.9699 0.3432

86 2 23 3 1.3705 0.9116 1.3407

87 2 23 3 1.4090 0.7509 0.7879

88 2 23 3 1.3197 0.7396 0.8475

89 2 23 3 1.5244 0.8794 0.9909

96 2 25 3 1.1190 0.8378 0.4426

97 2 25 3 1.1798 0.9079 1.1973

98 2 26 3 2.2254 0.9965 0.6369

99 2 26 3 2.0708 0.9735 1.6156

100 2 26 3 2.0731 0.9898 0.8408

112 1 31 3 0.8243 0.5221 1.6767

113 1 31 3 0.8503 0.5078 1.8931

57 2 15 4 0.6558 0.5414 1.0394

58 2 15 4 0.6573 0.4575 0.4558

59 2 15 4 0.6934 0.6259 0.3324

60 2 15 4 0.7817 0.6139 0.2968

110 2 30 4 1.3856 0.9358 0.7252

111 2 30 4 1.0847 0.7184 0.7252

130 1 35 4 1.3441 0.6045 1.2226

131 1 35 4 1.2146 0.5575 1.2226

132 1 35 4 1.3691 0.6931 1.2226

133 1 35 4 1.1902 0.5472 1.2226

157 2 40 4 0.8204 0.7829 1.9516

158 2 40 4 0.9141 0.7454 1.9516

159 2 41 4 0.7836 0.5997 1.0000

161 2 43 4 0.3855 0.2745 1.0000

65 2 17 5 1.3284 0.9910 1.3578

66 2 17 5 1.4717 1.0780 1.2391

67 2 17 5 1.6954 1.2390 0.9426

68 2 17 5 1.8734 1.4550 0.9525

80 2 21 5 1.1812 1.1951 0.7146

81 2 21 5 1.3820 1.0182 0.4254

90 2 24 5 1.0434 0.6904 1.8497

91 2 24 5 1.0655 0.8141 0.3526

92 2 24 5 0.9718 0.7980 0.2436

93 2 24 5 1.1593 0.6958 0.9525

94 2 24 5 1.2984 1.0311 0.6414

95 2 24 5 0.9863 0.8856 1.9516

;

proc means;

var CaintakeBW075 Cafecal_urinaryBW075 Inv_SEMCafecal_Norm;

run;

PROC nLmixed ;

Title Test_CaCrossValidation_Run1;

parms a = 0.35 b = 0.58

ve= 0.001 va= -30;

model Cafecal_urinaryBW075 ~ normal (a*exp(b*CaintakeBW075)+ u1,ve);

random u1 ~ normal(0, va)Subject=Study;

replicate Inv_SEMCafecal_Norm;

run;

data Test_CaCrossValidation_Run2;

input Obs Model Study Fold CaintakeBW075 Cafecal_urinaryBW075 Inv_SEMCafecal_Norm;

cards;

104 2 28 1 2.5368 1.8887 0.7067

138 1 37 1 1.4585 0.7983 1.0118

139 1 37 1 1.4265 0.9115 1.0118

140 1 37 1 1.7275 1.0324 1.0118

141 1 37 1 1.6723 1.0756 1.0118

142 1 37 1 1.6874 1.1085 1.0118

143 1 37 1 1.2950 1.0027 0.9029

144 1 37 1 1.7126 1.0020 0.9029

145 1 37 1 1.7145 1.1378 0.9029

146 1 37 1 1.7741 1.1296 0.9029

147 1 37 1 1.6965 1.0335 0.9029

148 1 37 1 1.3083 0.7717 0.8759

149 1 37 1 1.3602 0.8298 0.8759

150 1 37 1 1.4672 0.8375 0.8759

151 1 37 1 1.4827 0.9827 0.8759

152 1 37 1 1.5908 1.0948 0.8759

160 2 42 1 0.4701 0.3745 1.0000

26 2 8 3 1.9687 0.9371 0.3432

27 2 8 3 1.9184 0.9699 0.3432

86 2 23 3 1.3705 0.9116 1.3407

87 2 23 3 1.4090 0.7509 0.7879

88 2 23 3 1.3197 0.7396 0.8475

89 2 23 3 1.5244 0.8794 0.9909

96 2 25 3 1.1190 0.8378 0.4426

97 2 25 3 1.1798 0.9079 1.1973

98 2 26 3 2.2254 0.9965 0.6369

99 2 26 3 2.0708 0.9735 1.6156

100 2 26 3 2.0731 0.9898 0.8408

112 1 31 3 0.8243 0.5221 1.6767

113 1 31 3 0.8503 0.5078 1.8931

57 2 15 4 0.6558 0.5414 1.0394

58 2 15 4 0.6573 0.4575 0.4558

59 2 15 4 0.6934 0.6259 0.3324

60 2 15 4 0.7817 0.6139 0.2968

110 2 30 4 1.3856 0.9358 0.7252

111 2 30 4 1.0847 0.7184 0.7252

130 1 35 4 1.3441 0.6045 1.2226

131 1 35 4 1.2146 0.5575 1.2226

132 1 35 4 1.3691 0.6931 1.2226

133 1 35 4 1.1902 0.5472 1.2226

157 2 40 4 0.8204 0.7829 1.9516

158 2 40 4 0.9141 0.7454 1.9516

159 2 41 4 0.7836 0.5997 1.0000

161 2 43 4 0.3855 0.2745 1.0000

65 2 17 5 1.3284 0.9910 1.3578

66 2 17 5 1.4717 1.0780 1.2391

67 2 17 5 1.6954 1.2390 0.9426

68 2 17 5 1.8734 1.4550 0.9525

80 2 21 5 1.1812 1.1951 0.7146

81 2 21 5 1.3820 1.0182 0.4254

90 2 24 5 1.0434 0.6904 1.8497

91 2 24 5 1.0655 0.8141 0.3526

92 2 24 5 0.9718 0.7980 0.2436

93 2 24 5 1.1593 0.6958 0.9525

94 2 24 5 1.2984 1.0311 0.6414

95 2 24 5 0.9863 0.8856 1.9516

;

proc means;

var CaintakeBW075 Cafecal_urinaryBW075 Inv_SEMCafecal_Norm;

run;

PROC nLmixed ;

Title Test_CaCrossValidation_Run2;

parms a = 0.35 b = 0.58

ve= 0.001 va= -30;

model Cafecal_urinaryBW075 ~ normal (a*exp(b*CaintakeBW075)+ u1,ve);

random u1 ~ normal(0, va)Subject=Study;

replicate Inv_SEMCafecal_Norm;

run;

data Test_CaCrossValidation_Run3;

input Obs Model Study Fold CaintakeBW075 Cafecal_urinaryBW075 Inv_SEMCafecal_Norm;

cards;

104 2 28 1 2.5368 1.8887 0.7067

138 1 37 1 1.4585 0.7983 1.0118

139 1 37 1 1.4265 0.9115 1.0118

140 1 37 1 1.7275 1.0324 1.0118

141 1 37 1 1.6723 1.0756 1.0118

142 1 37 1 1.6874 1.1085 1.0118

143 1 37 1 1.2950 1.0027 0.9029

144 1 37 1 1.7126 1.0020 0.9029

145 1 37 1 1.7145 1.1378 0.9029

146 1 37 1 1.7741 1.1296 0.9029

147 1 37 1 1.6965 1.0335 0.9029

148 1 37 1 1.3083 0.7717 0.8759

149 1 37 1 1.3602 0.8298 0.8759

150 1 37 1 1.4672 0.8375 0.8759

151 1 37 1 1.4827 0.9827 0.8759

152 1 37 1 1.5908 1.0948 0.8759

160 2 42 1 0.4701 0.3745 1.0000

9 1 3 2 1.0934 0.8612 0.7345

10 1 3 2 1.1674 0.9368 0.7345

11 1 3 2 1.1666 0.8707 0.7345

12 1 3 2 1.1404 0.8727 0.7345

71 2 18 2 2.0370 1.5525 0.2394

72 2 18 2 1.8070 1.4233 0.2387

82 2 22 2 1.1782 0.7462 1.4181

83 2 22 2 1.2747 0.7287 1.9516

84 2 22 2 1.1878 0.7071 1.1157

85 2 22 2 1.2834 0.5879 1.9516

162 2 44 2 0.6599 0.5349 1.0000

213 2 60 2 0.8919 0.3812 1.9516

216 2 60 2 0.6307 0.3475 1.9516

57 2 15 4 0.6558 0.5414 1.0394

58 2 15 4 0.6573 0.4575 0.4558

59 2 15 4 0.6934 0.6259 0.3324

60 2 15 4 0.7817 0.6139 0.2968

110 2 30 4 1.3856 0.9358 0.7252

111 2 30 4 1.0847 0.7184 0.7252

130 1 35 4 1.3441 0.6045 1.2226

131 1 35 4 1.2146 0.5575 1.2226

132 1 35 4 1.3691 0.6931 1.2226

133 1 35 4 1.1902 0.5472 1.2226

157 2 40 4 0.8204 0.7829 1.9516

158 2 40 4 0.9141 0.7454 1.9516

159 2 41 4 0.7836 0.5997 1.0000

161 2 43 4 0.3855 0.2745 1.0000

65 2 17 5 1.3284 0.9910 1.3578

66 2 17 5 1.4717 1.0780 1.2391

67 2 17 5 1.6954 1.2390 0.9426

68 2 17 5 1.8734 1.4550 0.9525

80 2 21 5 1.1812 1.1951 0.7146

81 2 21 5 1.3820 1.0182 0.4254

90 2 24 5 1.0434 0.6904 1.8497

91 2 24 5 1.0655 0.8141 0.3526

92 2 24 5 0.9718 0.7980 0.2436

93 2 24 5 1.1593 0.6958 0.9525

94 2 24 5 1.2984 1.0311 0.6414

95 2 24 5 0.9863 0.8856 1.9516

;

proc means;

var CaintakeBW075 Cafecal_urinaryBW075 Inv_SEMCafecal_Norm;

run;

PROC nLmixed ;

Title Test_CaCrossValidation_Run3;

parms a = 0.35 b = 0.58

ve= 0.001 va= -30;

model Cafecal_urinaryBW075 ~ normal (a*exp(b*CaintakeBW075)+ u1,ve);

random u1 ~ normal(0, va)Subject=Study;

replicate Inv_SEMCafecal_Norm;

run;

data Test_CaCrossValidation_Run4;

input Obs Model Study Fold CaintakeBW075 Cafecal_urinaryBW075 Inv_SEMCafecal_Norm;

cards;

104 2 28 1 2.5368 1.8887 0.7067

138 1 37 1 1.4585 0.7983 1.0118

139 1 37 1 1.4265 0.9115 1.0118

140 1 37 1 1.7275 1.0324 1.0118

141 1 37 1 1.6723 1.0756 1.0118

142 1 37 1 1.6874 1.1085 1.0118

143 1 37 1 1.2950 1.0027 0.9029

144 1 37 1 1.7126 1.0020 0.9029

145 1 37 1 1.7145 1.1378 0.9029

146 1 37 1 1.7741 1.1296 0.9029

147 1 37 1 1.6965 1.0335 0.9029

148 1 37 1 1.3083 0.7717 0.8759

149 1 37 1 1.3602 0.8298 0.8759

150 1 37 1 1.4672 0.8375 0.8759

151 1 37 1 1.4827 0.9827 0.8759

152 1 37 1 1.5908 1.0948 0.8759

160 2 42 1 0.4701 0.3745 1.0000

9 1 3 2 1.0934 0.8612 0.7345

10 1 3 2 1.1674 0.9368 0.7345

11 1 3 2 1.1666 0.8707 0.7345

12 1 3 2 1.1404 0.8727 0.7345

71 2 18 2 2.0370 1.5525 0.2394

72 2 18 2 1.8070 1.4233 0.2387

82 2 22 2 1.1782 0.7462 1.4181

83 2 22 2 1.2747 0.7287 1.9516

84 2 22 2 1.1878 0.7071 1.1157

85 2 22 2 1.2834 0.5879 1.9516

162 2 44 2 0.6599 0.5349 1.0000

213 2 60 2 0.8919 0.3812 1.9516

216 2 60 2 0.6307 0.3475 1.9516

26 2 8 3 1.9687 0.9371 0.3432

27 2 8 3 1.9184 0.9699 0.3432

86 2 23 3 1.3705 0.9116 1.3407

87 2 23 3 1.4090 0.7509 0.7879

88 2 23 3 1.3197 0.7396 0.8475

89 2 23 3 1.5244 0.8794 0.9909

96 2 25 3 1.1190 0.8378 0.4426

97 2 25 3 1.1798 0.9079 1.1973

98 2 26 3 2.2254 0.9965 0.6369

99 2 26 3 2.0708 0.9735 1.6156

100 2 26 3 2.0731 0.9898 0.8408

112 1 31 3 0.8243 0.5221 1.6767

113 1 31 3 0.8503 0.5078 1.8931

65 2 17 5 1.3284 0.9910 1.3578

66 2 17 5 1.4717 1.0780 1.2391

67 2 17 5 1.6954 1.2390 0.9426

68 2 17 5 1.8734 1.4550 0.9525

80 2 21 5 1.1812 1.1951 0.7146

81 2 21 5 1.3820 1.0182 0.4254

90 2 24 5 1.0434 0.6904 1.8497

91 2 24 5 1.0655 0.8141 0.3526

92 2 24 5 0.9718 0.7980 0.2436

93 2 24 5 1.1593 0.6958 0.9525

94 2 24 5 1.2984 1.0311 0.6414

95 2 24 5 0.9863 0.8856 1.9516

;

proc means;

var CaintakeBW075 Cafecal_urinaryBW075 Inv_SEMCafecal_Norm;

run;

PROC nLmixed ;

Title Test_CaCrossValidation_Run4;

parms a = 0.36 b = 0.58

ve= 0.001 va= -35;

model Cafecal_urinaryBW075 ~ normal (a*exp(b*CaintakeBW075)+ u1,ve);

random u1 ~ normal(0, va)Subject=Study;

replicate Inv_SEMCafecal_Norm;

run;

data Test_CaCrossValidation_Run5;

input Model Obs Study Fold CaintakeBW075 CafecalaurinaryBW075 InvTruSEMCafecalPolMis InvTruncSEMCafecalMiss;

input Obs Model Study Fold CaintakeBW075 Cafecal_urinaryBW075 Inv_SEMCafecal_Norm;

cards;

104 2 28 1 2.5368 1.8887 0.7067

138 1 37 1 1.4585 0.7983 1.0118

139 1 37 1 1.4265 0.9115 1.0118

140 1 37 1 1.7275 1.0324 1.0118

141 1 37 1 1.6723 1.0756 1.0118

142 1 37 1 1.6874 1.1085 1.0118

143 1 37 1 1.2950 1.0027 0.9029

144 1 37 1 1.7126 1.0020 0.9029

145 1 37 1 1.7145 1.1378 0.9029

146 1 37 1 1.7741 1.1296 0.9029

147 1 37 1 1.6965 1.0335 0.9029

148 1 37 1 1.3083 0.7717 0.8759

149 1 37 1 1.3602 0.8298 0.8759

150 1 37 1 1.4672 0.8375 0.8759

151 1 37 1 1.4827 0.9827 0.8759

152 1 37 1 1.5908 1.0948 0.8759

160 2 42 1 0.4701 0.3745 1.0000

9 1 3 2 1.0934 0.8612 0.7345

10 1 3 2 1.1674 0.9368 0.7345

11 1 3 2 1.1666 0.8707 0.7345

12 1 3 2 1.1404 0.8727 0.7345

71 2 18 2 2.0370 1.5525 0.2394

72 2 18 2 1.8070 1.4233 0.2387

82 2 22 2 1.1782 0.7462 1.4181

83 2 22 2 1.2747 0.7287 1.9516

84 2 22 2 1.1878 0.7071 1.1157

85 2 22 2 1.2834 0.5879 1.9516

162 2 44 2 0.6599 0.5349 1.0000

213 2 60 2 0.8919 0.3812 1.9516

216 2 60 2 0.6307 0.3475 1.9516

26 2 8 3 1.9687 0.9371 0.3432

27 2 8 3 1.9184 0.9699 0.3432

86 2 23 3 1.3705 0.9116 1.3407

87 2 23 3 1.4090 0.7509 0.7879

88 2 23 3 1.3197 0.7396 0.8475

89 2 23 3 1.5244 0.8794 0.9909

96 2 25 3 1.1190 0.8378 0.4426

97 2 25 3 1.1798 0.9079 1.1973

98 2 26 3 2.2254 0.9965 0.6369

99 2 26 3 2.0708 0.9735 1.6156

100 2 26 3 2.0731 0.9898 0.8408

112 1 31 3 0.8243 0.5221 1.6767

113 1 31 3 0.8503 0.5078 1.8931

57 2 15 4 0.6558 0.5414 1.0394

58 2 15 4 0.6573 0.4575 0.4558

59 2 15 4 0.6934 0.6259 0.3324

60 2 15 4 0.7817 0.6139 0.2968

110 2 30 4 1.3856 0.9358 0.7252

111 2 30 4 1.0847 0.7184 0.7252

130 1 35 4 1.3441 0.6045 1.2226

131 1 35 4 1.2146 0.5575 1.2226

132 1 35 4 1.3691 0.6931 1.2226

133 1 35 4 1.1902 0.5472 1.2226

157 2 40 4 0.8204 0.7829 1.9516

158 2 40 4 0.9141 0.7454 1.9516

159 2 41 4 0.7836 0.5997 1.0000

161 2 43 4 0.3855 0.2745 1.0000

;

proc means;

var CaintakeBW075 Cafecal_urinaryBW075 Inv_SEMCafecal_Norm;

run;

PROC nLmixed ;

Title Test_CaCrossValidation_Run5;

parms a = 0.38 b = 0.58

ve= 0.001 va= -25;

model Cafecal_urinaryBW075 ~ normal (a*exp(b*CaintakeBW075)+ u1,ve);

random u1 ~ normal(0, va)Subject=Study;

replicate Inv_SEMCafecal_Norm;

run;

Overall Descriptive Dataset 149

11:23 Monday, September 6, 2023

The MEANS Procedure

Variable N Mean Std Dev Minimum Maximum

ƒƒƒƒƒƒƒƒƒƒƒƒƒƒƒƒƒƒƒƒƒƒƒƒƒƒƒƒƒƒƒƒƒƒƒƒƒƒƒƒƒƒƒƒƒƒƒƒƒƒƒƒƒƒƒƒƒƒƒƒƒƒƒƒƒƒƒƒƒƒƒƒƒƒƒƒƒƒƒƒƒƒƒƒƒƒƒƒƒƒ

CaintakeBW075 70 1.3026143 0.4383595 0.3855000 2.5368000

Cafecal_urinaryBW075 70 0.8516400 0.2851178 0.2745000 1.8887000

Inv_SEMCafecal_Norm 70 1.0052029 0.4723990 0.2387000 1.9516000

ƒƒƒƒƒƒƒƒƒƒƒƒƒƒƒƒƒƒƒƒƒƒƒƒƒƒƒƒƒƒƒƒƒƒƒƒƒƒƒƒƒƒƒƒƒƒƒƒƒƒƒƒƒƒƒƒƒƒƒƒƒƒƒƒƒƒƒƒƒƒƒƒƒƒƒƒƒƒƒƒƒƒƒƒƒƒƒƒƒƒ

Descriptive Dataset by Fold 150

11:23 Monday, September 6, 2023

----------------------------------------------- Fold=1 -----------------------------------------------

The MEANS Procedure

Variable N Mean Std Dev Minimum Maximum

ƒƒƒƒƒƒƒƒƒƒƒƒƒƒƒƒƒƒƒƒƒƒƒƒƒƒƒƒƒƒƒƒƒƒƒƒƒƒƒƒƒƒƒƒƒƒƒƒƒƒƒƒƒƒƒƒƒƒƒƒƒƒƒƒƒƒƒƒƒƒƒƒƒƒƒƒƒƒƒƒƒƒƒƒƒƒƒƒƒƒ

CaintakeBW075 17 1.5518235 0.3973796 0.4701000 2.5368000

Cafecal_urinaryBW075 17 1.0006824 0.2959922 0.3745000 1.8887000

Inv_SEMCafecal_Norm 17 0.9211588 0.0810850 0.7067000 1.0118000

ƒƒƒƒƒƒƒƒƒƒƒƒƒƒƒƒƒƒƒƒƒƒƒƒƒƒƒƒƒƒƒƒƒƒƒƒƒƒƒƒƒƒƒƒƒƒƒƒƒƒƒƒƒƒƒƒƒƒƒƒƒƒƒƒƒƒƒƒƒƒƒƒƒƒƒƒƒƒƒƒƒƒƒƒƒƒƒƒƒƒ

----------------------------------------------- Fold=2 -----------------------------------------------

Variable N Mean Std Dev Minimum Maximum

ƒƒƒƒƒƒƒƒƒƒƒƒƒƒƒƒƒƒƒƒƒƒƒƒƒƒƒƒƒƒƒƒƒƒƒƒƒƒƒƒƒƒƒƒƒƒƒƒƒƒƒƒƒƒƒƒƒƒƒƒƒƒƒƒƒƒƒƒƒƒƒƒƒƒƒƒƒƒƒƒƒƒƒƒƒƒƒƒƒƒ

CaintakeBW075 13 1.1937231 0.3883186 0.6307000 2.0370000

Cafecal_urinaryBW075 13 0.8115923 0.3532915 0.3475000 1.5525000

Inv_SEMCafecal_Norm 13 1.1351000 0.6463448 0.2387000 1.9516000

ƒƒƒƒƒƒƒƒƒƒƒƒƒƒƒƒƒƒƒƒƒƒƒƒƒƒƒƒƒƒƒƒƒƒƒƒƒƒƒƒƒƒƒƒƒƒƒƒƒƒƒƒƒƒƒƒƒƒƒƒƒƒƒƒƒƒƒƒƒƒƒƒƒƒƒƒƒƒƒƒƒƒƒƒƒƒƒƒƒƒ

----------------------------------------------- Fold=3 -----------------------------------------------

Variable N Mean Std Dev Minimum Maximum

ƒƒƒƒƒƒƒƒƒƒƒƒƒƒƒƒƒƒƒƒƒƒƒƒƒƒƒƒƒƒƒƒƒƒƒƒƒƒƒƒƒƒƒƒƒƒƒƒƒƒƒƒƒƒƒƒƒƒƒƒƒƒƒƒƒƒƒƒƒƒƒƒƒƒƒƒƒƒƒƒƒƒƒƒƒƒƒƒƒƒ

CaintakeBW075 13 1.5271846 0.4787237 0.8243000 2.2254000

Cafecal_urinaryBW075 13 0.8403000 0.1662794 0.5078000 0.9965000

Inv_SEMCafecal_Norm 13 0.9966462 0.5154569 0.3432000 1.8931000

ƒƒƒƒƒƒƒƒƒƒƒƒƒƒƒƒƒƒƒƒƒƒƒƒƒƒƒƒƒƒƒƒƒƒƒƒƒƒƒƒƒƒƒƒƒƒƒƒƒƒƒƒƒƒƒƒƒƒƒƒƒƒƒƒƒƒƒƒƒƒƒƒƒƒƒƒƒƒƒƒƒƒƒƒƒƒƒƒƒƒ

----------------------------------------------- Fold=4 -----------------------------------------------

Variable N Mean Std Dev Minimum Maximum

ƒƒƒƒƒƒƒƒƒƒƒƒƒƒƒƒƒƒƒƒƒƒƒƒƒƒƒƒƒƒƒƒƒƒƒƒƒƒƒƒƒƒƒƒƒƒƒƒƒƒƒƒƒƒƒƒƒƒƒƒƒƒƒƒƒƒƒƒƒƒƒƒƒƒƒƒƒƒƒƒƒƒƒƒƒƒƒƒƒƒ

CaintakeBW075 14 0.9485786 0.3159067 0.3855000 1.3856000

Cafecal_urinaryBW075 14 0.6212643 0.1566181 0.2745000 0.9358000

Inv_SEMCafecal_Norm 14 1.0263143 0.5105435 0.2968000 1.9516000

ƒƒƒƒƒƒƒƒƒƒƒƒƒƒƒƒƒƒƒƒƒƒƒƒƒƒƒƒƒƒƒƒƒƒƒƒƒƒƒƒƒƒƒƒƒƒƒƒƒƒƒƒƒƒƒƒƒƒƒƒƒƒƒƒƒƒƒƒƒƒƒƒƒƒƒƒƒƒƒƒƒƒƒƒƒƒƒƒƒƒ

----------------------------------------------- Fold=5 -----------------------------------------------

Variable N Mean Std Dev Minimum Maximum

ƒƒƒƒƒƒƒƒƒƒƒƒƒƒƒƒƒƒƒƒƒƒƒƒƒƒƒƒƒƒƒƒƒƒƒƒƒƒƒƒƒƒƒƒƒƒƒƒƒƒƒƒƒƒƒƒƒƒƒƒƒƒƒƒƒƒƒƒƒƒƒƒƒƒƒƒƒƒƒƒƒƒƒƒƒƒƒƒƒƒ

CaintakeBW075 13 1.2423154 0.3172586 0.6933000 1.8734000

Cafecal_urinaryBW075 13 0.9562231 0.2538976 0.5396000 1.4550000

Inv_SEMCafecal_Norm 13 0.9710308 0.5276599 0.2436000 1.9516000

ƒƒƒƒƒƒƒƒƒƒƒƒƒƒƒƒƒƒƒƒƒƒƒƒƒƒƒƒƒƒƒƒƒƒƒƒƒƒƒƒƒƒƒƒƒƒƒƒƒƒƒƒƒƒƒƒƒƒƒƒƒƒƒƒƒƒƒƒƒƒƒƒƒƒƒƒƒƒƒƒƒƒƒƒƒƒƒƒƒƒ

FINALMODEL_Ca_Excretion 11:23 Monday, September 6, 2023 151

The NLMIXED Procedure

Specifications

Data Set WORK.FINALMODEL_

CA_EXCRETION

Dependent Variable Cafecal_urinaryBW075

Distribution for Dependent Variable Normal

Random Effects u1

Distribution for Random Effects Normal

Subject Variable Study

Replicate Variable Inv_SEMCafecal_Norm

Optimization Technique Dual Quasi-Newton

Integration Method Adaptive Gaussian

Quadrature

Dimensions

Observations Used 70

Observations Not Used 0

Total Observations 70

Subjects 15

Max Obs Per Subject 15

Parameters 4

Quadrature Points 1

Parameters

a b ve va NegLogLike

0.35 0.58 0.001 -30 -27.999806

Iteration History

Iter Calls NegLogLike Diff MaxGrad Slope

1 17 -97.339046 69.33924 7292.135 -1.701E8

2 65 -97.88091 0.541864 8740.717 -543985

3* 164 -97.882176 0.001266 8686.367 -764043

4 259 -98.184672 0.302496 8078.796 -754540

5 307 -98.306273 0.121602 8959.013 -1333809

6* 358 -98.306273 1.59E-11 8959.013 -1463011

NOTE: FCONV convergence criterion satisfied.

Fit Statistics

-2 Log Likelihood -196.6

AIC (smaller is better) -188.6

FINALMODEL_Ca_Excretion 11:23 Monday, September 6, 2023 152

The NLMIXED Procedure

Fit Statistics

AICC (smaller is better) -188.0

BIC (smaller is better) -185.8

Parameter Estimates

Standard

Parameter Estimate Error DF t Value Pr > |t| Alpha Lower Upper Gradient

a 0.3604 0.1438 14 2.51 0.0251 0.05 0.05203 0.6688 -12.8921

b 0.5925 0.1306 14 4.54 0.0005 0.05 0.3124 0.8726 -16.4076

ve 0.004193 . 14 . . 0.05 . . -175.81

va -34.3530 . 14 . . 0.05 . . 8959.013

Effect Study x CaintakeBW075 153

11:23 Monday, September 6, 2023

The Mixed Procedure

Model Information

Data Set WORK.FINALMODEL_

CA_EXCRETION

Dependent Variable Cafecal_urinaryBW075

Weight Variable Inv_SEMCafecal_Norm

Covariance Structure Variance Components

Subject Effect Study

Estimation Method REML

Residual Variance Method Profile

Fixed Effects SE Method Model-Based

Degrees of Freedom Method Containment

Class Level Information

Class Levels Values

Study 23 3 8 15 17 18 21 22 23 24 25 26

28 30 31 35 37 40 41 42 43 44

45 60

Dimensions

Covariance Parameters 2

Columns in X 25

Columns in Z Per Subject 23

Subjects 23

Max Obs Per Subject 15

Observations Used 70

Observations Not Used 0

Total Observations 70

Iteration History

Iteration Evaluations -2 Res Log Like Criterion

0 1 -74.17475336

1 1 -74.17475336 0.00000000

Convergence criteria met.

Effect Study x CaintakeBW075 154

11:23 Monday, September 6, 2023

The Mixed Procedure

Covariance Parameter Estimates

Cov Parm Subject Estimate

Study Study 0

Residual 0.006288

Fit Statistics

-2 Res Log Likelihood -74.2

AIC (smaller is better) -72.2

AICC (smaller is better) -72.1

BIC (smaller is better) -71.0

Solution for Fixed Effects

Standard

Effect Study Estimate Error DF t Value Pr > |t|

Intercept 0.2106 0.1146 16 1.84 0.0846

CaintakeBW075 0.1998 0.1552 30 1.29 0.2077

CaintakeBW075*Study 3 0.3910 0.08031 30 4.87 <.0001

CaintakeBW075*Study 8 0.1822 0.1129 30 1.61 0.1169

CaintakeBW075*Study 15 0.2962 0.09775 30 3.03 0.0050

CaintakeBW075*Study 17 0.4158 0.09354 30 4.45 0.0001

CaintakeBW075*Study 18 0.4644 0.1174 30 3.96 0.0004

CaintakeBW075*Study 21 0.5218 0.09622 30 5.42 <.0001

CaintakeBW075*Study 22 0.1816 0.07906 30 2.30 0.0288

CaintakeBW075*Study 23 0.2441 0.08783 30 2.78 0.0093

CaintakeBW075*Study 24 0.3520 0.07214 30 4.88 <.0001

CaintakeBW075*Study 25 0.3836 0.08839 30 4.34 0.0001

CaintakeBW075*Study 26 0.1670 0.1076 30 1.55 0.1311

CaintakeBW075*Study 28 0.4617 0.1195 30 3.86 0.0006

CaintakeBW075*Study 30 0.3025 0.09221 30 3.28 0.0026

CaintakeBW075*Study 31 0.1625 0.07279 30 2.23 0.0332

CaintakeBW075*Study 35 0.1063 0.08204 30 1.30 0.2051

CaintakeBW075*Study 37 0.2966 0.09106 30 3.26 0.0028

CaintakeBW075*Study 40 0.4354 0.07103 30 6.13 <.0001

CaintakeBW075*Study 41 0.2967 0.1138 30 2.61 0.0141

CaintakeBW075*Study 42 0.1487 0.2017 30 0.74 0.4665

CaintakeBW075*Study 43 -0.03417 0.2604 30 -0.13 0.8965

CaintakeBW075*Study 44 0.2916 0.1338 30 2.18 0.0373

CaintakeBW075*Study 45 0.2747 0.1271 30 2.16 0.0388

CaintakeBW075*Study 60 0 . . . .

Effect Study x CaintakeBW075 155

11:23 Monday, September 6, 2023

The Mixed Procedure

Type 3 Tests of Fixed Effects

Num Den

Effect DF DF F Value Pr > F

CaintakeBW075 1 30 15.85 0.0004

CaintakeBW075*Study 22 30 10.61 <.0001

Study Variance and Study Effect Ca Excretion 156

11:23 Monday, September 6, 2023

The Mixed Procedure

Model Information

Data Set WORK.FINALMODEL_

CA_EXCRETION

Dependent Variable Cafecal_urinaryBW075

Weight Variable Inv_SEMCafecal_Norm

Covariance Structure Variance Components

Estimation Method Type 3

Residual Variance Method Factor

Fixed Effects SE Method Model-Based

Degrees of Freedom Method Containment

Class Level Information

Class Levels Values

Study 23 3 8 15 17 18 21 22 23 24 25 26

28 30 31 35 37 40 41 42 43 44

45 60

Dimensions

Covariance Parameters 2

Columns in X 2

Columns in Z 23

Subjects 1

Max Obs Per Subject 70

Observations Used 70

Observations Not Used 0

Total Observations 70

Type 3 Analysis of Variance

Sum of

Source DF Squares Mean Square Expected Mean Square Error Term

CaintakeBW075 1 0.224890 0.224890 Var(Residual) + Q(CaintakeBW075) MS(Residual)

Study 22 1.456106 0.066187 Var(Residual) + 2.7718 Var(Study) MS(Residual)

Type 3 Analysis of Variance

Error

Source DF F Value Pr > F

CaintakeBW075 46 34.35 <.0001

Study 46 10.11 <.0001

Study Variance and Study Effect Ca Excretion 157

11:23 Monday, September 6, 2023

The Mixed Procedure

Type 3 Analysis of Variance

Sum of

Source DF Squares Mean Square Expected Mean Square Error Term

Residual 46 0.301188 0.006548 Var(Residual) .

Type 3 Analysis of Variance

Error

Source DF F Value Pr > F

Residual . . .

Covariance Parameter

Estimates

Cov Parm Estimate

Study 0.02152

Residual 0.006548

Fit Statistics

-2 Res Log Likelihood -85.9

AIC (smaller is better) -81.9

AICC (smaller is better) -81.7

BIC (smaller is better) -79.7

Solution for Fixed Effects

Standard

Effect Estimate Error DF t Value Pr > |t|

Intercept 0.1303 0.07067 22 1.84 0.0786

CaintakeBW075 0.5612 0.05249 46 10.69 <.0001

Type 3 Tests of Fixed Effects

Num Den

Effect DF DF F Value Pr > F

CaintakeBW075 1 46 114.31 <.0001

Study Variance and Study Effect Ca Excretion 158

11:23 Monday, September 6, 2023

Test_CaCrossValidation_Run1 159

11:23 Monday, September 6, 2023

The NLMIXED Procedure

Specifications

Data Set WORK.TEST_

CACROSSVALIDATION_

RUN1

Dependent Variable Cafecal_urinaryBW075

Distribution for Dependent Variable Normal

Random Effects u1

Distribution for Random Effects Normal

Subject Variable Study

Replicate Variable Inv_SEMCafecal_Norm

Optimization Technique Dual Quasi-Newton

Integration Method Adaptive Gaussian

Quadrature

Dimensions

Observations Used 52

Observations Not Used 0

Total Observations 52

Subjects 12

Max Obs Per Subject 6

Parameters 4

Quadrature Points 1

Parameters

a b ve va NegLogLike

0.35 0.58 0.001 -30 -26.55755

Iteration History

Iter Calls NegLogLike Diff MaxGrad Slope

1 17 -78.283724 51.72617 6994.513 -9.73E7

2 63 -78.2868 0.003076 7069.662 -468075

3* 108 -78.287077 0.000277 7107.45 -475765

4* 202 -78.287989 0.000912 7063.309 -505162

5 246 -78.288403 0.000414 7095.918 -537432

6* 290 -78.28841 7.575E-6 7099.915 -537763

7* 334 -78.288411 4.25E-7 7100.91 -537801

8* 378 -78.288411 1.523E-8 7101.159 -537810

9* 473 -78.548327 0.259916 7002.541 -504268

10 521 -78.555494 0.007168 7118.949 -4857390

11* 573 -78.556083 0.000588 7178.174 -4993306

12* 625 -78.556083 1.53E-10 7178.174 -5062911

Test_CaCrossValidation_Run1 160

11:23 Monday, September 6, 2023

The NLMIXED Procedure

Iteration History

Iter Calls NegLogLike Diff MaxGrad Slope

13* 671 -78.556083 8.53E-14 7178.174 -5062911

NOTE: FCONV convergence criterion satisfied.

Fit Statistics

-2 Log Likelihood -157.1

AIC (smaller is better) -149.1

AICC (smaller is better) -148.3

BIC (smaller is better) -147.2

Parameter Estimates

Standard

Parameter Estimate Error DF t Value Pr > |t| Alpha Lower Upper Gradient

a 0.3617 0.1996 11 1.81 0.0973 0.05 -0.07764 0.8010 -6.25542

b 0.5943 0.1832 11 3.24 0.0078 0.05 0.1912 0.9975 -8.54417

ve 0.004129 . 11 . . 0.05 . . -12.1513

va -37.1309 . 11 . . 0.05 . . 7178.174

Test_CaCrossValidation_Run1 161

11:23 Monday, September 6, 2023

The MEANS Procedure

Variable N Mean Std Dev Minimum Maximum

ƒƒƒƒƒƒƒƒƒƒƒƒƒƒƒƒƒƒƒƒƒƒƒƒƒƒƒƒƒƒƒƒƒƒƒƒƒƒƒƒƒƒƒƒƒƒƒƒƒƒƒƒƒƒƒƒƒƒƒƒƒƒƒƒƒƒƒƒƒƒƒƒƒƒƒƒƒƒƒƒƒƒƒƒƒƒƒƒƒƒ

CaintakeBW075 56 1.3387732 0.4441713 0.3855000 2.5368000

Cafecal_urinaryBW075 56 0.8665089 0.2690313 0.2745000 1.8887000

Inv_SEMCafecal_Norm 56 0.9751411 0.4288456 0.2436000 1.9516000

ƒƒƒƒƒƒƒƒƒƒƒƒƒƒƒƒƒƒƒƒƒƒƒƒƒƒƒƒƒƒƒƒƒƒƒƒƒƒƒƒƒƒƒƒƒƒƒƒƒƒƒƒƒƒƒƒƒƒƒƒƒƒƒƒƒƒƒƒƒƒƒƒƒƒƒƒƒƒƒƒƒƒƒƒƒƒƒƒƒƒ

Test_CaCrossValidation_Run2 162

11:23 Monday, September 6, 2023

The NLMIXED Procedure

Specifications

Data Set WORK.TEST_

CACROSSVALIDATION_

RUN2

Dependent Variable Cafecal_urinaryBW075

Distribution for Dependent Variable Normal

Random Effects u1

Distribution for Random Effects Normal

Subject Variable Study

Replicate Variable Inv_SEMCafecal_Norm

Optimization Technique Dual Quasi-Newton

Integration Method Adaptive Gaussian

Quadrature

Dimensions

Observations Used 56

Observations Not Used 0

Total Observations 56

Subjects 11

Max Obs Per Subject 15

Parameters 4

Quadrature Points 1

Parameters

a b ve va NegLogLike

0.35 0.58 0.001 -30 -9.1792616

Iteration History

Iter Calls NegLogLike Diff MaxGrad Slope

1 17 -72.827051 63.64779 6098.721 -1.269E8

2 62 -72.827055 3.911E-6 6101.608 -357245

3* 107 -72.827055 3.249E-8 6101.969 -357512

4* 203 -73.195903 0.368848 5346.236 -372357

5 260 -73.516293 0.32039 6043.173 -507006

6* 313 -73.532698 0.016405 6245.437 -557062

7* 364 -73.53271 0.000012 6251.539 -568661

8* 420 -73.53271 6.87E-11 6251.539 -568990

9* 477 -73.53271 1.14E-13 6251.539 -568990

Test_CaCrossValidation_Run2 163

11:23 Monday, September 6, 2023

The NLMIXED Procedure

NOTE: FCONV convergence criterion satisfied.

Fit Statistics

-2 Log Likelihood -147.1

AIC (smaller is better) -139.1

AICC (smaller is better) -138.3

BIC (smaller is better) -137.5

Parameter Estimates

Standard

Parameter Estimate Error DF t Value Pr > |t| Alpha Lower Upper Gradient

a 0.3634 0.1232 10 2.95 0.0145 0.05 0.08896 0.6378 -18.5999

b 0.5952 0.1121 10 5.31 0.0003 0.05 0.3455 0.8450 -21.6864

ve 0.004639 . 10 . . 0.05 . . -0.19086

va -33.0531 . 10 . . 0.05 . . 6251.539

Test_CaCrossValidation_Run2 164

11:23 Monday, September 6, 2023

Test_CaCrossValidation_Run3 165

11:23 Monday, September 6, 2023

The NLMIXED Procedure

Specifications

Data Set WORK.TEST_

CACROSSVALIDATION_

RUN3

Dependent Variable Cafecal_urinaryBW075

Distribution for Dependent Variable Normal

Random Effects u1

Distribution for Random Effects Normal

Subject Variable Study

Replicate Variable Inv_SEMCafecal_Norm

Optimization Technique Dual Quasi-Newton

Integration Method Adaptive Gaussian

Quadrature

Dimensions

Observations Used 56

Observations Not Used 0

Total Observations 56

Subjects 12

Max Obs Per Subject 15

Parameters 4

Quadrature Points 1

Parameters

a b ve va NegLogLike

0.35 0.58 0.001 -30 -4.4270873

Iteration History

Iter Calls NegLogLike Diff MaxGrad Slope

1 17 -75.22014 70.79305 5980.711 -1.514E8

2 62 -75.234828 0.014687 6154.401 -348490

3* 107 -75.234931 0.000103 6176.734 -364819

4* 201 -75.247356 0.012425 5938.61 -381532

5 245 -75.269333 0.021977 6118.445 -402328

6* 289 -75.270651 0.001318 6162.318 -404968

7* 332 -75.270738 0.000087 6173.076 -405426

8* 375 -75.270747 8.967E-6 6178.414 -405527

9* 465 -75.71116 0.440413 5432.087 -381739

10 518 -76.040584 0.329424 6139.3 -659829

11* 566 -76.056137 0.015554 6345.663 -747766

12* 664 -76.05653 0.000393 6317.916 -402676

Test_CaCrossValidation_Run3 166

11:23 Monday, September 6, 2023

The NLMIXED Procedure

Iteration History

Iter Calls NegLogLike Diff MaxGrad Slope

13 707 -76.056721 0.000191 6341.36 -438237

14* 797 -76.064615 0.007893 6186.848 -402130

15 849 -76.07346 0.008845 6385.15 -597006

16* 905 -76.07346 8E-12 6385.15 -610218

NOTE: FCONV convergence criterion satisfied.

Fit Statistics

-2 Log Likelihood -152.1

AIC (smaller is better) -144.1

AICC (smaller is better) -143.4

BIC (smaller is better) -142.2

Parameter Estimates

Standard

Parameter Estimate Error DF t Value Pr > |t| Alpha Lower Upper Gradient

a 0.3726 0.2151 11 1.73 0.1112 0.05 -0.1009 0.8461 -5.75147

b 0.6081 0.1914 11 3.18 0.0088 0.05 0.1867 1.0295 -9.42124

ve 0.004759 . 11 . . 0.05 . . -41.3547

va -37.1925 . 11 . . 0.05 . . 6385.15

Test_CaCrossValidation_Run3 167

11:23 Monday, September 6, 2023

Test_CaCrossValidation_Run4 168

11:23 Monday, September 6, 2023

The NLMIXED Procedure

Specifications

Data Set WORK.TEST_

CACROSSVALIDATION_

RUN4

Dependent Variable Cafecal_urinaryBW075

Distribution for Dependent Variable Normal

Random Effects u1

Distribution for Random Effects Normal

Subject Variable Study

Replicate Variable Inv_SEMCafecal_Norm

Optimization Technique Dual Quasi-Newton

Integration Method Adaptive Gaussian

Quadrature

Dimensions

Observations Used 55

Observations Not Used 0

Total Observations 55

Subjects 9

Max Obs Per Subject 15

Parameters 4

Quadrature Points 1

Parameters

a b ve va NegLogLike

0.36 0.58 0.001 -35 -0.3566251

Iteration History

Iter Calls NegLogLike Diff MaxGrad Slope

1 17 -74.308283 73.95166 5822.291 -1.609E8

2 62 -74.321635 0.013352 5979.04 -330617

3* 107 -74.321815 0.00018 5999.192 -345066

4* 152 -74.321815 9.368E-8 5999.822 -346932

5* 245 -74.44582 0.124005 5512.81 -359982

6 298 -74.564601 0.118781 6190.795 -738104

7* 349 -74.564601 1.39E-11 6190.795 -841686

NOTE: FCONV convergence criterion satisfied.

Test_CaCrossValidation_Run4 169

11:23 Monday, September 6, 2023

The NLMIXED Procedure

Fit Statistics

-2 Log Likelihood -149.1

AIC (smaller is better) -141.1

AICC (smaller is better) -140.3

BIC (smaller is better) -140.3

Parameter Estimates

Standard

Parameter Estimate Error DF t Value Pr > |t| Alpha Lower Upper Gradient

a 0.3718 0.2600 8 1.43 0.1906 0.05 -0.2277 0.9714 -3.58829

b 0.5951 0.2232 8 2.67 0.0285 0.05 0.08041 1.1099 -5.76533

ve 0.004902 . 8 . . 0.05 . . -144.596

va -41.0029 . 8 . . 0.05 . . 6190.795

Test_CaCrossValidation_Run4 170

11:23 Monday, September 6, 2023

Test_CaCrossValidation_Run5 171

11:23 Monday, September 6, 2023

The NLMIXED Procedure

Specifications

Data Set WORK.TEST_

CACROSSVALIDATION_

RUN5

Dependent Variable Cafecal_urinaryBW075

Distribution for Dependent Variable Normal

Random Effects u1

Distribution for Random Effects Normal

Subject Variable Study

Replicate Variable Inv_SEMCafecal_Norm

Optimization Technique Dual Quasi-Newton

Integration Method Adaptive Gaussian

Quadrature

Dimensions

Observations Used 19

Observations Not Used 0

Total Observations 19

Subjects 8

Max Obs Per Subject 5

Parameters 4

Quadrature Points 1

Parameters

a b ve va NegLogLike

0.38 0.58 0.001 -25 -23.948986

Iteration History

Iter Calls NegLogLike Diff MaxGrad Slope

1 15 -32.169284 8.220298 3100.134 -4947428

2 63 -32.361626 0.192342 3697.617 -97766

3* 108 -32.363949 0.002323 3782.316 -123952

4* 204 -32.364001 0.000052 3765.981 -143060

5 246 -32.364035 0.000035 3772.947 -148693

6* 288 -32.364039 3.656E-6 3776.401 -148718

7* 376 -32.369015 0.004976 3671.277 -142612

8 419 -32.373422 0.004406 3791.534 -264410

9* 514 -32.373505 0.000083 3775.739 -143759

10 556 -32.37351 5.241E-6 3779.194 -146104

11* 599 -32.37351 3.098E-8 3779.409 -146112

12* 642 -32.37351 2.575E-9 3779.516 -146112

Test_CaCrossValidation_Run5 172

11:23 Monday, September 6, 2023

The NLMIXED Procedure

Iteration History

Iter Calls NegLogLike Diff MaxGrad Slope

13* 735 -32.404044 0.030534 3738.875 -142848

14 781 -32.404992 0.000948 3777.743 -1527632

15* 827 -32.405052 0.00006 3787.555 -1555841

16* 873 -32.405055 3.695E-6 3790.013 -1562994

17* 919 -32.405056 2.221E-7 3790.627 -1564788

18* 965 -32.405056 1.167E-8 3790.781 -1565237

19* 1011 -32.405056 2.26E-10 3790.8 -1565349

20* 1057 -32.405056 1.37E-11 3790.805 -1565363

21* 1100 -32.405056 7.6E-13 3790.806 -1565366

NOTE: FCONV convergence criterion satisfied.

Fit Statistics

-2 Log Likelihood -64.8

AIC (smaller is better) -56.8

AICC (smaller is better) -54.0

BIC (smaller is better) -56.5

Parameter Estimates

Standard

Parameter Estimate Error DF t Value Pr > |t| Alpha Lower Upper Gradient

a 0.3842 0.3595 7 1.07 0.3206 0.05 -0.4658 1.2342 -2.51018

b 0.5864 0.3041 7 1.93 0.0952 0.05 -0.1327 1.3055 -4.10527

ve 0.002817 . 7 . . 0.05 . . 0.049584

va -29.7382 . 7 . . 0.05 . . 3790.806

**File “Predicted x Observed Ca excretion – TEST K - FOLD**

| Obs | Model | Study | Fold | CaintakeBW075 | Cafecal_urinaryBW075 | Inv_SEMCafecal_Norm | Estimated_CaFecalUrinary |
| --- | --- | --- | --- | --- | --- | --- | --- |
| 104 | 2 | 28 | 1 | 2.5368 | 1.8887 | 0.7067 | 1.6334 |
| 138 | 1 | 37 | 1 | 1.4585 | 0.7983 | 1.0118 | 0.8606 |
| 139 | 1 | 37 | 1 | 1.4265 | 0.9115 | 1.0118 | 0.8444 |
| 140 | 1 | 37 | 1 | 1.7275 | 1.0324 | 1.0118 | 1.0098 |
| 141 | 1 | 37 | 1 | 1.6723 | 1.0756 | 1.0118 | 0.9771 |
| 142 | 1 | 37 | 1 | 1.6874 | 1.1085 | 1.0118 | 0.9860 |
| 143 | 1 | 37 | 1 | 1.2950 | 1.0027 | 0.9029 | 0.7809 |
| 144 | 1 | 37 | 1 | 1.7126 | 1.0020 | 0.9029 | 1.0008 |
| 145 | 1 | 37 | 1 | 1.7145 | 1.1378 | 0.9029 | 1.0020 |
| 146 | 1 | 37 | 1 | 1.7741 | 1.1296 | 0.9029 | 1.0381 |
| 147 | 1 | 37 | 1 | 1.6965 | 1.0335 | 0.9029 | 0.9913 |
| 148 | 1 | 37 | 1 | 1.3083 | 0.7717 | 0.8759 | 0.7871 |
| 149 | 1 | 37 | 1 | 1.3602 | 0.8298 | 0.8759 | 0.8118 |
| 150 | 1 | 37 | 1 | 1.4672 | 0.8375 | 0.8759 | 0.8651 |
| 151 | 1 | 37 | 1 | 1.4827 | 0.9827 | 0.8759 | 0.8730 |
| 152 | 1 | 37 | 1 | 1.5908 | 1.0948 | 0.8759 | 0.9310 |
| 160 | 2 | 42 | 1 | 0.4701 | 0.3745 | 1.0000 | 0.4783 |
| 9 | 1 | 3 | 2 | 1.0934 | 0.8612 | 0.7345 | 0.6967 |
| 10 | 1 | 3 | 2 | 1.1674 | 0.9368 | 0.7345 | 0.7280 |
| 11 | 1 | 3 | 2 | 1.1666 | 0.8707 | 0.7345 | 0.7277 |
| 12 | 1 | 3 | 2 | 1.1404 | 0.8727 | 0.7345 | 0.7164 |
| 71 | 2 | 18 | 2 | 2.0370 | 1.5525 | 0.2394 | 1.2216 |
| 72 | 2 | 18 | 2 | 1.8070 | 1.4233 | 0.2387 | 1.0653 |
| 82 | 2 | 22 | 2 | 1.1782 | 0.7462 | 1.4181 | 0.7327 |
| 83 | 2 | 22 | 2 | 1.2747 | 0.7287 | 1.9516 | 0.7760 |
| 84 | 2 | 22 | 2 | 1.1878 | 0.7071 | 1.1157 | 0.7369 |
| 85 | 2 | 22 | 2 | 1.2834 | 0.5879 | 1.9516 | 0.7801 |
| 162 | 2 | 44 | 2 | 0.6599 | 0.5349 | 1.0000 | 0.5382 |
| 213 | 2 | 60 | 2 | 0.8919 | 0.3812 | 1.9516 | 0.6179 |
| 216 | 2 | 60 | 2 | 0.6307 | 0.3475 | 1.9516 | 0.5289 |
| 26 | 2 | 8 | 3 | 1.9687 | 0.9371 | 0.3432 | 1.2336 |
| 27 | 2 | 8 | 3 | 1.9184 | 0.9699 | 0.3432 | 1.1964 |
| 86 | 2 | 23 | 3 | 1.3705 | 0.9116 | 1.3407 | 0.8574 |
| 87 | 2 | 23 | 3 | 1.4090 | 0.7509 | 0.7879 | 0.8777 |
| 88 | 2 | 23 | 3 | 1.3197 | 0.7396 | 0.8475 | 0.8313 |
| 89 | 2 | 23 | 3 | 1.5244 | 0.8794 | 0.9909 | 0.9415 |
| 96 | 2 | 25 | 3 | 1.1190 | 0.8378 | 0.4426 | 0.7358 |
| 97 | 2 | 25 | 3 | 1.1798 | 0.9079 | 1.1973 | 0.7635 |
| 98 | 2 | 26 | 3 | 2.2254 | 0.9965 | 0.6369 | 1.4420 |
| 99 | 2 | 26 | 3 | 2.0708 | 0.9735 | 1.6156 | 1.3126 |
| 100 | 2 | 26 | 3 | 2.0731 | 0.9898 | 0.8408 | 1.3145 |
| 112 | 1 | 31 | 3 | 0.8243 | 0.5221 | 1.6767 | 0.6151 |
| 113 | 1 | 31 | 3 | 0.8503 | 0.5078 | 1.8931 | 0.6249 |
| 57 | 2 | 15 | 4 | 0.6558 | 0.5414 | 1.0394 | 0.5493 |
| 58 | 2 | 15 | 4 | 0.6573 | 0.4575 | 0.4558 | 0.5498 |
| 59 | 2 | 15 | 4 | 0.6934 | 0.6259 | 0.3324 | 0.5617 |
| 60 | 2 | 15 | 4 | 0.7817 | 0.6139 | 0.2968 | 0.5920 |
| 110 | 2 | 30 | 4 | 1.3856 | 0.9358 | 0.7252 | 0.8481 |
| 111 | 2 | 30 | 4 | 1.0847 | 0.7184 | 0.7252 | 0.7090 |
| 130 | 1 | 35 | 4 | 1.3441 | 0.6045 | 1.2226 | 0.8273 |
| 131 | 1 | 35 | 4 | 1.2146 | 0.5575 | 1.2226 | 0.7660 |
| 132 | 1 | 35 | 4 | 1.3691 | 0.6931 | 1.2226 | 0.8397 |
| 133 | 1 | 35 | 4 | 1.1902 | 0.5472 | 1.2226 | 0.7550 |
| 157 | 2 | 40 | 4 | 0.8204 | 0.7829 | 1.9516 | 0.6058 |
| 158 | 2 | 40 | 4 | 0.9141 | 0.7454 | 1.9516 | 0.6406 |
| 159 | 2 | 41 | 4 | 0.7836 | 0.5997 | 1.0000 | 0.5927 |
| 161 | 2 | 43 | 4 | 0.3855 | 0.2745 | 1.0000 | 0.4677 |
| 65 | 2 | 17 | 5 | 1.3284 | 0.9910 | 1.3578 | 0.7965 |
| 66 | 2 | 17 | 5 | 1.4717 | 1.0780 | 1.2391 | 0.9107 |
| 67 | 2 | 17 | 5 | 1.6954 | 1.2390 | 0.9426 | 1.0383 |
| 68 | 2 | 17 | 5 | 1.8734 | 1.4550 | 0.9525 | 1.1525 |
| 80 | 2 | 21 | 5 | 1.1812 | 1.1951 | 0.7146 | 0.7680 |
| 81 | 2 | 21 | 5 | 1.3820 | 1.0182 | 0.4254 | 0.8640 |
| 90 | 2 | 24 | 5 | 1.0434 | 0.6904 | 1.8497 | 0.7084 |
| 91 | 2 | 24 | 5 | 1.0655 | 0.8141 | 0.3526 | 0.7176 |
| 92 | 2 | 24 | 5 | 0.9718 | 0.7980 | 0.2436 | 0.6793 |
| 93 | 2 | 24 | 5 | 1.1593 | 0.6958 | 0.9525 | 0.7582 |
| 94 | 2 | 24 | 5 | 1.2984 | 1.0311 | 0.6414 | 0.8227 |
| 95 | 2 | 24 | 5 | 0.9863 | 0.8856 | 1.9516 | 0.6851 |
| 163 | 2 | 45 | 5 | 0.6933 | 0.5396 | 1.0000 | 0.5769 |

**MODEL EVALUATION Ca EXCRETION**

| R version 4.3.1 (2023-06-16 ucrt) -- "Beagle Scouts"  Copyright (C) 2023 The R Foundation for Statistical Computing  Platform: x86_64-w64-mingw32/x64 (64-bit)  R is free software and comes with ABSOLUTELY NO WARRANTY.  You are welcome to redistribute it under certain conditions.  Type 'license()' or 'licence()' for distribution details.  R is a collaborative project with many contributors.  Type 'contributors()' for more information and  'citation()' on how to cite R or R packages in publications.  Type 'demo()' for some demos, 'help()' for on-line help, or  'help.start()' for an HTML browser interface to help.  Type 'q()' to quit R.  [Workspace loaded from ~/.RData]  > library(metrica)  > library(dplyr)  Attaching package: ‘dplyr’  The following objects are masked from ‘package:stats’:  filter, lag  The following objects are masked from ‘package:base’:  intersect, setdiff, setequal, union  library(purrr)  > library(readxl)  > library(readxl)  > View(Predited_x_Observed_Ca_excretion_TEST_K_FOLDS)  > attach(Predited_x_Observed_Ca_excretion_TEST_K_FOLDS)  > TEST.Caexcretion <- metrics_summary(data = Predited_x_Observed_Ca_excretion_TEST_K_FOLDS, obs = Cafecal_urinaryBW075, pred = Estimated_CaFecalUrinary, type = "regression")  > TEST.Caexcretion  Metric Score  1 B0 0.146327817  2 B1 0.805560635  3 r 0.790895521  4 R2 0.625515726  5 Xa 0.974335286  6 CCC 0.770597414  7 MAE 0.139825372  8 RMAE 0.164183372  9 MAPE 17.407167445  10 SMAPE 16.659746534  11 RAE 0.652148449  12 RSE 0.379330802  13 MBE 0.019264813  14 PBE 2.262080178  15 PAB 1.220956069  16 PPB 9.966674592  17 MSE 0.030396920  18 RMSE 0.174347124  19 RRMSE 0.204718917  20 RSR 2.175721598  21 iqRMSE 0.563266208  22 MLA 0.003400695  23 MLP 0.026996224  24 RMLA 0.003400695  25 RMLP 0.026996224  26 SB 0.000371133  27 SDSD 0.003029562  28 LCS 0.026996224  29 PLA 11.187630661  30 PLP 88.812369339  31 Ue 88.812369339  32 Uc 9.966674592  33 Ub 1.220956069  34 NSE 0.620669198  35 E1 0.347851551  36 Erel 0.542337950  37 KGE 0.725882629  38 d 0.873443325  39 d1 0.641008197  40 d1r 0.673925776  41 RAC 0.885137848  42 AC 0.499971802  43 lambda 0.770597414  44 dcorr 0.772295420  45 MIC 0.615802100 |
| --- |

**S2.3** **Final Dataset, and codes used to derive Phosphorous and Calcium Metabolizable Coefficient (Table 3; Figure 4)**

**Abbreviations:**

Obs: observations ID

Study: Study ID

Ca_P_Ratio = Ca/P ratio diet

Foragediet = Forage in diet (g/kg DM)

CPdiet = Crude protein in diet (g/kg DM)

NDFdiet = Neutral detergent fiber in diet (g/kg DM)

EEdiet = Ether extract in diet (g/kg DM)

StarchDiet = Starch in diet (g/kg DM)

InverseTruncSEMPfecalPolled = normalized inverse of SEM P fecal excretion

InverseTruncSEMCafecalPolled = normalized inverse of SEM Ca fecal excretion

BW = body weight (kg)

DMI = dry matter intake (kg/d)

MY = milk yield (kg/d)

Pintake = Phosphorous intake (g/d)

Pfecal = Phosphorous fecal excretion (g/d)

Purinary = Phosphorous urinary excretion (g/d)

Caintake = Calcium intake (g/d)

Cafecal = Calcium fecal excretion (g/d)

Caurinary = Calcium urinary excretion (g/d)

Est_End_P = Estimated phosphorous endogenous fecal and urinary excretion (g/d = 0.1352 × BW^0.75^)

Est_End_Ca = Estimated calcium endogenous fecal and urinary excretion (g/d = 0.3604 × BW^0.75^)

MetabolazibleP = Metabolizable coefficient of phosphorous (0 to 1) = (Pintake – Pfecal – Purinary + Est_End_P) / Pintake

MetabolazibleCa = Metabolizable coefficient of calcium (0 to 1) = (Caintake – Cafecal – Caurinary + Est_End_Ca) / Caintake

**INPUT CODE**

data P and Ca Metabolizable;

input Obs Study Ca_P_Ratio Foragediet CPdiet NDFdiet EEdiet StarchDiet InverseTruncSEMPfecalPolled InverseTruncSEMCafecalPolled BW DMI MY Pintake Pfecal Purinary Caintake

MetabolazibleCa ;

cards;

1 1 . . . . . . 1.672 . . . . 52 30.8 0.17 . . . . . . .

2 1 . . . . . . 1.672 . 604 . 27.9 39.9 25.1 0.13 . . . 16.47 43.91 0.781 .

3 1 . . . . . . 1.672 . 604 . 27.9 34.6 26.4 0.2 . . . 16.47 43.91 0.707 .

4 1 . . . . . . 1.719 . . . . 42.4 22 0.21 . . . . . . .

5 1 . . . . . . 1.719 . 604 . 27.9 30.7 22.6 0.24 . . . 16.47 . 0.793 .

6 1 . . . . . . 1.719 . 604 . 27.9 41.1 27.8 0.18 . . . 16.47 43.91 0.720 .

7 2 2.60 957 153 433 . . 0.990 . 604 9.38 28.53 30.7 24.2 . . . . . 43.91 . .

8 2 1.93 957 151 430 . . 0.990 . 605 8.7 28.57 35.7 29.2 . . . . . 43.96 . .

9 3 1.67 582 177 362 . . 0.619 0.829 630 20.1 29.2 83.5 60.4 3.2 137.5 105.5 2.8 17.00 45.32 . 0.542

10 3 1.63 582 173 359 . . 0.619 0.829 630 21.1 32.2 89.1 65.4 0.8 146.8 116.6 1.2 17.00 45.32 . 0.506

11 3 1.67 584 181 366 . . 0.619 0.829 630 20.6 31.3 88 61.6 0.7 146.7 108.5 0.99 17.00 45.32 . 0.563

12 3 1.67 571 178 382 . . 0.619 0.829 630 20.1 30.3 84.6 60.5 2.4 143.4 109.1 0.64 17.00 45.32 . 0.551

13 4 2.21 . 165 347 44 . 1.450 . 644 . . 74 45.6 . . . . 17.28 46.07 0.617 .

14 4 2.21 . 165 347 44 . 1.450 . 644 . . 72.9 51 . . . . 17.28 46.07 0.538 .

15 4 2.21 . 165 347 44 . 1.450 . 644 . . 72.6 52.1 . . . . 17.28 46.07 0.520 .

16 4 2.21 . 165 347 44 . 1.450 . 644 . . 71.5 49.8 . . . . 17.28 46.07 0.545 .

17 5 . 690 . . . . 1.000 . 633 17.4 24.5 68 29 . . . . 17.06 45.48 0.824 .

18 5 . 691 . . . . 1.000 . 645 17.7 25.4 60 30 . . . . 17.30 46.13 0.788 .

19 6 . . . . . . . . . . . 30.3 29.2 1.22 64.8 46.3 0.2 . . . .

20 6 . . . . . . . . . . . 27.9 21.9 3.69 59.4 39.5 0.6 . . . .

21 6 . . . . . . . . . . . 26 14.8 4.73 54.1 29.9 0.3 . . . .

22 7 3.00 440 180 403 47 169 1.238 . 663 20.1 31.3 71.3 41.8 . . . . 17.66 47.09 0.661 .

23 7 3.13 534 170 482 44 95 1.238 . 655 17.6 28.9 55.7 36.2 . . . . 17.50 46.66 0.664 .

24 7 2.47 449 173 311 46 304 1.238 . 649 23.4 32.7 83.7 36.7 . . . . 17.38 46.34 0.769 .

25 7 2.58 544 174 342 45 278 1.238 . 656 23.2 30.9 82 41.9 . . . . 17.52 46.72 0.703 .

26 8 5.04 600 186 350 . . 0.689 0.313 518 16.7 23.36 45.5 14.6 0.0221 213.7 101.2 0.524 14.68 39.12 1.001 0.707

27 8 4.65 550 170 356 . . 0.689 0.313 514 16.7 23.15 45 14.5 0.0319 207 104.4 0.256 14.59 38.89 1.001 0.682

28 9 1.54 475 174 378 39 130 0.775 . 577 20.6 35.2 83.6 47 . . . . 15.92 42.43 0.628 .

29 9 1.49 475 178 362 41 146 0.827 . 569 21.9 35.7 88.7 52.8 . . . . 15.75 41.99 0.582 .

30 9 1.49 475 178 363 41 145 0.856 . 564 19.2 31.1 78.7 46.1 . . . . 15.65 41.71 0.613 .

31 9 1.49 475 163 402 38 127 0.591 . 608 14.4 27.4 58.9 34.9 . . . . 16.55 44.13 0.689 .

32 9 1.75 475 135 496 28 59 0.620 . 627 10.4 15.4 33.7 23.4 . . . . 16.94 45.16 0.808 .

33 9 2.06 475 173 383 39 133 0.775 . 614 22.3 36.1 70.4 34.4 . . . . 16.68 44.45 0.748 .

34 9 2.03 475 176 372 41 145 0.827 . 614 22.7 36.1 70.4 33 . . . . 16.68 44.45 0.768 .

35 9 1.51 475 176 373 41 144 0.856 . 598 18.8 30.7 77.4 44.1 . . . . 16.35 43.58 0.641 .

36 9 1.58 475 157 427 36 112 0.591 . 652 13.3 25.6 52.5 32.6 . . . . 17.44 46.50 0.711 .

37 9 1.75 475 136 496 29 63 0.620 . 691 10.7 17.2 35.9 26.2 . . . . 18.22 48.57 0.778 .

38 10 . . . . . . 1.862 . 638 23.3 32.8 91.5 45.8 . . . . 17.16 45.75 0.687 .

39 10 . . . . . . 1.862 . 638 23.3 34 82.4 39.7 . . . . 17.16 45.75 0.726 .

40 10 . . . . . . 1.862 . 638 23.3 31.1 85.4 44.8 . . . . 17.16 45.75 0.676 .

41 10 . . . . . . 1.862 . 638 23.3 31.3 93 44.4 . . . . 17.16 45.75 0.707 .

42 11 . 482 172 338 . . 1.125 . . 23.2 33.8 77.1 47.4 0.5 . . . . . . .

43 11 . 480 165 335 . . 1.125 . . 22.3 33.6 71.8 45.9 0.5 . . . . . . .

44 11 . 479 172 330 . . 1.125 . . 21.9 36.5 87.7 53.5 0.5 . . . . . . .

45 12 1.69 605 150 347 . . 1.996 . . 23.1 31.6 90.9 51.4 . . . . . . . .

46 12 1.69 605 150 347 . . 1.996 . . 22.3 31.6 87.9 49.9 . . . . . . . .

47 12 1.69 605 150 347 . . 1.996 . . 23.1 32 90.7 53.4 . . . . . . . .

48 12 1.69 605 150 347 . . 1.996 . . 22.7 31.5 89.1 52.3 . . . . . . . .

49 13 . . . . . . 1.339 . . . . 103.2 75 0.4 . . . . . . .

50 13 . . . . . . 1.339 . . . . 72.2 41.2 0.2 . . . . . . .

51 13 . . . . . . 0.487 . . . . 105.1 62.5 0.3 . . . . . . .

52 13 . . . . . . 0.487 . . . . 71.3 42.6 0.1 . . . . . . .

53 14 2.23 610 172 317 . 245 0.952 . . 25.1 33.8 87.3 49.8 0.34 . . . . . . .

54 14 1.80 610 172 315 . 244 0.952 . . 26.4 34 116.9 71.6 1.37 . . . . . . .

55 14 2.52 610 171 317 . 251 0.952 . . 24.2 33.1 72.3 39.3 0.31 . . . . . . .

56 14 1.95 610 171 314 . 249 0.952 . . 23.7 33.5 100.7 60.5 0.51 . . . . . . .

57 15 1.61 1000 . . . . 1.319 0.947 388 9.8 10.85 36 25.33 0.17 57.33 44 3.33 11.82 31.51 0.620 0.724

58 15 1.39 874 . . . . 0.439 0.415 434 11.9 14.55 43 28 0.22 62.5 40 3.5 12.86 34.27 0.643 0.852

59 15 1.51 1000 . . . . 0.501 0.303 370 10.2 11.85 38.5 29.5 0.2 58.5 48.5 4.3 11.41 30.40 0.525 0.617

60 15 1.31 812 . . . . 1.170 0.270 416 14.1 15.4 50.5 31.5 0.26 72 52.5 4.05 12.45 33.20 0.618 0.676

61 16 6.43 1000 . . . . 0.753 0.606 403 13.53 8.5 47 26.33 0.48 304 212 3.97 12.16 32.42 0.688 .

62 16 6.22 1000 . . . . 0.585 1.778 356 12.7 11.65 45 29 0.48 283.5 206.5 3.85 11.08 29.54 0.591 .

63 16 6.23 945 . . . . 1.000 1.000 448 16.5 13.4 57 23 0.6 360 200 4.2 13.17 35.09 0.817 0.530

64 16 5.86 882 . . . . 1.000 1.000 422 15.3 13.6 54 27 0.57 307 202 3.1 12.59 33.56 0.723 0.441

65 17 2.82 897 . . . . 1.210 1.237 389 12.08 13.15 39.83 24.32 0.35 116.28 85.68 1.07 11.83 31.55 0.678 0.525

66 17 2.68 878 . . . . 2.089 1.129 322 12.33 14.37 42.1 26.43 0.33 111.87 81.17 0.77 10.28 27.40 0.608 0.512

67 17 3.39 889 . . . . 0.491 0.859 395 11.27 8.8 44.7 29.58 1.05 150.15 107.7 2.03 11.97 31.92 0.583 0.482

68 17 3.17 886 . . . . 0.290 0.868 334 13.15 12.5 45.5 35.05 1 146.2 112.3 1.25 10.55 28.13 . 0.416

69 18 3.38 844 . . . . 0.685 0.219 356 12.65 16.25 46.5 31 0.17 162.67 137.17 2.32 11.08 29.54 0.568 .

70 18 4.14 835 . . . . 0.587 0.189 350 12.53 17.8 44.5 26.67 0.25 188.83 146.17 2.22 10.94 29.16 0.641 .

71 18 4.26 865 . . . . 0.547 0.218 359 11.63 14.93 37.83 23.5 0.27 168 125.17 2.87 11.15 29.72 0.666 0.415

72 18 3.29 847 . . . . 0.702 0.217 404 12.73 17.05 46.83 29.5 0.36 162.83 125.33 2.93 12.18 32.48 0.623 0.412

73 19 7.08 825 . . . . 0.351 1.778 365 13 12.15 33.5 21 0.08 240 163 7 11.29 30.10 0.708 .

74 19 6.85 807 . . . . 1.170 1.292 347 13.2 13.05 34 21.5 0.08 244.5 148.5 4.8 10.87 28.98 0.685 0.492

75 19 7.96 885 . . . . 0.585 0.169 357 12.3 11.85 30 19 0.07 235.5 141.5 8 11.09 29.57 0.734 0.491

76 19 7.67 881 . . . . 1.000 1.000 363 13.7 11.7 33 22 0.07 253 160 3.2 11.24 29.97 0.672 0.473

77 20 5.00 1000 . . . . 0.611 0.398 395 12.23 7.3 28.83 19 0.9 147.13 144.8 1.27 11.99 31.95 0.726 .

78 20 3.45 749 . . . . 1.057 0.442 419 14.37 7.2 41.17 24.1 1.47 142.93 130.17 1.43 12.52 33.38 0.683 .

79 21 3.45 749 . . . . 1.057 0.442 419 14.37 7.2 41.17 24.1 1.47 142.93 130.17 1.43 12.52 33.38 0.683 .

80 21 3.31 718 . . . . 0.650 0.651 425 12.77 6.5 33.43 25.07 0.18 110.5 110.07 1.73 12.65 33.72 0.623 .

81 21 3.69 712 . . . . 0.418 0.388 445 12.5 4.5 35.95 22.8 0.2 133.9 96.5 2.15 13.10 34.92 0.725 0.524

82 22 1.56 743 . . . . 0.877 1.292 421 14 11.35 69 34 0.55 109.5 66.5 2.85 12.57 33.50 0.681 0.673

83 22 2.02 699 . . . . 2.089 1.778 383 11.95 10.58 55.25 36.25 0.93 110.25 61.25 1.78 11.69 31.17 0.539 0.711

84 22 1.67 734 . . . . 0.409 1.016 420 13.55 10.28 66.75 35.5 0.5 110.25 62.5 3.13 12.55 33.45 0.649 0.708

85 22 2.00 704 . . . . 0.828 1.778 383 12.15 10.2 55.25 31 0.93 111 48.75 2.1 11.69 31.17 0.634 0.823

86 23 2.46 565 . . . . 0.407 1.221 523 15.16 27.08 63.6 37.8 0.16 149.8 97.6 2.04 14.78 39.39 0.635 0.598

87 23 2.39 567 . . . . 0.975 0.718 538 15.2 24.58 67.4 35.8 0.52 157.4 81.6 2.28 15.10 40.26 0.685 0.723

88 23 2.30 511 . . . . 0.685 0.772 365 10.44 15 47.6 25.8 0.62 110.2 60.4 1.36 11.29 30.10 0.682 0.713

89 23 2.52 536 . . . . 0.432 0.903 316 10.98 15.92 46.2 24.4 0.18 114.2 64.2 1.68 10.13 27.00 0.687 0.660

90 24 1.70 729 . . . . 0.816 1.685 417 12.75 11.9 55.3 29.15 0.15 96.2 62.15 1.5 12.46 33.23 0.696 0.684

91 24 2.08 712 . . . . 2.089 0.321 414 12.5 12.53 48.65 35.55 0.25 97.7 73.1 1.55 12.40 33.05 0.519 0.574

92 24 2.27 726 . . . . 0.311 0.222 419 12.05 10.9 39.65 29.85 0.1 90 72.8 1.1 12.52 33.38 0.560 0.550

93 24 1.67 678 . . . . 0.300 0.868 437 14.9 17.3 66.9 42.35 0.3 110.8 64.9 1.6 12.92 34.45 0.556 0.711

94 24 2.41 673 . . . . 0.217 0.584 445 14.2 15.55 51.8 37.2 0.25 125.8 98.15 1.75 13.10 34.92 0.530 0.483

95 24 2.70 662 . . . . 0.190 1.778 442 13.15 15.1 35.85 26.05 0.15 95 84.1 1.2 13.02 34.71 0.632 0.467

96 25 2.17 673 . . . . 0.312 0.403 464 14.17 15.83 51.2 37.53 0.27 111.87 82.53 1.23 13.52 36.03 0.526 0.573

97 25 2.14 669 . . . . 0.208 1.091 419 14 16.13 51.83 32.87 0.2 109.2 82.23 1.8 12.51 33.36 0.603 0.536

98 26 4.45 839 . . . . 0.877 0.580 384 14.93 17.65 43.5 19.55 0.67 192.95 86.18 0.22 11.72 31.25 0.805 0.714

99 26 4.10 828 . . . . 1.309 1.472 387 13.98 17.9 47.65 13.93 0.34 180.6 84.77 0.13 11.79 31.43 0.948 0.704

100 26 4.00 822 . . . . 0.477 0.766 393 13.45 17.18 45.1 15.63 0.45 182.9 87.08 0.24 11.93 31.80 0.908 0.696

101 26 4.23 828 . . . . 1.426 1.633 388 13.93 17.23 42.13 16.48 0.38 183.47 72.25 0.26 11.81 31.48 0.880 0.776

102 27 5.05 1000 . . . . 1.117 0.438 389 11.39 10.2 23.25 10.99 0.75 122.36 111.43 1.08 11.84 31.56 1.004 .

103 27 5.06 832 . . . . 1.671 0.821 392 13.49 11.46 24.38 15.21 0.46 122.88 144.77 0.85 11.91 31.75 0.846 .

104 28 3.96 1000 . . . . 1.154 0.644 429 12.28 8.8 59.3 45.63 0.07 238.92 176.63 1.25 12.73 33.94 . 0.398

105 28 3.83 823 . . . . 1.154 1.022 429 12.79 8.54 52.5 42.36 0.06 207.75 171.7 1.05 12.73 33.94 . .

106 29 1.53 552 177 326 . . 1.053 1.000 472 16.1 19.5 74.5 54.7 0.77 . . . 13.69 36.50 . .

107 29 1.53 552 177 326 . . 1.053 1.000 472 17.1 20.3 76.7 50.4 2.62 . . . 13.69 36.50 0.487 .

108 29 1.53 552 177 326 . . 1.053 1.000 472 17 20.2 77.8 59.4 0.99 . . . 13.69 36.50 . .

109 29 1.53 552 177 326 . . 1.053 1.000 472 17.2 20.9 74.1 55.3 1.7 . . . 13.69 36.50 . .

110 30 1.75 . 151 331 . . 0.516 0.661 638 . 43.2 89.6 47.6 0.2 175.9 114.8 4 17.16 45.75 0.658 0.585

111 30 1.75 . 151 331 . . 0.516 0.661 638 . 35.4 72.8 39.6 0.2 137.7 89.4 1.8 17.16 45.75 0.689 0.670

112 31 1.50 650 149 337 40 . 0.884 1.892 699 14.3 34 74.4 39.8 0.1 112.1 69.3 1.7 18.39 49.01 0.711 0.804

113 31 1.50 650 149 337 40 . 0.990 2.136 676 14.3 34.9 74 39.5 0.2 112.7 66.7 0.6 17.92 47.77 0.706 0.827

114 32 1.95 588 142 469 24 139 1.415 . 584 16.1 20.7 74.2 56.9 0.5 . . . 16.06 42.81 . .

115 32 1.41 588 135 402 17 558 1.415 . 584 16 20.1 68.2 49.1 0.55 . . . 16.06 42.81 0.508 .

116 32 1.24 588 145 408 20 488 1.415 . 584 16.2 19.4 77.7 57.2 0.51 . . . 16.06 42.81 0.464 .

117 32 2.44 588 145 438 32 24 1.415 . 584 16 19.4 68 49 0.05 . . . 16.06 42.81 0.515 .

118 32 1.79 588 121 401 20 436 1.415 . 584 15.1 16.6 53.6 40.4 0.14 . . . 16.06 42.81 0.543 .

119 32 1.44 588 155 425 22 323 1.415 . 584 16.8 20.3 80.3 59 1.39 . . . 16.06 42.81 0.448 .

120 33 1.74 588 159 392 . 374 1.235 . 620 17.65 23.45 68.15 40.2 0.975 . . . 16.80 44.78 0.642 .

121 33 1.86 588 159 393 . 382 1.235 . 620 17.75 23.25 66.95 40 0.56 . . . 16.80 44.78 0.645 .

122 33 2.12 588 158 401 . 297 1.235 . 620 17.75 23.6 66.7 40.4 0.845 . . . 16.80 44.78 0.633 .

123 33 1.50 588 194 381 . 252 1.235 . 620 17.9 24.15 71.3 42.25 1.235 . . . 16.80 44.78 0.626 .

124 33 1.74 588 193 392 . 273 1.235 . 620 18 23.95 70.1 41.9 0.82 . . . 16.80 44.78 0.630 .

125 33 2.06 588 192 401 . 367 1.235 . 620 18 24.3 69.85 42.3 1.105 . . . 16.80 44.78 0.619 .

126 34 2.58 . 180 349 . . 0.550 . 704 28.3 44.5 128 65.8 . . . . 18.49 49.28 . .

127 34 2.43 . 181 347 . . 0.550 . 700 28.8 43.5 134.1 64 . . . . 18.39 49.02 . .

128 34 2.41 . 182 348 . . 0.550 . 703 29 43.5 134 66.8 . . . . 18.45 49.19 . .

129 34 2.62 . 182 351 . . 0.550 . 706 28.2 43 127.2 60.4 . . . . 18.52 49.36 . .

130 35 1.71 540 163 285 . . 0.798 1.380 574 23.5 36.3 92.5 46.3 0.86 157.6 70.5 0.3812 15.85 42.26 0.662 0.818

131 35 1.82 540 161 306 . . 0.798 1.380 552 20.9 33.3 71.9 40.3 0.74 138.3 62.9 0.5788 15.39 41.04 0.643 0.838

132 35 1.81 540 160 285 . . 0.798 1.380 561 22.5 38.8 80.9 45.8 0.61 157.9 79.4 0.5397 15.59 41.57 0.619 0.757

133 35 1.85 540 159 300 . . 0.798 1.380 556 20.6 32.4 71.9 39 0.54 136.3 61.8 0.8686 15.48 41.27 0.665 0.843

134 36 1.74 449 168 . . . 0.651 . 577 25.5 41.1 117.5 82.4 0.41 . . . 15.92 42.43 . .

135 36 1.74 449 168 . . . 0.651 . 592 26.7 42.9 119.3 83.8 0.34 . . . 16.23 43.25 . .

136 36 1.67 607 148 . . . 0.651 . 634 24.3 32.6 99.6 89.1 0.14 . . . 17.08 45.54 . .

137 36 1.67 607 148 . . . 0.651 . 632 24.3 31.4 98.9 82.3 0.14 . . . 17.04 45.43 . .

138 37 2.26 438 166 264 . . 0.434 1.142 609 23.2 50 78.9 37.1 0.27 178.8 96.5 1.37 16.57 44.18 0.736 0.700

139 37 2.26 438 166 264 . . 0.434 1.142 598 22.4 48.9 70 36.8 0.27 172.5 109.7 0.53 16.35 43.58 0.704 0.614

140 37 2.26 438 166 264 . . 0.434 1.142 606 27.4 50.3 93.1 44.9 0.43 211 124.8 1.3 16.51 44.02 0.690 0.611

141 37 2.26 438 166 264 . . 0.434 1.142 607 26.6 49.9 90.3 46.2 0.33 204.5 130.7 0.84 16.53 44.07 0.668 0.572

142 37 2.26 438 166 264 . . 0.434 1.142 606 26.8 48.6 91 46.3 0.3 206.1 134.5 0.89 16.51 44.02 0.669 0.557

143 37 1.43 436 167 262 . . 0.387 1.019 599 24.4 52.8 110.2 84.8 1.33 156.8 121.1 0.31 16.37 43.64 . 0.504

144 37 1.43 436 167 262 . . 0.387 1.019 564 26.8 51 139.3 86.4 0.58 198.2 115.1 0.86 15.65 41.71 0.488 0.625

145 37 1.43 436 167 262 . . 0.387 1.019 573 27.2 50.1 143.6 99.2 1.31 200.8 132.9 0.35 15.83 42.21 . 0.547

146 37 1.43 436 167 262 . . 0.387 1.019 565 27.8 46.1 144.5 81.5 1.57 205.6 130.4 0.51 15.67 41.77 0.534 0.566

147 37 1.43 436 167 262 . . 0.387 1.019 565 26.6 42.2 138.2 85.5 1.63 196.6 119.4 0.37 15.67 41.77 0.483 0.603

148 37 1.07 434 166 262 . . 0.387 0.988 606 22.1 47.5 148 101.7 6.08 159.8 94.2 0.06 16.51 44.02 . 0.686

149 37 1.07 434 166 262 . . 0.387 0.988 585 22.5 45.4 150.6 99.7 4.18 161.8 98.3 0.41 16.08 42.87 . 0.655

150 37 1.07 434 166 262 . . 0.387 0.988 588 24.3 45.2 163 110.8 3.66 175.2 99.9 0.1 16.14 43.03 . 0.675

151 37 1.07 434 166 262 . . 0.387 0.988 598 24.8 45.6 166.2 111.9 3.33 179.3 118.6 0.24 16.35 43.58 . 0.580

152 37 1.07 434 166 262 . . 0.387 0.988 601 26.8 45.4 179.7 118.8 2.26 193.1 132.7 0.19 16.41 43.75 . 0.538

153 38 2.36 449 174 261 . . 0.555 . . 22.2 38.7 103.9 51.3 5.42 . . . . . . .

154 38 3.09 450 171 261 . . 0.555 . . 21.5 38.8 67.7 38 1.87 . . . . . . .

155 38 2.88 450 174 260 . . 0.555 . . 21.7 41.3 69.5 33.6 1.2 . . . . . . .

156 39 . 600 166 394 . . 1.000 . 614 20.7 29.5 76.1 46.5 1.8 . . . 16.68 44.45 0.584 .

157 40 3.31 552 164 335 . . 2.089 1.778 645 20.6 40 31.8 16.9 0.06 105 95.9 4.3 17.30 46.13 1.011 0.485

158 40 2.44 656 161 314 . . 2.089 1.778 645 22.7 40 48 21.8 0.1 117 90.3 5.1 17.30 46.13 0.904 0.579

159 41 . . . . . . 1.000 . 604 . 21.62 59.11 41.82 0.239 95.49 72.397 0.688 16.48 43.92 0.567 0.695

160 42 . . . . . . 1.000 . 603 . 20.5 79.536 57.48 0.294 57.187 44.6 0.96 16.45 43.85 0.480 0.970

161 43 . . . . . . 1.000 . 597 . 20.71 63.02 50.46 0.231 46.58 31.68 1.484 16.34 43.55 . .

162 44 . . . . . . 1.000 . 586 . 19.69 38.799 29.05 0.25 78.598 62.196 1.519 16.10 42.93 0.660 0.736

163 45 . . . . . . 1.000 . 489 . 14.1 51.78 31.69 0.513 72.08 54.58 1.52 14.06 37.47 0.650 0.742

164 46 3.90 375 161 359 . . 1.000 . 590 . . 60 53.74 0.52 . . . 16.19 43.14 . .

165 46 3.90 375 161 359 . . 1.000 . 590 . . 60 32.39 0.87 . . . 16.19 43.14 0.715 .

166 46 3.90 375 161 359 . . 1.000 . 590 . . 60 33.57 0.61 . . . 16.19 43.14 0.700 .

167 46 3.90 375 161 359 . . 1.000 . 590 . . 60 41.57 0.63 . . . 16.19 43.14 0.566 .

168 46 3.54 375 154 360 . . 1.000 . 590 . . 82 44.32 0.75 . . . 16.19 43.14 0.648 .

169 46 3.54 375 154 360 . . 1.000 . 590 . . 82 41.51 1.38 . . . 16.19 43.14 0.674 .

170 46 3.54 375 154 360 . . 1.000 . 590 . . 82 45.48 1.36 . . . 16.19 43.14 0.626 .

171 46 3.54 375 154 360 . . 1.000 . 590 . . 82 49.09 1.54 . . . 16.19 43.14 0.580 .

172 46 3.05 375 154 364 . . 1.000 . 590 . . 112 50.83 0.79 . . . 16.19 43.14 0.684 .

173 46 3.05 375 154 364 . . 1.000 . 590 . . 112 59.01 3.37 . . . 16.19 43.14 0.588 .

174 46 3.05 375 154 364 . . 1.000 . 590 . . 112 66.4 4.91 . . . 16.19 43.14 0.508 .

175 46 3.05 375 154 364 . . 1.000 . 590 . . 112 75.53 4.36 . . . 16.19 43.14 . .

176 47 3.62 557 203 390 . . 2.134 . 640 11.3 15.7 29.4 15.3 0.26 . . . 17.20 45.86 1.056 .

177 47 3.62 557 203 390 . . 2.134 . 530 15.3 23.9 39.8 18.2 0.07 . . . 14.93 39.81 0.916 .

178 47 3.62 557 203 390 . . 2.134 . 603 25.1 47 65.2 26.35 0.06 . . . 16.45 43.86 0.847 .

179 48 2.26 550 165 340 40 . 2.134 . 632 20.8 36.4 74.2 . . . . . 17.05 45.44 . .

180 48 1.88 550 164 335 42 . 2.134 . 664 22 35.4 92.3 . . . . . 17.69 47.16 . .

181 49 . . . . . . 0.848 . . . . 81.4 47.5 0.07 . . . . . . .

182 49 . . . . . . 0.848 . . . . 87.3 52.6 0.09 . . . . . . .

183 50 . 480 190 360 . . 0.691 . 754 . 37 104.8 63.4 . . . . 19.45 51.86 0.581 .

184 50 . 515 192 391 . . 0.691 . 754 . 37 110.9 77.4 . . . . 19.45 51.86 0.477 .

185 51 . 508 137 314 29 . 1.768 . 627 23.8 27.9 58.5 42 0.038 . . . 16.94 45.16 0.571 .

186 51 . 508 144 311 30 . 1.768 . 627 21.6 25.6 53.1 35.7 0.033 . . . 16.94 45.16 0.646 .

187 51 . 511 133 313 30 . 1.768 . 627 21 24.7 51.9 37.1 0.034 . . . 16.94 45.16 0.611 .

188 52 3.00 700 140 302 35 . 1.431 . 597 19.8 30.6 47.9 21.6 0.22 . . . 16.33 43.53 0.885 .

189 52 2.03 700 148 295 35 . 1.431 . 597 19.6 30.8 67.2 30.8 0.26 . . . 16.33 43.53 0.781 .

190 53 2.42 555 182 347 . . 0.750 . 463 17.9 33 78.3 41.5 0.2 . . . 13.49 35.97 0.640 .

191 53 2.23 549 176 319 . . 0.750 . 463 18.6 32.6 84.2 58.2 0.26 . . . 13.49 35.97 0.466 .

192 53 1.67 545 186 350 . . 0.750 . 463 18.1 32.8 98.8 64.6 0.31 . . . 13.49 35.97 0.480 .

193 53 1.70 552 170 361 . . 0.750 . 463 17.8 34 93.3 50.5 0.61 . . . 13.49 35.97 0.597 .

194 54 . 765 . . . . 2.230 . . 16.1 27.4 53.1 23.8 0.6 . . . . . . .

195 54 . 765 . . . . 2.230 . . 17.1 26.9 48.2 22 0.9 . . . . . . .

196 54 . 765 . . . . 2.230 . . 18 26.1 48.7 21.7 0.6 . . . . . . .

197 54 . 765 . . . . 2.230 . . 17.5 27.1 33.1 16.2 0.5 . . . . . . .

198 55 1.07 258 . . . . . . . 15.5 14.86 87 41.8 0.8 93.2 47.4 0.6 . . . .

199 55 1.18 258 . . . . . . . 15 16.98 72.4 47.7 0.3 85.4 53.2 0.6 . . . .

200 55 1.45 258 . . . . . . . 15.1 17.32 63.6 51.5 1 92.2 66.6 0.7 . . . .

201 56 2.07 500 186 415 . . 1.000 . 545 18.4 19.5 97.2 28.4 . . . . 15.25 40.65 . .

202 56 1.85 411 207 457 . . 1.000 . 545 15.8 14.5 95.2 25.6 . . . . 15.25 40.65 . .

203 56 1.96 417 196 431 . . 1.000 . 545 16.8 25.9 96.1 29.4 . . . . 15.25 40.65 . .

204 57 2.67 367 171 401 . . 0.763 . 678 18.6 34.1 69.2 49.1 . . . . 17.96 47.89 . .

205 57 4.00 365 171 403 . . 0.763 . 678 18 33 50.9 25.7 . . . . 17.96 47.89 . .

206 58 3.05 419 162 . 19 . 2.089 . 603 16.9 20.8 37 20.3 0.3 . . . 16.45 43.86 0.888 .

207 58 3.56 419 162 . 19 . 2.089 . 597 10.9 10 21.5 13.3 0.3 . . . 16.33 43.53 . .

208 59 1.22 . . . . . 0.923 1.778 547 14.5 . 58.9 13.9 1.31 71.8 25.4 3.8 15.29 40.76 1.001 .

209 59 1.20 . . . . . 0.923 1.778 543 14.3 . 56.2 13.9 1.54 67.2 24.7 5.1 15.21 40.54 0.996 .

210 59 1.11 . . . . . 0.923 1.778 548 15.4 . 63 18 1.2 70.1 27.4 2.6 15.31 40.82 0.938 .

211 59 1.08 . . . . . 0.923 1.778 547 16.5 . 75.9 21.9 1 82.1 30 6.1 15.29 40.76 0.900 1.057

212 59 1.04 . . . . . 0.923 1.778 542 14.9 . 61.4 14.5 1.15 63.7 21.2 2.9 15.19 40.48 0.992 .

213 60 1.50 . . . . . 1.949 1.778 545 15.1 . 67.2 22.8 0.2 100.6 37.2 5.8 15.25 40.65 0.885 0.977

214 60 1.58 . . . . . 1.949 1.778 544 16.3 . 64.4 21.2 0.24 102 39.8 7.2 15.23 40.60 0.904 0.937

215 60 1.24 . . . . . 1.949 1.778 545 14.9 . 62.4 20.8 0.18 77.2 31.2 8.6 15.25 40.65 0.908 1.011

216 60 1.06 . . . . . 1.949 1.778 560 16.6 . 68.8 27.2 0.28 72.6 33.6 6.4 15.56 41.49 0.827 1.021

217 60 0.89 . . . . . 1.949 1.778 566 14.7 . 66.4 18.2 0.34 58.8 23.8 7.7 15.69 41.82 0.957 .

218 61 1.53 475 169 316 . . 0.783 0.742 . 24.1 32.4 76.1 50.86 0.816 122.54 87.66 0.718 . . . .

219 61 2.29 475 169 309 . . 0.620 0.538 . 23.4 34.2 72.88 45.24 1.402 184.58 129.62 0.598 . . . .

220 61 3.03 475 168 301 . . 0.726 0.701 . 22.7 31.9 71.24 46.46 0.856 249.7 174.56 0.826 . . . .

221 62 . 780 . . . . 1.032 . . 22.7 . 79.9 43.7 . . . . . . . .

222 62 . 820 . . . . 1.032 . . 21.3 . 59.5 29.1 . . . . . . . .

223 62 . 820 . . . . 1.032 . . 22.1 . 51.9 26.5 . . . . . . . .

224 63 . 810 . . . . 0.650 . . 22.5 . 88 39.2 . . . . . . . .

225 63 . 700 . . . . 0.650 . . 24.6 . 70.9 28.1 . . . . . . . .

226 63 . 840 . . . . 0.650 . . 22.2 . 57.2 21.1 . . . . . . . .

227 64 . 700 258 265 28 . 2.230 . 558 13.6 20.5 45 22 2.4 . . . 15.52 41.38 0.803 .

228 64 . 700 254 269 27 . 2.063 . 558 13.3 20.8 34 19 0.2 . . . 15.52 41.38 0.892 .

229 64 . 700 256 280 26 . 2.063 . 558 13.6 20.5 34 18 0.1 . . . 15.52 41.38 0.924 .

230 65 . 500 174 296 . . . . 699 25 42.4 77.5 43 . . . . 18.38 48.99 . .

231 65 . 500 174 295 . . . . 671 25 38.7 97.5 66 . . . . 17.81 47.49 . .

232 65 . 500 173 293 . . . . 655 24.6 39.4 115.6 88 . . . . 17.50 46.66 . .

233 66 2.10 500 178 314 . . 0.604 . 661 23 35 58.7 31.8 . . . . 17.61 46.96 . .

234 66 2.10 500 178 314 . . 0.563 . 661 23 35 68.4 39.4 . . . . 17.61 46.96 . .

235 66 2.10 500 178 314 . . 0.434 . 661 23 35 72.8 39.9 . . . . 17.61 46.96 . .

236 66 2.10 500 178 314 . . 0.619 . 661 23 35 73.6 37.1 . . . . 17.61 46.96 . .

237 66 2.10 500 178 314 . . 0.798 . 661 23 35 81.6 44.5 . . . . 17.61 46.96 . .

238 66 2.10 500 178 314 . . 0.728 . 661 23 35 59.9 30.2 . . . . 17.61 46.96 . .

239 66 1.63 500 178 312 . . 0.604 . 679 22.4 36.5 68 36.4 . . . . 17.97 47.91 . .

240 66 1.63 500 178 312 . . 0.563 . 679 22.4 36.5 82.6 47.9 . . . . 17.97 47.91 . .

241 66 1.63 500 178 312 . . 0.434 . 679 22.4 36.5 90.4 51.3 . . . . 17.97 47.91 . .

242 66 1.63 500 178 312 . . 0.619 . 679 22.4 36.5 93.1 48.9 . . . . 17.97 47.91 . .

243 66 1.63 500 178 312 . . 0.798 . 679 22.4 36.5 104.4 68.8 . . . . 17.97 47.91 . .

244 66 1.63 500 178 312 . . 0.728 . 679 22.4 36.5 85.7 61.5 . . . . 17.97 47.91 . .

245 66 1.33 500 178 310 . . 0.604 . 635 23.4 36.2 83.9 44.6 . . . . 17.10 45.59 . .

246 66 1.33 500 178 310 . . 0.563 . 635 23.4 36.2 103.1 54 . . . . 17.10 45.59 . .

247 66 1.33 500 178 310 . . 0.434 . 635 23.4 36.2 111.8 68.9 . . . . 17.10 45.59 . .

248 66 1.33 500 178 310 . . 0.619 . 635 23.4 36.2 117 60.7 . . . . 17.10 45.59 . .

249 66 1.33 500 178 310 . . 0.798 . 635 23.4 36.2 134.4 80 . . . . 17.10 45.59 . .

250 66 1.33 500 178 310 . . 0.728 . 635 23.4 36.2 116.2 97.3 . . . . 17.10 45.59 . .

251 67 2.14 370 183 354 . . 0.934 . 596 25.9 37.8 108.9 66.9 . . . . 16.30 43.46 . .

252 67 2.15 370 183 354 . . 0.934 . 601 25.1 38.8 105.3 55.2 . . . . 16.42 43.76 . .

253 68 1.34 525 174 291 . . 0.791 0.999 . 21 44.4 99.2 58 . 131.3 100.6 . . . . .

254 68 1.76 527 175 292 . . 0.791 0.999 . 20.7 44.5 78.3 40.8 . 133.8 89.2 . . . . .

255 68 0.98 528 175 292 . . 0.791 0.999 . 21.9 44 106.5 64.2 . 104.3 70.2 . . . . .

256 68 1.18 528 175 292 . . 0.791 0.999 . 21.4 42.9 79.2 43.9 . 95.8 75.4 . . . . .

257 69 . 607 158 294 . . 0.728 . 702 25.8 42.5 87.4 57.5 . . . . 18.43 49.13 . .

258 69 . 549 158 331 . . 0.728 . 641 24.2 41.7 81.4 45.6 . . . . 17.22 45.91 . .

259 69 . 602 157 292 . . 0.728 . 659 26.9 43.4 127.3 96.8 . . . . 17.57 46.85 . .

260 69 . 544 157 330 . . 0.728 . 673 26.1 44.5 122.8 82.4 . . . . 17.86 47.62 . .

261 70 . 480 180 271 . . 0.619 . 706 23.1 36.4 73.8 45.2 . . . . 18.52 49.36 . .

262 70 . 580 182 303 . . 0.619 . 720 24 33.8 74.8 52.2 . . . . 18.79 50.09 . .

263 70 . 480 180 271 . . 0.619 . 696 23.2 36.5 95.8 63.7 . . . . 18.31 48.81 . .

264 70 . 580 181 303 . . 0.619 . 702 23.9 34.2 95.7 66 . . . . 18.43 49.13 . .

265 71 1.66 520 161 354 35 . 1.238 0.662 603 22.5 35.9 82 35 . 139 99 . 16.45 43.86 . .

266 71 2.26 520 160 347 34 . 1.238 0.662 594 21.8 34 83 31 . 189 153 . 16.27 43.36 . .

267 71 1.64 520 161 355 35 . 1.238 0.662 588 21.7 34.5 84 35 . 137 85 . 16.14 43.03 . .

268 71 2.57 520 157 344 34 . 1.238 0.662 600 21.9 34.9 82 34 . 210 147 . 16.39 43.69 . .

269 71 1.71 520 158 352 35 . 1.238 0.662 596 22.6 37.6 88 36 . 149 95 . 16.31 43.47 . .

270 71 2.57 520 158 355 34 . 1.238 0.662 592 20.4 33.2 77 29 . 199 155 . 16.23 43.25 . .

271 71 1.71 520 159 354 35 . 1.238 0.662 588 21 33.7 78 32 . 134 92 . 16.14 43.03 . .

272 71 2.78 520 161 352 34 . 1.238 0.662 594 20.7 33.2 75 29 . 207 142 . 16.27 43.36 . .

273 72 . 497 150 370 22 . 0.890 . . 22.8 32.7 95.2 60.1 . . . . . . . .

274 72 . 496 147 371 23 . 0.890 . . 22.7 31.7 116 85.9 . . . . . . . .

275 72 . 496 151 378 42 . 0.890 . . 23.3 30.8 122 91.7 . . . . . . . .

276 72 . 496 156 379 42 . 0.890 . . 23.2 29.6 117 88.1 . . . . . . . .

277 73 2.97 506 151 364 . . 1.000 . 556 18.4 28.9 62.3 37.3 0.38 . . . 15.48 . 0.644 .

278 73 2.54 506 150 365 . . 1.000 . 556 18.4 29.5 72.1 39.4 0.45 . . . 15.48 . 0.662 .

279 73 2.27 506 150 363 . . 1.000 . 556 18.4 29.5 81.8 44.7 0.44 . . . 15.48 . 0.637 .

280 73 2.30 506 172 366 . . 1.000 . 556 18.8 29.8 83.4 45.2 0.51 . . . 15.48 . 0.638 .

281 74 1.73 675 141 359 26 245 0.952 . 688 17 25 64.3 57.7 0.19 . . . 18.16 . . .

282 74 1.59 675 139 346 28 251 0.707 . 715 17 21 64 50.2 0.5 . . . 18.69 . 0.500 .

283 75 2.20 600 160 318 25 220 1.125 . . 23.9 35.3 77.6 40.7 . . . . . . . .

284 75 2.21 600 160 321 25 214 1.125 . . 24 34.2 80.4 39.1 . . . . . . . .

285 75 1.71 600 174 351 26 175 1.125 . . 23.6 35.4 89.9 48.6 . . . . . . . .

286 75 1.78 600 174 348 25 167 1.125 . . 23.9 33.3 88.1 45.3 . . . . . . . .

287 75 1.97 600 180 285 25 247 1.125 . . 22.6 34 79.6 39.3 . . . . . . . .

288 75 2.00 600 180 284 26 251 1.125 . . 22.6 36.3 81.6 37.6 . . . . . . . .

289 75 2.20 600 160 318 25 220 0.884 . . 23 23.5 74.7 43.3 . . . . . . . .

290 75 2.21 600 160 321 25 214 0.884 . . 22.8 23.6 75.4 43.2 . . . . . . . .

291 75 1.71 600 174 351 26 175 0.884 . . 22.7 23.3 87.3 55.9 . . . . . . . .

292 75 1.78 600 174 348 25 167 0.884 . . 22.6 23.7 83.1 57.1 . . . . . . . .

293 75 1.97 600 180 285 25 247 0.884 . . 22.3 22.3 78.4 43.3 . . . . . . . .

294 75 2.00 600 180 284 26 251 0.884 . . 22.2 23.5 80 42.3 . . . . . . . .

295 76 1.81 565 . . . . 0.994 1.000 469 11.46 10.04 35.84 31.76 1.51 64.76 2.28 61.89 13.63 36.32 0.452 0.570

296 76 1.62 576 . . . . 0.994 1.000 476 12.81 12.74 52.49 41.59 2.2 85.29 2.58 72.46 13.78 36.73 . 0.551

297 76 1.80 647 . . . . 0.994 1.000 480 14.59 12.87 59.98 43.83 2.35 107.81 2.67 73.41 13.86 36.96 0.461 0.637

298 76 1.65 601 . . . . 0.994 1.000 485 15.68 14.08 73.14 47.12 2.71 120.51 2.74 77.77 13.97 37.25 0.510 0.641

;

proc means;

Title Means overall;

var Ca_P_Ratio Foragediet CPdiet NDFdiet EEdiet StarchDiet InverseTruncSEMPfecalPolled InverseTruncSEMCafecalPolled BW DMI MY Pintake Pfecal Purinary Caintake Cafecal Caurinary Est_End_P Est_End_Ca MetabolazibleP MetabolazibleCa ;

run;

proc means;

proc corr;

var Ca_P_Ratio Foragediet CPdiet NDFdiet EEdiet StarchDiet BW DMI MY Pintake Pfecal Purinary Caintake Cafecal Caurinary Est_End_P Est_End_Ca MetabolazibleP MetabolazibleCa ;

run;

PROC MIXED COVTEST;

Class Study;

MODEL MetabolazibleP = Foragediet/solution;

RANDOM intercept/Type=un Subject=Study;

weight InverseTruncSEMPfecalPolled;

RUN;

PROC MIXED COVTEST;

Class Study;

MODEL MetabolazibleP = NDFdiet/solution;

RANDOM intercept/Type=un Subject=Study;

weight InverseTruncSEMPfecalPolled;

RUN;

PROC MIXED COVTEST;

Class Study;

MODEL MetabolazibleP = EEdiet/solution;

RANDOM intercept/Type=un Subject=Study;

weight InverseTruncSEMPfecalPolled;

RUN;

PROC MIXED COVTEST;

Class Study;

MODEL MetabolazibleP = CPdiet/solution;

RANDOM intercept/Type=un Subject=Study;

weight InverseTruncSEMPfecalPolled;

RUN;

PROC MIXED COVTEST;

Class Study;

MODEL MetabolazibleP = starchdiet/solution;

RANDOM intercept/Type=un Subject=Study;

weight InverseTruncSEMPfecalPolled;

RUN;

PROC MIXED COVTEST;

Class Study;

MODEL MetabolazibleCa = NDFdiet/solution;

RANDOM intercept/Type=un Subject=Study;

WEIGHT InverseTruncSEMCafecalPolled;

RUN;

PROC MIXED COVTEST;

Class Study;

MODEL MetabolazibleCa = Starchdiet/solution;

RANDOM intercept/Type=un Subject=Study;

WEIGHT InverseTruncSEMCafecalPolled;

RUN;

PROC MIXED COVTEST;

Class Study;

MODEL MetabolazibleCa = EEdiet/solution;

RANDOM intercept/Type=un Subject=Study;

WEIGHT InverseTruncSEMCafecalPolled;

RUN;

PROC MIXED COVTEST;

Class Study;

MODEL MetabolazibleCa = CPdiet/solution;

RANDOM intercept/Type=un Subject=Study;

WEIGHT InverseTruncSEMCafecalPolled;

RUN;

PROC MIXED COVTEST;

Class Study;

MODEL MetabolazibleCa = Foragediet/solution;

RANDOM intercept/Type=un Subject=Study;

WEIGHT InverseTruncSEMCafecalPolled;

RUN;

Means overall 13:45 March 13, 2024 57

The MEANS Procedure

Variable N Mean Std Dev Minimum Maximum

ƒƒƒƒƒƒƒƒƒƒƒƒƒƒƒƒƒƒƒƒƒƒƒƒƒƒƒƒƒƒƒƒƒƒƒƒƒƒƒƒƒƒƒƒƒƒƒƒƒƒƒƒƒƒƒƒƒƒƒƒƒƒƒƒƒƒƒƒƒƒƒƒƒƒƒƒƒƒƒƒƒƒƒƒƒƒƒƒƒƒƒƒƒƒƒƒƒƒƒ

Ca_P_Ratio 235 2.3660851 1.2111162 0.8900000 7.9600000

Foragediet 254 586.4055118 154.9985239 258.0000000 1000.00

CPdiet 196 168.3673469 17.8752875 121.0000000 258.0000000

NDFdiet 190 338.3894737 48.5173388 260.0000000 496.0000000

EEdiet 64 32.3281250 8.2057949 17.0000000 47.0000000

StarchDiet 44 227.1818182 110.2619023 24.0000000 558.0000000

InverseTruncSEMPfecalPolled 289 1.0072595 0.4886909 0.1900000 2.2300000

InverseTruncSEMCafecalPolled 113 0.9981150 0.4790715 0.1690000 2.1360000

BW 237 564.1518987 100.2266612 316.0000000 754.0000000

DMI 258 19.1980620 4.7992884 8.7000000 29.0000000

MY 255 28.2808235 11.0151330 4.5000000 52.8000000

Pintake 298 73.7451174 27.6630249 21.5000000 179.7000000

Pfecal 296 43.2471622 19.7846551 10.9900000 118.8000000

Purinary 190 0.8188421 1.0416126 0.0221000 6.0800000

Caintake 120 142.3189583 59.9221847 46.5800000 360.0000000

Cafecal 120 91.6811083 45.7020170 2.2800000 212.0000000

Caurinary 108 4.6152898 13.3396285 0.0600000 77.7700000

Est_End_P 235 15.5910638 2.1364683 10.1300000 19.4500000

Est_End_Ca 230 41.5128696 5.7156769 27.0000000 51.8600000

MetabolazibleP 157 0.6849236 0.1387460 0.4480000 1.0560000

MetabolazibleCa 81 0.6468148 0.1487669 0.3980000 1.0570000

ƒƒƒƒƒƒƒƒƒƒƒƒƒƒƒƒƒƒƒƒƒƒƒƒƒƒƒƒƒƒƒƒƒƒƒƒƒƒƒƒƒƒƒƒƒƒƒƒƒƒƒƒƒƒƒƒƒƒƒƒƒƒƒƒƒƒƒƒƒƒƒƒƒƒƒƒƒƒƒƒƒƒƒƒƒƒƒƒƒƒƒƒƒƒƒƒƒƒƒ

Means overall 13:45 Saturday, March 13, 2024 58

The MEANS Procedure

Variable N Mean Std Dev Minimum Maximum

ƒƒƒƒƒƒƒƒƒƒƒƒƒƒƒƒƒƒƒƒƒƒƒƒƒƒƒƒƒƒƒƒƒƒƒƒƒƒƒƒƒƒƒƒƒƒƒƒƒƒƒƒƒƒƒƒƒƒƒƒƒƒƒƒƒƒƒƒƒƒƒƒƒƒƒƒƒƒƒƒƒƒƒƒƒƒƒƒƒƒƒƒƒƒƒƒƒƒƒ

Obs 298 149.5000000 86.1694068 1.0000000 298.0000000

Study 298 40.0536913 23.1777745 1.0000000 76.0000000

Ca_P_Ratio 235 2.3660851 1.2111162 0.8900000 7.9600000

Foragediet 254 586.4055118 154.9985239 258.0000000 1000.00

CPdiet 196 168.3673469 17.8752875 121.0000000 258.0000000

NDFdiet 190 338.3894737 48.5173388 260.0000000 496.0000000

EEdiet 64 32.3281250 8.2057949 17.0000000 47.0000000

StarchDiet 44 227.1818182 110.2619023 24.0000000 558.0000000

InverseTruncSEMPfecalPolled 289 1.0072595 0.4886909 0.1900000 2.2300000

InverseTruncSEMCafecalPolled 113 0.9981150 0.4790715 0.1690000 2.1360000

BW 237 564.1518987 100.2266612 316.0000000 754.0000000

DMI 258 19.1980620 4.7992884 8.7000000 29.0000000

MY 255 28.2808235 11.0151330 4.5000000 52.8000000

Pintake 298 73.7451174 27.6630249 21.5000000 179.7000000

Pfecal 296 43.2471622 19.7846551 10.9900000 118.8000000

Purinary 190 0.8188421 1.0416126 0.0221000 6.0800000

Caintake 120 142.3189583 59.9221847 46.5800000 360.0000000

Cafecal 120 91.6811083 45.7020170 2.2800000 212.0000000

Caurinary 108 4.6152898 13.3396285 0.0600000 77.7700000

Est_End_P 235 15.5910638 2.1364683 10.1300000 19.4500000

Est_End_Ca 230 41.5128696 5.7156769 27.0000000 51.8600000

MetabolazibleP 157 0.6849236 0.1387460 0.4480000 1.0560000

MetabolazibleCa 81 0.6468148 0.1487669 0.3980000 1.0570000

ƒƒƒƒƒƒƒƒƒƒƒƒƒƒƒƒƒƒƒƒƒƒƒƒƒƒƒƒƒƒƒƒƒƒƒƒƒƒƒƒƒƒƒƒƒƒƒƒƒƒƒƒƒƒƒƒƒƒƒƒƒƒƒƒƒƒƒƒƒƒƒƒƒƒƒƒƒƒƒƒƒƒƒƒƒƒƒƒƒƒƒƒƒƒƒƒƒƒƒ

Means overall 13:45 , March 13, 2024 59

The CORR Procedure

19 Variables: Ca_P_Ratio Foragediet CPdiet NDFdiet EEdiet

StarchDiet BW DMI MY Pintake

Pfecal Purinary Caintake Cafecal Caurinary

Est_End_P Est_End_Ca MetabolazibleP MetabolazibleCa

Simple Statistics

Variable N Mean Std Dev Sum Minimum Maximum

Ca_P_Ratio 235 2.36609 1.21112 556.03000 0.89000 7.96000

Foragediet 254 586.40551 154.99852 148947 258.00000 1000

CPdiet 196 168.36735 17.87529 33000 121.00000 258.00000

NDFdiet 190 338.38947 48.51734 64294 260.00000 496.00000

EEdiet 64 32.32813 8.20579 2069 17.00000 47.00000

StarchDiet 44 227.18182 110.26190 9996 24.00000 558.00000

BW 237 564.15190 100.22666 133704 316.00000 754.00000

DMI 258 19.19806 4.79929 4953 8.70000 29.00000

MY 255 28.28082 11.01513 7212 4.50000 52.80000

Pintake 298 73.74512 27.66302 21976 21.50000 179.70000

Pfecal 296 43.24716 19.78466 12801 10.99000 118.80000

Purinary 190 0.81884 1.04161 155.58000 0.02210 6.08000

Caintake 120 142.31896 59.92218 17078 46.58000 360.00000

Cafecal 120 91.68111 45.70202 11002 2.28000 212.00000

Caurinary 108 4.61529 13.33963 498.45130 0.06000 77.77000

Est_End_P 235 15.59106 2.13647 3664 10.13000 19.45000

Est_End_Ca 230 41.51287 5.71568 9548 27.00000 51.86000

MetabolazibleP 157 0.68492 0.13875 107.53300 0.44800 1.05600

MetabolazibleCa 81 0.64681 0.14877 52.39200 0.39800 1.05700

Pearson Correlation Coefficients

Prob > |r| under H0: Rho=0

Number of Observations

Ca_P_ Starch

Ratio Foragediet CPdiet NDFdiet EEdiet Diet BW

Ca_P_Ratio 1.00000 0.45349 0.01518 0.21685 0.11504 -0.20671 -0.45508

<.0001 0.8447 0.0054 0.4075 0.1782 <.0001

235 215 169 163 54 44 202

Foragediet 0.45349 1.00000 0.04129 0.09234 -0.32241 0.43206 -0.67028

<.0001 0.5757 0.2176 0.0120 0.0034 <.0001

215 254 186 180 60 44 204

CPdiet 0.01518 0.04129 1.00000 -0.21564 0.07771 -0.14722 -0.06894

0.8447 0.5757 0.0028 0.5416 0.3403 0.3879

169 186 196 190 64 44 159

NDFdiet 0.21685 0.09234 -0.21564 1.00000 0.06443 -0.21738 -0.02926

0.0054 0.2176 0.0028 0.6188 0.1564 0.7195

163 180 190 190 62 44 153

EEdiet 0.11504 -0.32241 0.07771 0.06443 1.00000 -0.47619 0.35172

0.4075 0.0120 0.5416 0.6188 0.0044 0.0142

54 60 64 62 64 34 48

StarchDiet -0.20671 0.43206 -0.14722 -0.21738 -0.47619 1.00000 -0.14505

0.1782 0.0034 0.3403 0.1564 0.0044 0.4615

44 44 44 44 34 44 28

BW -0.45508 -0.67028 -0.06894 -0.02926 0.35172 -0.14505 1.00000

<.0001 <.0001 0.3879 0.7195 0.0142 0.4615

202 204 159 153 48 28 237

DMI -0.33812 -0.57766 -0.01459 -0.59081 0.18553 0.03079 0.69320

<.0001 <.0001 0.8476 <.0001 0.1558 0.8427 <.0001

217 240 176 170 60 44 208

MY -0.43419 -0.63036 0.00449 -0.60180 0.57764 -0.22753 0.71611

<.0001 <.0001 0.9523 <.0001 <.0001 0.1374 <.0001

209 236 180 174 60 44 211

Means overall 13:45 Saturday, March 13, 2024 60

The CORR Procedure

Pearson Correlation Coefficients

Prob > |r| under H0: Rho=0

Number of Observations

Ca_P_ Starch

Ratio Foragediet CPdiet NDFdiet EEdiet Diet BW

Pintake -0.48738 -0.56693 -0.01224 -0.36295 0.24379 0.15081 0.47915

<.0001 <.0001 0.8648 <.0001 0.0522 0.3285 <.0001

235 254 196 190 64 44 237

Pfecal -0.44090 -0.46622 -0.12211 -0.30841 0.06701 0.24339 0.41093

<.0001 <.0001 0.0899 <.0001 0.6048 0.1114 <.0001

233 252 194 188 62 44 235

Purinary -0.22277 -0.33068 0.06257 -0.22108 -0.21021 0.15184 0.16428

0.0052 <.0001 0.5363 0.0322 0.3737 0.5475 0.0385

156 158 100 94 20 18 159

Caintake 0.66274 0.13730 0.07967 -0.25836 -0.77305 . -0.09401

<.0001 0.1731 0.5987 0.0830 0.0087 . 0.3354

112 100 46 46 10 0 107

Cafecal 0.63973 0.23520 0.09737 -0.05009 -0.77493 . -0.13146

<.0001 0.0185 0.5197 0.7410 0.0085 . 0.1771

112 100 46 46 10 0 107

Caurinary -0.09075 -0.02443 -0.25256 0.37142 . . -0.04023

0.3692 0.8212 0.1496 0.0306 . . 0.6926

100 88 34 34 2 0 99

Est_End_P -0.46071 -0.70779 -0.07592 -0.02470 0.35420 -0.14466 0.99968

<.0001 <.0001 0.3447 0.7634 0.0135 0.4627 <.0001

200 202 157 151 48 28 235

Est_End_Ca -0.45790 -0.69320 -0.06492 -0.02103 0.49644 -0.20624 0.99970

<.0001 <.0001 0.4253 0.8004 0.0004 0.3121 <.0001

196 198 153 147 46 26 230

MetabolazibleP 0.20859 0.15322 0.33736 -0.06019 0.01604 -0.51258 0.02826

0.0156 0.0830 0.0017 0.5888 0.9271 0.0074 0.7253

134 129 84 83 35 26 157

MetabolazibleCa -0.39797 -0.20123 -0.43971 0.02474 . . 0.24054

0.0003 0.0948 0.0133 0.8949 . . 0.0305

77 70 31 31 2 0 81

Pearson Correlation Coefficients

Prob > |r| under H0: Rho=0

Number of Observations

DMI MY Pintake Pfecal Purinary Caintake Cafecal

Ca_P_Ratio -0.33812 -0.43419 -0.48738 -0.44090 -0.22277 0.66274 0.63973

<.0001 <.0001 <.0001 <.0001 0.0052 <.0001 <.0001

217 209 235 233 156 112 112

Foragediet -0.57766 -0.63036 -0.56693 -0.46622 -0.33068 0.13730 0.23520

<.0001 <.0001 <.0001 <.0001 <.0001 0.1731 0.0185

240 236 254 252 158 100 100

CPdiet -0.01459 0.00449 -0.01224 -0.12211 0.06257 0.07967 0.09737

0.8476 0.9523 0.8648 0.0899 0.5363 0.5987 0.5197

176 180 196 194 100 46 46

NDFdiet -0.59081 -0.60180 -0.36295 -0.30841 -0.22108 -0.25836 -0.05009

<.0001 <.0001 <.0001 <.0001 0.0322 0.0830 0.7410

170 174 190 188 94 46 46

EEdiet 0.18553 0.57764 0.24379 0.06701 -0.21021 -0.77305 -0.77493

0.1558 <.0001 0.0522 0.6048 0.3737 0.0087 0.0085

60 60 64 62 20 10 10

Means overall 13:45 Saturday, March 13, 2024 61

The CORR Procedure

Pearson Correlation Coefficients

Prob > |r| under H0: Rho=0

Number of Observations

DMI MY Pintake Pfecal Purinary Caintake Cafecal

StarchDiet 0.03079 -0.22753 0.15081 0.24339 0.15184 . .

0.8427 0.1374 0.3285 0.1114 0.5475 . .

44 44 44 44 18 0 0

BW 0.69320 0.71611 0.47915 0.41093 0.16428 -0.09401 -0.13146

<.0001 <.0001 <.0001 <.0001 0.0385 0.3354 0.1771

208 211 237 235 159 107 107

DMI 1.00000 0.86517 0.77236 0.62847 0.25405 0.28243 0.21877

<.0001 <.0001 <.0001 0.0014 0.0028 0.0217

258 242 258 256 156 110 110

MY 0.86517 1.00000 0.71824 0.57253 0.25729 0.16195 0.09847

<.0001 <.0001 <.0001 0.0011 0.0956 0.3129

242 255 255 253 157 107 107

Pintake 0.77236 0.71824 1.00000 0.88230 0.43404 0.19019 0.09985

<.0001 <.0001 <.0001 <.0001 0.0375 0.2779

258 255 298 296 190 120 120

Pfecal 0.62847 0.57253 0.88230 1.00000 0.41110 0.15131 0.13736

<.0001 <.0001 <.0001 <.0001 0.0990 0.1346

256 253 296 296 190 120 120

Purinary 0.25405 0.25729 0.43404 0.41110 1.00000 -0.07017 -0.13714

0.0014 0.0011 <.0001 <.0001 0.4705 0.1570

156 157 190 190 190 108 108

Caintake 0.28243 0.16195 0.19019 0.15131 -0.07017 1.00000 0.87440

0.0028 0.0956 0.0375 0.0990 0.4705 <.0001

110 107 120 120 108 120 120

Cafecal 0.21877 0.09847 0.09985 0.13736 -0.13714 0.87440 1.00000

0.0217 0.3129 0.2779 0.1346 0.1570 <.0001

110 107 120 120 108 120 120

Caurinary -0.15935 -0.19332 -0.08917 -0.03024 0.21190 -0.15022 -0.37664

0.1171 0.0605 0.3587 0.7560 0.0277 0.1207 <.0001

98 95 108 108 108 108 108

Est_End_P 0.71528 0.71757 0.49039 0.41516 0.16752 -0.09663 -0.13630

<.0001 <.0001 <.0001 <.0001 0.0348 0.3221 0.1616

206 209 235 233 159 107 107

Est_End_Ca 0.70384 0.72946 0.49394 0.41148 0.18882 -0.09660 -0.13629

<.0001 <.0001 <.0001 <.0001 0.0198 0.3223 0.1616

202 204 230 228 152 107 107

MetabolazibleP -0.09436 0.02244 -0.39524 -0.75336 -0.22884 -0.00369 -0.07909

0.2837 0.7977 <.0001 <.0001 0.0086 0.9736 0.4772

131 133 157 157 131 83 83

MetabolazibleCa -0.01296 0.08125 0.04661 -0.14377 -0.06907 -0.43698 -0.63219

0.9121 0.4853 0.6795 0.2004 0.5401 <.0001 <.0001

75 76 81 81 81 81 81

Pearson Correlation Coefficients

Prob > |r| under H0: Rho=0

Number of Observations

Est_End_ Metabolazible Metabolazible

Caurinary Est_End_P Ca P Ca

Ca_P_Ratio -0.09075 -0.46071 -0.45790 0.20859 -0.39797

0.3692 <.0001 <.0001 0.0156 0.0003

100 200 196 134 77

Means overall 13:45 Saturday, March 13, 2024 62

The CORR Procedure

Pearson Correlation Coefficients

Prob > |r| under H0: Rho=0

Number of Observations

Est_End_ Metabolazible Metabolazible

Caurinary Est_End_P Ca P Ca

Foragediet -0.02443 -0.70779 -0.69320 0.15322 -0.20123

0.8212 <.0001 <.0001 0.0830 0.0948

88 202 198 129 70

CPdiet -0.25256 -0.07592 -0.06492 0.33736 -0.43971

0.1496 0.3447 0.4253 0.0017 0.0133

34 157 153 84 31

NDFdiet 0.37142 -0.02470 -0.02103 -0.06019 0.02474

0.0306 0.7634 0.8004 0.5888 0.8949

34 151 147 83 31

EEdiet . 0.35420 0.49644 0.01604 .

. 0.0135 0.0004 0.9271 .

2 48 46 35 2

StarchDiet . -0.14466 -0.20624 -0.51258 .

. 0.4627 0.3121 0.0074 .

0 28 26 26 0

BW -0.04023 0.99968 0.99970 0.02826 0.24054

0.6926 <.0001 <.0001 0.7253 0.0305

99 235 230 157 81

DMI -0.15935 0.71528 0.70384 -0.09436 -0.01296

0.1171 <.0001 <.0001 0.2837 0.9121

98 206 202 131 75

MY -0.19332 0.71757 0.72946 0.02244 0.08125

0.0605 <.0001 <.0001 0.7977 0.4853

95 209 204 133 76

Pintake -0.08917 0.49039 0.49394 -0.39524 0.04661

0.3587 <.0001 <.0001 <.0001 0.6795

108 235 230 157 81

Pfecal -0.03024 0.41516 0.41148 -0.75336 -0.14377

0.7560 <.0001 <.0001 <.0001 0.2004

108 233 228 157 81

Purinary 0.21190 0.16752 0.18882 -0.22884 -0.06907

0.0277 0.0348 0.0198 0.0086 0.5401

108 159 152 131 81

Caintake -0.15022 -0.09663 -0.09660 -0.00369 -0.43698

0.1207 0.3221 0.3223 0.9736 <.0001

108 107 107 83 81

Cafecal -0.37664 -0.13630 -0.13629 -0.07909 -0.63219

<.0001 0.1616 0.1616 0.4772 <.0001

108 107 107 83 81

Caurinary 1.00000 -0.03481 -0.03480 -0.24461 -0.03795

0.7323 0.7324 0.0258 0.7366

108 99 99 83 81

Est_End_P -0.03481 1.00000 1.00000 0.03026 0.24475

0.7323 <.0001 0.7068 0.0277

99 235 228 157 81

Est_End_Ca -0.03480 1.00000 1.00000 0.04413 0.24464

0.7324 <.0001 0.5905 0.0277

99 228 230 151 81

MetabolazibleP -0.24461 0.03026 0.04413 1.00000 0.28393

0.0258 0.7068 0.5905 0.0199

83 157 151 157 67

Means overall 13:45 Saturday, March 13, 2024 63

The CORR Procedure

Pearson Correlation Coefficients

Prob > |r| under H0: Rho=0

Number of Observations

Est_End_ Metabolazible Metabolazible

Caurinary Est_End_P Ca P Ca

MetabolazibleCa -0.03795 0.24475 0.24464 0.28393 1.00000

0.7366 0.0277 0.0277 0.0199

81 81 81 67 81

Means overall 13:45 Saturday, March 13, 2024 64

The Mixed Procedure

Model Information

Data Set WORK.METABOLIZABLE

Dependent Variable MetabolazibleP

Weight Variable InverseTruncSEMPfecal

Polled

Covariance Structure Unstructured

Subject Effect Study

Estimation Method REML

Residual Variance Method Profile

Fixed Effects SE Method Model-Based

Degrees of Freedom Method Containment

Class Level Information

Class Levels Values

Study 61 2 3 5 7 8 9 11 12 14 15 16 17

18 19 20 21 22 23 24 25 26 27

28 29 31 32 33 35 36 37 38 39

40 46 47 48 50 51 52 53 54 55

56 57 58 61 62 63 64 65 66 67

68 69 70 71 72 73 74 75 76

Dimensions

Covariance Parameters 2

Columns in X 2

Columns in Z Per Subject 1

Subjects 61

Max Obs Per Subject 18

Observations Used 129

Observations Not Used 169

Total Observations 298

Iteration History

Iteration Evaluations -2 Res Log Like Criterion

0 1 -92.90054218

1 2 -217.05305321 0.00004441

2 1 -217.06336187 0.00000010

3 1 -217.06338469 0.00000000

Convergence criteria met.

Covariance Parameter Estimates

Standard Z

Cov Parm Subject Estimate Error Value Pr Z

UN(1,1) Study 0.01886 0.004931 3.82 <.0001

Residual 0.003749 0.000551 6.80 <.0001

Fit Statistics

-2 Res Log Likelihood -217.1

AIC (smaller is better) -213.1

AICC (smaller is better) -213.0

BIC (smaller is better) -208.8

Null Model Likelihood Ratio Test

DF Chi-Square Pr > ChiSq

1 124.16 <.0001

Means overall 13:45 Saturday, March 13, 2024 65

The Mixed Procedure

Solution for Fixed Effects

Standard

Effect Estimate Error DF t Value Pr > |t|

Intercept 0.6365 0.08030 35 7.93 <.0001

Foragediet 0.000077 0.000119 92 0.65 0.5157

Type 3 Tests of Fixed Effects

Num Den

Effect DF DF F Value Pr > F

Foragediet 1 92 0.43 0.5157

Means overall 13:45 Saturday, March 13, 2024 66

The Mixed Procedure

Model Information

Data Set WORK.METABOLIZABLE

Dependent Variable MetabolazibleP

Weight Variable InverseTruncSEMPfecal

Polled

Covariance Structure Unstructured

Subject Effect Study

Estimation Method REML

Residual Variance Method Profile

Fixed Effects SE Method Model-Based

Degrees of Freedom Method Containment

Class Level Information

Class Levels Values

Study 42 2 3 4 7 8 9 11 12 14 29 30 31

32 33 34 35 37 38 39 40 46 47

48 50 51 52 53 56 57 61 64 65

66 67 68 69 70 71 72 73 74 75

Dimensions

Covariance Parameters 2

Columns in X 2

Columns in Z Per Subject 1

Subjects 42

Max Obs Per Subject 18

Observations Used 83

Observations Not Used 215

Total Observations 298

Iteration History

Iteration Evaluations -2 Res Log Like Criterion

0 1 -53.99332949

1 2 -136.57917565 0.00308182

2 1 -137.09646003 0.00034746

3 1 -137.15005606 0.00000594

4 1 -137.15091591 0.00000000

Convergence criteria met.

Covariance Parameter Estimates

Standard Z

Cov Parm Subject Estimate Error Value Pr Z

UN(1,1) Study 0.02194 0.007388 2.97 0.0015

Residual 0.004131 0.000755 5.47 <.0001

Fit Statistics

-2 Res Log Likelihood -137.2

AIC (smaller is better) -133.2

AICC (smaller is better) -133.0

BIC (smaller is better) -129.7

Null Model Likelihood Ratio Test

DF Chi-Square Pr > ChiSq

1 83.16 <.0001

Means overall 13:45 Saturday, March 13, 2024 67

The Mixed Procedure

Solution for Fixed Effects

Standard

Effect Estimate Error DF t Value Pr > |t|

Intercept 0.6328 0.1071 21 5.91 <.0001

NDFdiet 0.000129 0.000294 60 0.44 0.6615

Type 3 Tests of Fixed Effects

Num Den

Effect DF DF F Value Pr > F

NDFdiet 1 60 0.19 0.6615

Means overall 13:45 Saturday, March 13, 2024 68

The Mixed Procedure

Model Information

Data Set WORK.METABOLIZABLE

Dependent Variable MetabolazibleP

Weight Variable InverseTruncSEMPfecal

Polled

Covariance Structure Unstructured

Subject Effect Study

Estimation Method REML

Residual Variance Method Profile

Fixed Effects SE Method Model-Based

Degrees of Freedom Method Containment

Class Level Information

Class Levels Values

Study 14 4 7 9 31 32 48 51 52 58 64 71

72 74 75

Dimensions

Covariance Parameters 2

Columns in X 2

Columns in Z Per Subject 1

Subjects 14

Max Obs Per Subject 12

Observations Used 35

Observations Not Used 263

Total Observations 298

Iteration History

Iteration Evaluations -2 Res Log Like Criterion

0 1 -20.32032600

1 3 -59.55276688 0.01215929

2 2 -59.68807638 0.00342031

3 1 -59.93127294 0.00041776

4 1 -59.95860041 0.00000845

5 1 -59.95911692 0.00000000

Convergence criteria met.

Covariance Parameter Estimates

Standard Z

Cov Parm Subject Estimate Error Value Pr Z

UN(1,1) Study 0.01998 0.01020 1.96 0.0251

Residual 0.003677 0.001055 3.48 0.0002

Fit Statistics

-2 Res Log Likelihood -60.0

AIC (smaller is better) -56.0

AICC (smaller is better) -55.6

BIC (smaller is better) -54.7

Null Model Likelihood Ratio Test

DF Chi-Square Pr > ChiSq

1 39.64 <.0001

Means overall 13:45 Saturday, March 13, 2024 69

The Mixed Procedure

Solution for Fixed Effects

Standard

Effect Estimate Error DF t Value Pr > |t|

Intercept 0.8212 0.1026 9 8.00 <.0001

EEdiet -0.00408 0.002784 24 -1.47 0.1555

Type 3 Tests of Fixed Effects

Num Den

Effect DF DF F Value Pr > F

EEdiet 1 24 2.15 0.1555

Means overall 13:45 Saturday, March 13, 2024 70

The Mixed Procedure

Model Information

Data Set WORK.METABOLIZABLE

Dependent Variable MetabolazibleP

Weight Variable InverseTruncSEMPfecal

Polled

Covariance Structure Unstructured

Subject Effect Study

Estimation Method REML

Residual Variance Method Profile

Fixed Effects SE Method Model-Based

Degrees of Freedom Method Containment

Class Level Information

Class Levels Values

Study 44 2 3 4 7 8 9 11 12 14 29 30 31

32 33 34 35 36 37 38 39 40 46

47 48 50 51 52 53 56 57 58 61

64 65 66 67 68 69 70 71 72 73

74 75

Dimensions

Covariance Parameters 2

Columns in X 2

Columns in Z Per Subject 1

Subjects 44

Max Obs Per Subject 18

Observations Used 84

Observations Not Used 214

Total Observations 298

Iteration History

Iteration Evaluations -2 Res Log Like Criterion

0 1 -69.73451284

1 2 -131.89565579 0.02630002

2 1 -136.70439360 0.01162342

3 1 -138.79621148 0.00337340

4 1 -139.37409221 0.00042093

5 1 -139.44040546 0.00000877

6 1 -139.44169693 0.00000000

Convergence criteria met.

Covariance Parameter Estimates

Standard Z

Cov Parm Subject Estimate Error Value Pr Z

UN(1,1) Study 0.02467 0.008490 2.91 0.0018

Residual 0.003996 0.000744 5.37 <.0001

Fit Statistics

-2 Res Log Likelihood -139.4

AIC (smaller is better) -135.4

AICC (smaller is better) -135.3

BIC (smaller is better) -131.9

Means overall 13:45 Saturday, March 13, 2024 71

The Mixed Procedure

Null Model Likelihood Ratio Test

DF Chi-Square Pr > ChiSq

1 69.71 <.0001

Solution for Fixed Effects

Standard

Effect Estimate Error DF t Value Pr > |t|

Intercept 0.7902 0.1240 22 6.37 <.0001

CPdiet -0.00062 0.000711 60 -0.87 0.3882

Type 3 Tests of Fixed Effects

Num Den

Effect DF DF F Value Pr > F

CPdiet 1 60 0.76 0.3882

Means overall 13:45 Saturday, March 13, 2024 72

The Mixed Procedure

Model Information

Data Set WORK.METABOLIZABLE

Dependent Variable MetabolazibleP

Weight Variable InverseTruncSEMPfecal

Polled

Covariance Structure Unstructured

Subject Effect Study

Estimation Method REML

Residual Variance Method Profile

Fixed Effects SE Method Model-Based

Degrees of Freedom Method Containment

Class Level Information

Class Levels Values

Study 7 7 9 14 32 33 74 75

Dimensions

Covariance Parameters 2

Columns in X 2

Columns in Z Per Subject 1

Subjects 7

Max Obs Per Subject 12

Observations Used 26

Observations Not Used 272

Total Observations 298

Iteration History

Iteration Evaluations -2 Res Log Like Criterion

0 1 -31.16974220

1 2 -45.39580644 0.00241330

2 1 -45.52439913 0.00033378

3 1 -45.54078883 0.00000880

4 1 -45.54119070 0.00000001

Convergence criteria met.

Covariance Parameter Estimates

Standard Z

Cov Parm Subject Estimate Error Value Pr Z

UN(1,1) Study 0.008419 0.006860 1.23 0.1098

Residual 0.002991 0.000948 3.16 0.0008

Fit Statistics

-2 Res Log Likelihood -45.5

AIC (smaller is better) -41.5

AICC (smaller is better) -41.0

BIC (smaller is better) -41.6

Null Model Likelihood Ratio Test

DF Chi-Square Pr > ChiSq

1 14.37 0.0002

Means overall 13:45 Saturday, March 13, 2024 73

The Mixed Procedure

Solution for Fixed Effects

Standard

Effect Estimate Error DF t Value Pr > |t|

Intercept 0.6150 0.04995 4 12.31 0.0003

StarchDiet -0.00002 0.000096 20 -0.21 0.8368

Type 3 Tests of Fixed Effects

Num Den

Effect DF DF F Value Pr > F

StarchDiet 1 20 0.04 0.8368

Means overall 13:45 Saturday, March 13, 2024 74

The Mixed Procedure

Model Information

Data Set WORK.METABOLIZABLE

Dependent Variable MetabolazibleP

Weight Variable InverseTruncSEMPfecal

Polled

Covariance Structure Unstructured

Subject Effect Study

Estimation Method REML

Residual Variance Method Profile

Fixed Effects SE Method Model-Based

Degrees of Freedom Method Containment

Class Level Information

Class Levels Values

Study 53 2 3 4 7 8 9 12 14 15 16 17 18

19 20 21 22 23 24 25 26 27 28

29 30 31 32 33 34 35 36 37 38

40 46 47 48 52 53 55 56 57 58

59 60 61 66 67 68 71 73 74 75

76

Dimensions

Covariance Parameters 2

Columns in X 2

Columns in Z Per Subject 1

Subjects 53

Max Obs Per Subject 18

Observations Used 134

Observations Not Used 164

Total Observations 298

Iteration History

Iteration Evaluations -2 Res Log Like Criterion

0 1 -91.25855742

1 3 -232.14085156 0.00199092

2 2 -245.33438232 0.25805797

3 2 -250.88033316 0.00009452

4 1 -250.90235874 0.00000058

5 1 -250.90250141 0.00000000

Convergence criteria met.

Covariance Parameter Estimates

Standard Z

Cov Parm Subject Estimate Error Value Pr Z

UN(1,1) Study 0.01917 0.004960 3.86 <.0001

Residual 0.003376 0.000479 7.05 <.0001

Fit Statistics

-2 Res Log Likelihood -250.9

AIC (smaller is better) -246.9

AICC (smaller is better) -246.8

BIC (smaller is better) -243.0

Means overall 13:45 Saturday, March 13, 2024 75

The Mixed Procedure

Null Model Likelihood Ratio Test

DF Chi-Square Pr > ChiSq

1 159.64 <.0001

Solution for Fixed Effects

Standard

Effect Estimate Error DF t Value Pr > |t|

Intercept 0.5886 0.04226 34 13.93 <.0001

Ca_P_Ratio 0.03937 0.01259 98 3.13 0.0023

Type 3 Tests of Fixed Effects

Num Den

Effect DF DF F Value Pr > F

Ca_P_Ratio 1 98 9.78 0.0023

Means overall 13:45 Saturday, March 13, 2024 76

The Mixed Procedure

Model Information

Data Set WORK.METABOLIZABLE

Dependent Variable MetabolazibleCa

Weight Variable InverseTruncSEMCafecal

Polled

Covariance Structure Unstructured

Subject Effect Study

Estimation Method REML

Residual Variance Method Profile

Fixed Effects SE Method Model-Based

Degrees of Freedom Method Containment

Class Level Information

Class Levels Values

Study 42 2 3 4 7 8 9 11 12 14 29 30 31

32 33 34 35 37 38 39 40 46 47

48 50 51 52 53 56 57 61 64 65

66 67 68 69 70 71 72 73 74 75

Dimensions

Covariance Parameters 2

Columns in X 2

Columns in Z Per Subject 1

Subjects 42

Max Obs Per Subject 18

Observations Used 31

Observations Not Used 267

Total Observations 298

Iteration History

Iteration Evaluations -2 Res Log Like Criterion

0 1 -24.77079044

1 3 -56.99766653 0.00037315

2 2 -59.16431794 0.04948877

3 2 -59.84168018 0.00031094

4 1 -59.86078288 0.00000646

5 1 -59.86115385 0.00000000

Convergence criteria met.

Covariance Parameter Estimates

Standard Z

Cov Parm Subject Estimate Error Value Pr Z

UN(1,1) Study 0.01507 0.009389 1.61 0.0542

Residual 0.002851 0.000821 3.47 0.0003

Fit Statistics

-2 Res Log Likelihood -59.9

AIC (smaller is better) -55.9

AICC (smaller is better) -55.4

BIC (smaller is better) -52.4

Null Model Likelihood Ratio Test

DF Chi-Square Pr > ChiSq

1 35.09 <.0001

Means overall 13:45 Saturday, March 13, 2024 77

The Mixed Procedure

Solution for Fixed Effects

Standard

Effect Estimate Error DF t Value Pr > |t|

Intercept 0.7504 0.3478 6 2.16 0.0743

NDFdiet -0.00028 0.001067 23 -0.26 0.7969

Type 3 Tests of Fixed Effects

Num Den

Effect DF DF F Value Pr > F

NDFdiet 1 23 0.07 0.7969

Means overall 13:45 Saturday, March 13, 2024 78

The Mixed Procedure

Model Information

Data Set WORK.METABOLIZABLE

Dependent Variable MetabolazibleCa

Weight Variable InverseTruncSEMCafecal

Polled

Covariance Structure Unstructured

Subject Effect Study

Estimation Method REML

Residual Variance Method Profile

Fixed Effects SE Method Model-Based

Degrees of Freedom Method Containment

Class Level Information

Class Levels Values

Study 7 7 9 14 32 33 74 75

Dimensions

Covariance Parameters 2

Columns in X 2

Columns in Z Per Subject 1

Subjects 7

Max Obs Per Subject 12

Observations Used 0

Observations Not Used 298

Total Observations 298

Means overall 13:45 Saturday, March 13, 2024 79

The Mixed Procedure

Model Information

Data Set WORK.METABOLIZABLE

Dependent Variable MetabolazibleCa

Weight Variable InverseTruncSEMCafecal

Polled

Covariance Structure Unstructured

Subject Effect Study

Estimation Method REML

Residual Variance Method Profile

Fixed Effects SE Method Model-Based

Degrees of Freedom Method Containment

Class Level Information

Class Levels Values

Study 14 4 7 9 31 32 48 51 52 58 64 71

72 74 75

Dimensions

Covariance Parameters 2

Columns in X 2

Columns in Z Per Subject 1

Subjects 14

Max Obs Per Subject 12

Observations Used 2

Observations Not Used 296

Total Observations 298

Iteration History

Iteration Evaluations -2 Res Log Like Criterion

0 1 -4.70664506

1 1 -4.70664506 0.00000000

Convergence criteria met but final hessian is not positive

definite.

Covariance Parameter Estimates

Standard Z

Cov Parm Subject Estimate Error Value Pr Z

UN(1,1) Study 2.79E-11 0 . .

Residual 0.000531 0.000751 0.71 0.2398

Fit Statistics

-2 Res Log Likelihood -4.7

AIC (smaller is better) -0.7

AICC (smaller is better) 11.3

BIC (smaller is better) 0.6

Null Model Likelihood Ratio Test

DF Chi-Square Pr > ChiSq

1 0.00 1.0000

Means overall 13:45 Saturday, March 13, 2024 80

The Mixed Procedure

Solution for Fixed Effects

Standard

Effect Estimate Error DF t Value Pr > |t|

Intercept 0.8162 0.01148 0 71.10 .

EEdiet 0 . . . .

Type 3 Tests of Fixed Effects

Num Den

Effect DF DF F Value Pr > F

EEdiet 0 . . .

Means overall 13:45 Saturday, March 13, 2024 81

The Mixed Procedure

Model Information

Data Set WORK.METABOLIZABLE

Dependent Variable MetabolazibleCa

Weight Variable InverseTruncSEMCafecal

Polled

Covariance Structure Unstructured

Subject Effect Study

Estimation Method REML

Residual Variance Method Profile

Fixed Effects SE Method Model-Based

Degrees of Freedom Method Containment

Class Level Information

Class Levels Values

Study 44 2 3 4 7 8 9 11 12 14 29 30 31

32 33 34 35 36 37 38 39 40 46

47 48 50 51 52 53 56 57 58 61

64 65 66 67 68 69 70 71 72 73

74 75

Dimensions

Covariance Parameters 2

Columns in X 2

Columns in Z Per Subject 1

Subjects 44

Max Obs Per Subject 18

Observations Used 31

Observations Not Used 267

Total Observations 298

Iteration History

Iteration Evaluations -2 Res Log Like Criterion

0 1 -40.38994893

1 2 -62.34744284 0.01011797

2 1 -63.08424392 0.00320067

3 1 -63.30762593 0.00049730

4 1 -63.33971755 0.00001689

5 1 -63.34072598 0.00000002

6 1 -63.34072733 0.00000000

Convergence criteria met.

Covariance Parameter Estimates

Standard Z

Cov Parm Subject Estimate Error Value Pr Z

UN(1,1) Study 0.01196 0.007943 1.51 0.0661

Residual 0.002861 0.000829 3.45 0.0003

Fit Statistics

-2 Res Log Likelihood -63.3

AIC (smaller is better) -59.3

AICC (smaller is better) -58.9

BIC (smaller is better) -55.8

Means overall 13:45 Saturday, March 13, 2024 82

The Mixed Procedure

Null Model Likelihood Ratio Test

DF Chi-Square Pr > ChiSq

1 22.95 <.0001

Solution for Fixed Effects

Standard

Effect Estimate Error DF t Value Pr > |t|

Intercept 1.2861 0.5515 6 2.33 0.0585

CPdiet -0.00383 0.003368 23 -1.14 0.2670

Type 3 Tests of Fixed Effects

Num Den

Effect DF DF F Value Pr > F

CPdiet 1 23 1.29 0.2670

Means overall 13:45 Saturday, March 13, 2024 83

The Mixed Procedure

Model Information

Data Set WORK.METABOLIZABLE

Dependent Variable MetabolazibleCa

Weight Variable InverseTruncSEMCafecal

Polled

Covariance Structure Unstructured

Subject Effect Study

Estimation Method REML

Residual Variance Method Profile

Fixed Effects SE Method Model-Based

Degrees of Freedom Method Containment

Class Level Information

Class Levels Values

Study 61 2 3 5 7 8 9 11 12 14 15 16 17

18 19 20 21 22 23 24 25 26 27

28 29 31 32 33 35 36 37 38 39

40 46 47 48 50 51 52 53 54 55

56 57 58 61 62 63 64 65 66 67

68 69 70 71 72 73 74 75 76

Dimensions

Covariance Parameters 2

Columns in X 2

Columns in Z Per Subject 1

Subjects 61

Max Obs Per Subject 18

Observations Used 70

Observations Not Used 228

Total Observations 298

Iteration History

Iteration Evaluations -2 Res Log Like Criterion

0 1 -67.33541494

1 2 -118.40514869 0.00000000

Convergence criteria met.

Covariance Parameter Estimates

Standard Z

Cov Parm Subject Estimate Error Value Pr Z

UN(1,1) Study 0.01275 0.005170 2.47 0.0068

Residual 0.003844 0.000767 5.01 <.0001

Fit Statistics

-2 Res Log Likelihood -118.4

AIC (smaller is better) -114.4

AICC (smaller is better) -114.2

BIC (smaller is better) -110.2

Null Model Likelihood Ratio Test

DF Chi-Square Pr > ChiSq

1 51.07 <.0001

Means overall 13:45 Saturday, March 13, 2024 84

The Mixed Procedure

Solution for Fixed Effects

Standard

Effect Estimate Error DF t Value Pr > |t|

Intercept 0.6868 0.1141 18 6.02 <.0001

Foragediet -0.00011 0.000156 50 -0.72 0.4751

Type 3 Tests of Fixed Effects

Num Den

Effect DF DF F Value Pr > F

Foragediet 1 50 0.52 0.4751
