# Supplementary material for: A new system of phosphorus and calcium requirements for lactating dairy cows
Source: PLoS One. 2024 Aug 29;19(8):e0308889. doi: 10.1371/journal.pone.0308889 (PMC11361663; doi:10.1371/journal.pone.0308889)
Supplement: S3 File — (DOCX) [file pone.0308889.s003.docx]

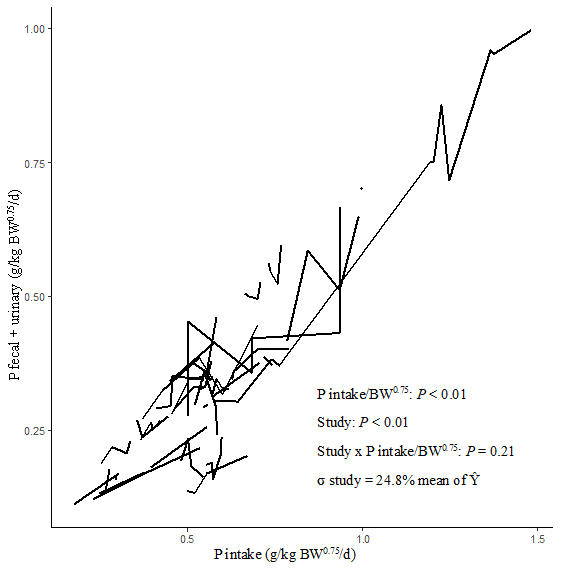


**Supporting information S3.1. Relationship between the sum of daily phosphorus (P) total fecal and urinary excretion and P intake**. The solids lines are the observed values of each study (39 balance trials). The study was evaluated as a random effect. σ study = square root of the estimated study variance. BW = body weight.


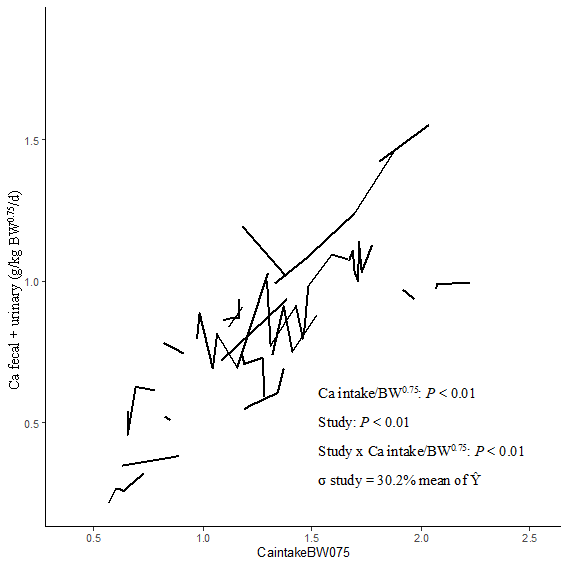


**Supporting information S3.2.** **Relationship between the sum of daily phosphorus (Ca) total fecal and urinary excretion and Ca intake**. The solids lines are the observed values of each study (23 balance trials). The study was evaluated as a random effect. σ study = square root of the estimated study variance. BW = body weight.
