# Supplementary material for: A new system of phosphorus and calcium requirements for lactating dairy cows
Source: PLoS One. 2024 Aug 29;19(8):e0308889. doi: 10.1371/journal.pone.0308889 (PMC11361663; doi:10.1371/journal.pone.0308889)
Supplement: Attachment — Submitted filename: Response to Reviewers.doc [file pone.0308889.s005.doc]

Dear Dr Aziz ur Rahman Muhammad,

Academic Editors

PLOS ONE

Thank you for your comments, criticisms, and suggestions regarding our manuscript PONE-D-2409154, “A new system of phosphorus and calcium requirements for lactating dairy cows”.

Please find below our responses to each point raised by you and the reviewers.

**Journal Requirements Addressed:**

1. The file naming and manuscript style were addressed to meet PLOS ONE`s style requirements.
2. The Grant information provided in “Funding information” and “Financial Disclosure” had been corrected.
3. The financial disclosure statement has been amended as requirement.
4. Please note that now the results on interactions between P and Ca excretions with milk yield group have been provided in Results and Table 2.
5. Reference list was revised.

**Additional Editor Comments:** Reviewers has reviewed the manuscript and all of the 3 reviewers suggested minor revision. Therefore, I would like to invite you for minor revision that are mainly related to English language and grammar mistakes. please carefully address other issues related to your manuscript in the revised version of the manuscript.

Author response: The manuscript has been reviewed. English language and grammar mistakes have been corrected. Thank you.

Sincerely,

André Oliveira

Corresponding Author

**Response to Reviewer #1:**

Thank you for your comments, criticisms and suggestions.

Please find below responses for your questions.

**Reviewer #1 Comments:**

**L34-35: perhaps reword to “We estimated the net requirements of P and Ca for maintenance from the intercept of a nonlinear equation between mineral intake and the sum of fecal and urinary excretions, which is an estimate of endogenous P and Ca losses.”
L40: “… ) using a 5-fold cross validation approach.”
L43: “… means, respectively.”
L46: “were” or “are”?
L46: “… for lactating dairy…”
L49: “… sustainability of the dairy…”
L62: define AC here at first use.
L64-66: “…, we propose here the term metabolizable coefficient as opposed to AC.”
L71: “… adopted to estimate endogenous…”
L91-92: abbreviate to MC
L98: abbreviate to MC
L107: delete one of the “P and Ca”
L112: “review” instead of “revision” in all instances that it is used similarly.
L126: “reported in studies”
L126: “were” instead of “are”
L150: missing a period
L188-189: I recommend entering this equation using the “Equation” functionality of Microsoft word (under “Insert” and then “Equation”)
L198: should this be “studentized residual”?
L206: should this be “… using the 5-fold cross-validation approach…”?
L224: “genotype” or “breed”?
L351: “… introduced the term…”
L354: “… the concept of metabolizable mineral aligns…”
L364-365: “… is feasibly measured in a commercial…”
L424 “Keanthao et al. [50] reported…”
L427: “… to adequately estimate P…”
L434: “highlighted”
L436: “NASEM”
L436-437: reword.
L439: delete “be”**

Author Response: Thank you for all your suggestions. All suggestions have been addressed.

**L109: this meta-analysis focused on P and Ca, but you only used search terms of “dairy cows” and “phosphorus”? Why wasn’t “calcium” used as a search term?**
Author Response: As our initial objective was included on P balance trial, the used search terms were “dairy cows” and “phosphorus”. However, after selection of the studies we observed that several studies also provided information on Ca balance. Therefore, we decided derived P and Ca requirements models for lactating dairy cows.

**L110: was the second systematic review done using the same search terms?**

Author Response: Yes, we used the same search terms in the second systematic review as described in manuscript.

**L186: hasn’t MC already been defined?**

Author Response: Yes, but abbreviations should not be used at the beginning of a sentence

**L226-227: was urine P and Ca exclusively measured by total collection? Did not all studies measure urine output by total collection? Why would they measure urine output by total collection and then use markers to assess fecal output in the same study?**

Author Response: Please note that information on fecal and urinary outputs methods has been provided since the first version of our manuscript, Fecal and urinary P and Ca excretions were obtained by total collection in 75.6% of observations; other studies (24.4% observations) used ytterbium (6.5% observations), Cr_2_O_3_ (5.5%), indigestible NDF or acid detergent fiber 288 h (3.6%), n-alkanes (3.6%), and lignin, TiO_2_ and other (5.2%) as fecal output markers, and creatinine urine as urinary output marker.

**L244-245: Did you describe in the methods how mean and slope biases were assessed? Also, I don’t know if slope being not different from 1 necessarily indicates linear bias. St-Pierre (2003; DOI: 10.3168/jds.S0022-0302(03)73612-1) suggests regressing residuals (obs – predicted) against mean centered predicted values, then the intercept of this regression is mean bias, and the slope is slope bias.**
Author Response: The method to assess mean and slope biases was included in Material and Methods. Thank you.

**L307-308: was this a significant difference between Jersey and Holsteins? The standard errors make it seem as if they would not be statistically different.**

Author Response: Thank you for your comments. In this new version, we have used confidence interval 95% to evaluated the difference between mineral milk concentration between Jersey and Holstein. The mean P milk concentration was 0.90 (95% CI; 0.89, 0.92) g/kg for Holstein and 1.00 (95% CI; 0.96, 1.04) g/kg for Jersey, while Ca milk concentration was 1.18 (95% CI; 1.12, 1.23) g/kg for Holstein and 1.38 (95% CI; 1.28, 1.47) g/kg for Jersey (Figure 5). Therefore, P and Ca milk concentration of Holstein were lower (P ≤ 0.05) than Jersey.

**L356-358: If MC and AC are so similar, what is the benefit of using MC over AC?**

Author Response: The benefit is conceptual. The term “absorption coefficient” is correct if MC was obtained only from fecal excretion. We proposed that when urinary excretion is included, the term “metabolizable coefficient” seems more appropriate.

Sincerely,

André Oliveira

Corresponding Author

**Response to Reviewer #2:**

Thank you for your comments, criticisms and suggestions.

Please find below responses for your questions.

**General comments: This meta-analysis developed and evaluated models to estimate Ca and P requirements for lactating dairy cattle using NL mixed models. Overall, the methodology is adequate, and the manuscript is well-written. The limitations of this study were well-discussed and justified. The parameter estimates had low standard errors, and cross-evaluation indicated opportunities for practical applications. Additionally, the authors recognized that an additional model evaluation using independent studies is recommended. For this reviewer, the major issue is to clarify if growing animals or late gestation animals were used in this study. If yes, the authors need to justify why they did not explore those requirements. Therefore, please find below some comments for consideration.**

Author response: We appreciate your positives comments. It was not our aim to derive models of P and Ca requirement for growing or late gestation dairy cattle. Our objective was clear in to derivate a new system of P and Ca net requirements for maintenance and lactation, and dietary metabolizable coefficient of P and Ca for lactating dairy cows. Therefore, we built a large and comprehensive dataset of balance trials only with lactating dairy cows. We estimated the net requirements for maintenance from the intercept of a nonlinear equation between mineral intake and the sum of total fecal and urinary excretions, which is an estimate of endogenous mineral loss. Mineral secreted in milk was used to obtain net requirements for lactation. The mineral metabolizable coefficient was quantified through observed (treatment means) mineral intake and total fecal and urinary excretions, discounting the estimated endogenous excretions from our proposed models. Mineral requirements for growing represents the body mineral accretion, while mineral requirements for pregnancy represents the fetal mineral accretion. No information on body or fetal mineral accretions were provided in selected studies in our dataset. Thank you.

**Title: Use "A new system of phosphorus and calcium requirements for lactating dairy cows"**
Author Response: Your suggestion of title was accepted. Thank you.

**L33: Use “articles or studies” instead of “papers” across the entire manuscript.
L37: Use “treatment means”.
L40: “.. using a 5-fold cross-validation approach…” instead of “...from the..”.
L46: “lactating” instead of “lactation”.
L107: Rewrite this sentence, something like: “A systematic review of mineral balance trials published as peer-review publications (xx studies) was performed. Treatment means were used to develop models to estimate P and Ca requirement for lactating dairy cows”.
L124: Remove this sentence “...(53 from previous review and four from new 124 revision).."
L140 and L177: Delete redundant information.
L66/98/186/L194: Revise abbreviation use for metabolizable coefficient (MC) across the entire manuscript.
L221: Add the DMI range.
L236: Remove the space before the comma.
L244: Use "slope bias" instead of “slop”.
L334: Include a discussion about internal and external model evaluation. Also, discuss the % of the data (treatment means) that was from early, middle and late lactation. Also, the authors need to discuss the methods used to estimate Ca and P excretion in feces and their limitations (markers)..
L395: Include a period in this phrase “… ecosystems [3, 4]. Our…”.
L416-424: Fix parenthesis.
L436: Fix a typo “NSAEM (2021)”**

Author Response: Thank you for your suggestions and corrections. All corrections and suggestions were accepted.

**L33: Please describe the database used in the abstract (breed, averages for DMI and milk yield x±xx).**
Author Response: Unfortunately the inclusion this information will exceed the limits of word in the abstract

**L93: Remove the word "system" and use "model."**
Author Response: I respect your idiosyncrasy, but I see no reason to replace the word “system for “model” here.

**Table 1: Confirm if that is the final database used to develop the models, if not, please describe only the final database used for model fitting. Also, describe the frequency for parity (primiparous, multiparous, or both) and fecal collection methods (total or estimated using markers) using the final database.**

Author Response: The Table 1 contains the descriptive statistics of the our complete dataset which is available in an Excel file [21]. The frequency for fecal collection methods is described in results since first version of manuscript. Parity frequency was added. Multiparous cows dataset were reported in 31 studies, primiparous in three studies, multiparous and primiparous cows in eight studies, and no parity cows was reported in 34 studies. Please note that each model or outcome (P fecal+urinary excretion; Ca fecal+urinary excretion; P-MC; Ca-MC; milk P; and milk Ca) were fitted with different number of studies and treatment means due to the availability of reported inputs in articles. In addition, please note that the final dataset, codes and outputs each model are described in Supplementary Information S2.

**L126: Clarify this sentence “Data not reported on studies were evaluated as missing data; then, they are subsequently excluded from the final model.” How was the evaluation conducted? Do the authors mean summarized using descriptive statistics?**

Author Response: The sentence was clarified. No procedure to estimate missing data was adopted, except for the SEM of mineral fecal excretion. Data not reported in articles were evaluated as missing data; then, they were not used in final models.

**L139: Describe how many treatment means were dropped as outliers.**

Author Response: The number of treatment means dropped as outliers have been described since first version in Table 1 and in Complete Dataset in an Excel file [21].

**L149: Describe the N or % of treatment means that did not report SEM in your database. Include in the discussion section the methods used as weight in meta-analysis and why did the authors use SEM instead of other methods (i.e. N of animals / trt).**

Author Response: The observations number of SEM for P and Ca fecal excretions were added in Table 1. The inverse of reported SEM is the gold-method for studies weighting in meta-analysis when treatment means are used as observations (St-Pierre, 2001. https://doi.org/10.3168/jds.S0022-0302(01)74530-4; Sauvant et al., 2020. 10.1017/s1751731120001688). The number of animals or experimental units per treatment (n/trt) has limitations as studies-weighting method, because studies with same n/trt can be differences in experimental precision of the treatment means (i.e. SEM). Therefore, n/trt should only be used if SEM values were not reported. I see no reason to include a discussion why I adopted a gold-method (inverse SEM) instead a limited method (i.e. N/trt) for studies-weighting. In addition, I did not compare methods for studies-weighting. The inclusion this discussion is redundant.

**L153: What is the mature BW for each breed used in this database? Based on the minimum BW and maximum DIM, the authors need to discuss if some cows had growth/pregnancy requirements for Ca and P which were not explored in this study. If yes, include it in the limitation of study.**

Author Response: Mature BW were note reported in studies. However, typically mature BW for actual Holstein cows is ~ 680 kg and Jersey cow is ~ 450 kg. This question about growth/pregnancy requirement was previously addressed. Our dataset contains only lactating dairy cows with 316 to 754 kg BW (Table 1; [21].

**L159: Describe how the initial values for parameters of the NL mixed models were identified. Describe it.**

Author Response: The initial values were defined through of interactive approach. Please note that the initial values for estimates of the NL model are provided in S2.

**L162: Check if the intercept β1 is significant in this model, include it in the results section.**

Author Response: The intercepts are significant, and its P-values were included in Table 2. Thank you.

**L159: Explain why a nonlinear exponential mixed model was chosen over other models (i.e., linear mixed models with linear or quadratic terms, did you test it or that was due to data visualization?).**

Author Response: Nonlinear models are widely known for better to describe physical, chemical and biological phenomena than linear models. We initially also tested linear models (i.e. linear or quadradic function), but its models provided negative or null intercepts, indicating a biological inconsistency (negative or null endogenous excretion). In addition, the proposed exponential model presented better fir to the data, and a positive and coherent intercept (estimated endogenous excretion).

**L221: Clarify if some animals were growing in this database (316 to 754 kg).**
Author Response: Our dataset contains only lactating dairy cows with 316 to 754 kg BW.

**L331: Include a discussion about outliers in meta-analysis here. Also, is the low milk yield due the diet? For example, treatment means from the pasture system had lower milk. Discuss it.**

Author Response: This comments is inconsistent because a discussion about outliers has been included since the first version of our manuscript. In addition, the evaluation of factors affecting milk yield was not our objective. Milk yield is affect by genetic, environmental and interaction genetic x environmental. The central point of our discussion here is that our primary objective was to establish a new factorial P and Ca requirements system for maintenance and lactation from a meta-regression of a comprehensive mineral balance trials database. Therefore, our dataset (lactating dairy cows with 316 to 754 kg BW and 4.5 to 52.8 kg/d) is aligned with our objective of deriving a comprehensive requirement system. On the other hand, we seek to remove potential outliers arising from factors such as milk yield from analysis based on studentized residual.

**L448/449: Explain the main limitations of cross-validation in terms of model evaluation.**

Author Response: Here, we do not consider that the use of the K- fold cross-validation approach is a limitation in our study. K- fold cross-validation approach is a standard-method for model evaluation if the folds were independents, as our study. The limitation is that we were able to evaluate P and Ca excretions models used to estimate endogenous excretions, but not for P and Ca dietary requirements. The evaluation of dietary requirements prediction should be done through comparation between predicted with observed values from dose-response experiments. Unfortunately, the limited number of dose-response experiments restricted our ability to evaluated our models and other, such as NASEM (2021) and NRC (2001). Therefore, here we have highlighted that the predictive models of P and Ca dietary requirements still needs to be further evaluated through independent response-dose feeding experiments.

Sincerely,

André Oliveira

Corresponding Author

**Response to Reviewer #3:**

Thank you for your comments, criticisms and suggestions.

Please find below responses for your questions.

**General comments: Overall, this study has merit as it aims to develop a new model for predicting the calcium and phosphorous requirements of dairy cows. Previous requirements (NASEM and NRC) were predicted from equations derived from a rather small sample size which this study aims to overcome by including a far larger sample size in their regression analysis and subsequent prediction equations. It is not clear to the reader whether the studies used to create the NASEM and NRC predictions are also included in the present study (after checking the reference list and excel file, it seems they are (?)) – this should be highlighted as it adds merit and credibility to the present study.
I have answered No to question number 4 as the current manuscript requires English language editing to avoid ambiguities and improve understanding. Additionally, there are a number of typos throughout the manuscript that should be corrected before publishing e.g. L46, “lactation” should read “lactating”, L47, “requirement” should read “requirements”, L49, should read “in the dairy industry”… etc.
The introduction is well thought out and lays a solid foundation for the study. The discussion requires reformulation and rewriting.**
Author Response: Thank you for your positives comments. Our proposed models to estimate endogenous excretion and MC were derived from a different approach in relation to NASEM and NRC models. Since the first version of our manuscript, we have described a clear information about methods and dataset used by NASEM (2021) committee, and in contrast with our model. The NASEM (2021) proposed an overall value of endogenous fecal excretion of 1 g P/kg DMI. This value derived from treatment means analysis of only three P balance trials with lactating dairy cows [13, 14, 15], and it was calculated assuming a true absorption coefficient of 0.80 [12]. The endogenous urinary excretion of 0.0006 g P/kg body weight was proposed from analysis of treatment means in three studies with lactating dairy cows [12]. The proposed net requirement for maintenance of Ca (0.90 g Ca/kg DMI) by NASEM 2021 [12] was derived from a regression between endogenous fecal excretion and DMI from treatment means of five studies where endogenous fecal excretion was obtained using intravenously injected radioisotopes of Ca [7, 16, 17, 9, 18], but only one involving lactating dairy cows [8]. In contrast, we derived new values of endogenous excretion and MC from a meta-regression of a comprehensive mineral balance trials database with only lactating cows. We estimated the net requirements for maintenance from the intercept of a nonlinear equation between mineral intake and the sum of total fecal and urinary excretions, which is an estimate of endogenous mineral loss. Mineral secreted in milk was used to obtain net requirements for lactation. The mineral metabolizable coefficient was quantified through observed (treatment means) mineral intake and total fecal and urinary excretions, discounting the estimated endogenous excretions from our proposed models. Endogenous excretion for P was derived from 130 treatment means of 39 balance trials (studies), and endogenous excretion for Ca was derived from 70 treatment means of 23 balance trials (studies). Estimated metabolizable coefficient for phosphorus was derived from 157 treatment means, and for Ca from 81 treatment means. Therefore, the differences in approach, and dataset size and amplitude between our proposed system and NASEM (2021) and NRC (2001) models are evident.

.

**The title does not adequately describe the manuscript. Consider revising. e.g. “A new system to predict phosphorous and calcium requirements in dairy cows”**
Author Response: We did not agree with your comments. The title adequately describes our study “A new system of phosphorus and calcium requirements for lactating dairy cows”

**The authors should ensure that any studies published after 6 November 2017 but before 1 January 2018 have not been accidently overlooked due to the nature of the follow up search term. I suggest that the year 2017 be included in the search term instead of the year 2018 and any duplicated studies in 2017 be removed.**
Author Response: We did not find articles that meet our inclusion criteria between November 6 and December 2017 search term.

**Figure 1: Instead of using the headings “Identification of previous studies via databases” and “Identification of new studies via databases”, I would suggest the authors refer to these searches as initial and follow up searches. Dates could be included to avoid any ambiguity. Double check the use of brackets and spacings in Figure 1. Please double-check the numbers in the screening section of the PRISMA diagram, there seem to be some inconsistencies?**
Author Response: The Figure 1 was unformatted during the creation of the TIF file. The TIF file was re-done. Thank you.

**The number of mineral balance trials included is not clear as the text and Table 1 state 76 trials however, there are only 72 trials included in the Excel file containing the complete dataset (reference 21), please clarify.**
Author Response: Please note that some articles reported more of one balance trial (studies). Therefore, our complete dataset has correctly 57 articles with 76 balance trials (Table 1; Excel file [21]).

**Figure 2: Please update this figure to include the word excretion in the y axis label e.g. Total P excretion… and Total Ca excretion… to avoid any confusion.**
Author Response: The word “excretion” was added. Thank you.

**Figure 3: Please update this figure to include the word excretion in the x and y axes labels e.g. Observed total P excretion… and Observed total Ca excretion… to avoid any confusion. In Figure 3 it is stated that 5 folds were created by study grouping to guarantee independence between observed and predicted values. According to S2, the folds were created on Monday 6 September 2023 but the follow up literature search was conducted in March 2024 – I imagine that no new studies were published and included in the meta-analysis between these dates, however, this should be explicitly stated (in the text rather than in the figure legend) for clarity.**
Author Response: The word “excretion” was added. You are right about the dates. We have updated this information in material and methods for clarity. Thank you.

**Table 2: Please include the word “analysis” in the heading for clarity i.e. Nonlinear mixed regression analysis…**
Author Response: The word “analysis” was added. Thank you.

**S3: Please double check that these graphs are correct? If so, please label with a term other than “dotted lines” to improve clarity, I do not see any dotted lines(?).**
Author Response: You are right. We have corrected for “solid lines”. Thank you.

**Figure 4: What does the X mean in Figure 4? Please add to the figure legend for clarity.

Figure 5: Include the meaning of X for clarity i.e. X represents the mean concentration. Label Holstein and Jersey on the x axis to avoid any confusion.**
Author Response: X represent breed as presented in Figure Legend. Thank you.

**Discussion

Figure 6: These are nice graphs that help the reader to visualize requirements predicted by different entities. Since the equations and absorption coefficients have been included for the other models, I suggest including the same for the authors proposed model. It is not entirely clear when looking at the figure that the authors are referring to 10kg milk yield/ 20/ 30 etc. Please include “milk” or “milk yield” or an abbreviation e.g. “MY” in the figure.**
Author Response: Thank you for your positive comments. The MY abbreviation was added in the Figure 6.

**Lines 393 – 395: This is a repetition from the introduction as it is, however, it is a good point and should be expanded upon in the discussion.**
Author Response: Thank you for your positive comments. The current discussion about this point is enough, and no expansion is needed.

**Lines 415 – 416: This may be correct, however, it is not deducible from Figure 6 as there is no indication of DMI or the equation used to derive these values related to Figure 6? Please clarify either in the figure, the legend or in the text.**
Author Response: We have improved the clarify this analysis. Thank you.

**Line 421: When referring to dietary phosphorous or calcium intake, add the word “dietary” before P (or Ca) e.g. “dietary P” to avoid any ambiguity. Please check this throughout the manuscript.**
Author Response: We have checked this issue throughout the manuscript. Thank you.

**Line 426: Are there really so few dose-response relationship studies on Ca and P in dairy cows? If so, I would make this very apparent in the introduction and then build on this in the discussion (as the authors have done to a certain degree), however, more compelling wording and insight is required for an improved discussion.**

Author Response: To the best of our knowledge, dose-response datasets from trials with lactating cows are limited, which restricts our ability to evaluate our models and other for predicting dietary P and Ca requirements for lactating dairy cows. We have addressed this issue the past and current version in our manuscript. Additionally, please note that this limitation is highlighted in discussion section. We believe that the current discussion adequately address this issue, and an expansion is unnecessary.

**Lines 428 – 430: Please recalculate these percentages, there seems to be an error. Additionally, it would make it easier for the reader if the authors referred to a specific milk yield and then another e.g. “Our proposed model predicts a net calcium requirement 117% higher than the NASEM and 79% higher than the NRC recommendations for a 500kg dairy cow producing 10kg of milk per day. For cows producing 50kg (700kg) of milk a day, our model predicts….”
Lines 432 – 433: To enhance clarity rewrite in the same fashion as suggested above.
Lines 434 – 437 need to be rewritten to provide clarity.**
Author Response: The differences (%) were recalculated. The sentences were changed for improve clarify. Thank you.

**Line 449 – 450: Mention which other factors are involved in endogenous fecal losses for completeness.**
Author Response: A mention which other factors are involved in endogenous fecal losses was included. Thank you.

**Line 453: Use “actual” for known MC instead of specific true**
Author Response: It was changed. Thank you.

**References: Please use the same referencing style throughout. See references 10, 12, 16, 17 (I have not been through all the references) for examples where the referencing style deviates from the majority.**

Author Response: References were revised. Thank you.

Sincerely,

André Oliveira

Corresponding Author
