# Supplementary material for: A new system of phosphorus and calcium requirements for lactating dairy cows
Source: PLoS One. 2024 Aug 29;19(8):e0308889. doi: 10.1371/journal.pone.0308889 (PMC11361663; doi:10.1371/journal.pone.0308889)
Supplement: Attachment — Submitted filename: Response to Reviewers.doc [file pone.0308889.s006.doc]

Dear Dr Aziz ur Rahman Muhammad,

Academic Editors

PLOS ONE

Thank you to review our manuscript PONE-D-2409154, “A new system of phosphorus and calcium requirements for lactating dairy cows”.

I have addressed the reviewer comments, and improved and provide recommendations for further studies in mineral nutrition for lactating cows, as your solicitation

Sincerely,

André Oliveira

Corresponding Author

**Response to Reviewer #2:**

Dear Reviewer,

Thank you for your comments, criticisms, and suggestions.

Please find below responses for your questions.

**Reviewer #2 comments: Different statistical approaches, animal genotypes, climate, and research protocols change over time. Allmeta-analyses have limitations, but we need to clearly state them (statistical methods, software used, and data availability). One interesting discussion during the 2024 ADSA meeting this year was about variation in NDF analysisdue to differences in lab analysis. I believe that this type of discussion is important for mineral nutrition too.Over time, several research groups have been discussing how to improve our Dairy Science or Modeling Science.This reviewer made some points about methods for weighting studies in meta-analysis (SEM), nonlinear mixed-effectsmodels, cross-validation, and other aspects to encourage the authors to discuss ways to improve our meta-analysisapproach. Discussing the limitations and benefits of current meta-analysis methods and data available can promptreaders to think about these challenges, how this can affect the results, and address them in future research. A popular method widely used in meta-analysis doesn’t mean it cannot be improved. This reviewer respectfully disagrees with SEM being considered a gold-standard method, although it is widely used inAnimal Science meta-analysis. SE from GLM and Mixed models are fitted differently; SE from GLM is largelyunderestimated. If not evaluated carefully, this can cause under- or over-weighting in the model (see the discussion byHanigan et al., 2021, 2024: https://doi.org/10.3168/jds.2020-19672 , https://doi.org/10.3168/jds.2024-24230 ). This reviewer agrees that this type of discussion is more appropriate for a statistical methods paper. However, if theauthors choose to use this method, they need to justify it.**

Author Response: Thank you for sharing your insight on modelling, and the discussion during the 2024 ADAS meeting. You are right about the limitation of SEM as weighting criteria of studies when are fitted with different models (mixed and fixed). However, please note that I have addressed this issue since first version through of the normalized 1/SEM for mixed and fixed, according to a suggestion of St-Pierre N.R. (Former Professor of The Ohio State University). When 1/SEM is normalized the values was around to 1. How I did it separately for fixed and mixed models, the limitation of underestimation SEM from fixed models was addressed.

“Weighting factor = W_1_/W_2_, where: weighting factor = normalized inverse of the SEM of mineral fecal excretion (g/d); W_1_ = 1/SEM mineral fecal excretion (g/d); and W_2_ = overall mean of W_1_ across studies. To prevent overweighting of studies with extremely low SEM [23], we truncated (i.e., trimmed) the SEM in 0.35 × overall mean SEM; then SEM < (0.35 × overall mean SEM) was trimmed at 0.35 × overall mean SEM of mineral fecal excretion. This analysis was conducted separately for the studies that adopted mixed and fixed effects models because mixed models tend to have higher SEM [22, 24].”

**Reviewer #2 comments: This reviewer appreciates the biology discussion, framework description, and codes, which are acceptable in science. The major questions for the conclusion section are: do we need a new system or do we need recent/new studies in mineral nutrition for lactating dairy cows? The authors can discuss the N of papers published in the last 5 years intheir database. Also, please include recommendations for further research/meta-analysis.**

Author Response: Thank you for your positive comments. My motivation is that the NASEM (2021) recommendation of requirement for maintenance and AC are based in an analysis of limited dataset and from different approach that I have proposed here. Please, note that a recommendation for further studies are added since first version of manuscript “.. the adequacy of our proposed system predicting dietary P and Ca requirements and other models, such as the NASEM (2021) and NRC (2001), still requires further evaluation through independent response-dose feeding experiments”.

However, I have added a new recommendation in conclusion, as your solicitation. “In addition, our model can likely be improved through future studies that evaluate dietary, animal and environmental variables affecting mineral endogenous excretions and metabolizable coefficients.

Sincerely,

André Oliveira

Corresponding Author

**Response to Reviewer #3:**

Dear Reviewer,

Thank you for your detailed review.

Please find below responses for your questions.

**Reviewer #3: Dear Authors, thank you for addressing my previous concerns to a large extent, however, there a still quite a few small grammatical errors that need to be addressed before publication. I’ve made a list of the mostobvious ones below but please have a native English speaker go through your manuscript once again. I’ve advised toaccept your manuscript once these minor issues have been addressed. Thank you for an interesting read.L45: Add the word “the” before “metabolizable coefficient”L71: Should read “mineral balance” instead of “balance mineral” trials…L88: “model of” should read “models for”L90: Use “requires development” instead of “is still necessary to be developed”L94: “of” P and Ca… should read “for”L95: “nutrients” should be singularL109: “requirement” should be pluralL118 and L121: “peer review” should read “peer reviewed”L218: Add the word “an” before “iterative approach”L236: “of” should be “from”L270: Remove the word “cows” from this sentenceL272: “of” should read “from”L281: “cow” should be singular and “dataset” pluralL282-283: Do you mean the parity was not reported in 34 studies? If so, please change the wording of this sentence.Table 3: Do you mean “Dietary starch” by “Starch diet?” If you use “Dietary starch” or simply “Starch”L390: The fullstop (period) should be a comma in this sentenceL407: “mechanism” should be pluralL419: Here “metabolizable” should read “metabolizability”L427: “the” should read “that”L499: “to adequately” instead of “adequately to”L501: “requirements” should be singularL502: remove “of Ca”L503: First “cows” should be singularL509: “requirements” should be singularL512: “requirements” should be singularL513: Replace “Because od” with “Due to the”L527: “sampling spot” should read “spot sampling”L527: “are evidences” should read “is evidence”L529: “to” should read “for”L530: “collect” should read “collection”**

Author Response:. All error were addressed. Thank you.

**Reviewer #3: Figure 1: Missing opening brackets for “Phosphorus”. Missing closing bracket in first box. Please revise.**

Author Response: The Figure 1 was reviewed. Thank you.

**Reviewer #3: Figure 4 has not been updated. My previous comment: Figure 4: What does the X mean in Figure 4? Please add to the figure legend for clarity.**

Author Response: Please note that this Figure is a box-plot. X represents the mineral group ( phosphorous and calcium) as presented in X-axis and legend. No updated is needed for clarity. Thank you.
